# Unusual prophages in *Mycobacterium abscessus* genomes and strain variations in phage susceptibilities

**DOI:** 10.1371/journal.pone.0281769

**Published:** 2023-02-16

**Authors:** Elizabeth D. Amarh, Rebekah M. Dedrick, Rebecca A. Garlena, Daniel A. Russell, Christian H. Gauthier, Haley G. Aull, Lawrence Abad, Deborah Jacobs-Sera, Chidiebere Akusobi, Eric J. Rubin, Graham F. Hatfull

**Affiliations:** 1 Department of Biological Sciences, University of Pittsburgh, Pittsburgh, PA, United States of America; 2 Department of Immunology and Infectious Diseases, Harvard T.H. Chan School of Public Health, Boston, MA, United States of America; Cornell University, UNITED STATES

## Abstract

*Mycobacterium abscessus* infections are relatively common in patients with cystic fibrosis and are clinically challenging, with frequent intrinsic resistance to antibiotics. Therapeutic treatment with bacteriophages offers some promise but faces many challenges including substantial variation in phage susceptibilities among clinical isolates, and the need to personalize therapies for individual patients. Many strains are not susceptible to any phages or are not efficiently killed by lytic phages, including all smooth colony morphotype strains tested to-date. Here, we analyze a set of new *M*. *abscessus* isolates for the genomic relationships, prophage content, spontaneous phage release, and phage susceptibilities. We find that prophages are common in these *M*. *abscessus* genomes, but some have unusual arrangements, including tandemly integrated prophages, internal duplications, and they participate in active exchange of polymorphic toxin-immunity cassettes secreted by ESX systems. Relatively few strains are efficiently infected by any mycobacteriophages, and the infection patterns do not reflect the overall phylogenetic relationships of the strains. Characterization of these strains and their phage susceptibility profiles will help to advance the broader application of phage therapies for NTM infections.

## Introduction

Non-tuberculous mycobacteria (NTM) are an increasingly common cause of disease including pulmonary infections in patients with cystic fibrosis (CF) and non-CF bronchiectasis, as well as extrapulmonary infections of skin and soft tissues [1]. Among the most common pathogens is *Mycobacterium abscessus* which can be divided into three subspecies, subsp. *abscessus*, *bolletii*, *and massiliense* [2]. Environmental isolates of *M*. *abscessus* typically have a smooth colony morphology and characteristically are able to form biofilms, perform sliding motility, and are characterized by abundant glycopeptidolipids (GPLs) in their cell wall. Clinical isolates may also have a smooth morphotype, but rough colony morphotypes are common, which exhibit clumping and cording, and loss of GPLs from the cell wall [1].

Clinical isolates of *M*. *abscessus* are often intrinsically resistant to antibiotics, and resistance can be extended further in response to antibiotic therapies [3]. Clinical management of *M*. *abscessus* infections usually requires multiple antibiotics–including intravenously administered and inhaled therapies–for extended periods of time, and drug toxicities present major challenges [4]. Although *M*. *abscessus* infections may be only part of a complex polymicrobial community in the lungs of CF patients, it is often a contrary indicator for lung transplantation, which might otherwise be a suitable clinical option [5]. This is due in part to concerns for disseminated infection associated with immunosuppressive drugs, a common concern for extrapulmonary NTM infections with immunosuppression for a variety of clinical conditions. Clearly, there is a need for new therapeutic strategies for NTM infections [6].

Lytic mycobacteriophages present a plausible new approach to controlling NTM infections, and a three-phage cocktail was used to treat a pediatric CF patient with a disseminated *M*. *abscessus* infection following bilateral lung transplantation [7]. The intravenous (IV) treatment was well-tolerated with minimal safety concerns, and clinical improvement was observed without emergence of phage resistance [7]; anti-phage antibody responses were not observed, presumably due to transplant-associated immunosuppression. Twenty compassionate-use phage treatments have now been reported, with favorable outcomes in at least eleven patients, and without observable phage resistance, even in patients treated with just a single phage [8–10]. Neutralizing antibody responses are common following IV administration, and may be associated with treatment failure in some cases [11, 12], but in others do not appear to prevent favorable outcomes [8–10]. The broader use of phages for treating *M*. *abscessus* infection is complicated by the necessity to screen individual clinical isolates for phage susceptibilities, as relatively few therapeutically applicable phages have been identified, and there is substantial variation in the phage susceptibility profiles [8, 13].

Students and faculty in the Science Education Alliance Phage Hunters Advancing Genomics and Evolutionary Science (SEA-PHAGES) program [14–16] have isolated over 12,000 individual phages using *Mycobacterium smegmatis* as a host; over 2100 have been completely sequenced and annotated [17]. These span considerable sequence diversity but can be grouped into clusters of sequence-related phages [18, 19]. Currently there are 31 clusters (Clusters A–AE) and seven singletons, each of which has no close relatives. Numerical representation varies greatly across these clusters with over 700 in Cluster A, but fewer than five in Clusters U, V, X, Y, Z, AA, AC, AD and AE [17, 20]. Many clusters are also diverse and can be divided into subclusters according to their sequence relationships, and Cluster A has twenty subclusters (Subcluster A1– A20). In general, the sequence boundaries between all these groups are diffuse, and it is likely there is a continuum of viral diversity in nature, albeit with unequal representation [21]. The majority of the mycobacteriophage sequence types (clusters, subclusters, singletons) are temperate, although they often include phages that are naturally occurring lytic variants. A small subset of these types infect other bacterial hosts including *M*. *tuberculosis* and *M*. *abscessus* [13, 22, 23].

Few phages have been isolated directly on any strain of *M*. *abscessus*, and those that have are generally relatives of *M*. *smegmatis* phages [7]. However, representative phages of Clusters G, A, K, and AB infect some *M*. *abscessus* strains and have been used therapeutically to treat NTM infections [7, 12]. However, the phage infection profiles of 82 clinical *M*. *abscessus* isolates are highly variable and are not predictable from bacterial genomic information alone. Some smooth colony morphotype strains are infected by one or more phages, but none are efficiently killed, and no therapeutically useful phages are available for these infections [13]. In contrast, 75% of rough colony morphotype strains are infected and killed by one or more mycobacteriophages [13], although these may need to be engineered to endow them with lytic properties [7, 24].

The determinants of mycobacteriophage host range remain unclear, few receptor candidates have been identified, and most if not all NTM strains are CRISPR-free [13, 25]. However, prophages are important contributors to host range with many examples of lysogenically expressed genes of prophages conferring defense against viral attack, both from closely related phages (homotypic defense, including superinfection immunity) and unrelated phages (heterotypic defense) [26–28]. *M*. *abscessus* strains vary greatly in their prophage content [29], and 85% of strains contain at least one integrated prophage, and can have as many as six [30]. Most of these prophages are unrelated to the phages of *M*. *smegmatis* but also span considerable sequence diversity and integrate into at least 18 different genomic attachment sites (*attB*) [13, 30]. Most are predicted to be viable and capable of lytic growth, and several spontaneously induced prophages have been propagated on permissive host strains [13, 31, 32]. It is plausible that the prophages–together with the variable plasmid content–contribute substantially to the variation in phage susceptibilities among different *M*. *abscessus* clinical isolates [13, 26].

Here, we explore the strain variation and phage infection profiles of 20 *M*. *abscessus* strains of different origins than those examined previously. Thirteen strains originate in Taiwan and six from the US, together with three type strains. Most of these strains have a smooth colony morphotype and few are infected by any of the *M*. *smegmatis* phages tested. However, 70% of the strains have at least one integrated prophage, most of which are intact and release virions into the culture supernatants. Further, some prophages are unusually arranged with tandem integrations or internal duplications.

## Results

### Phylogeny of *M*. *abscessus* strains

**Fig 1:**
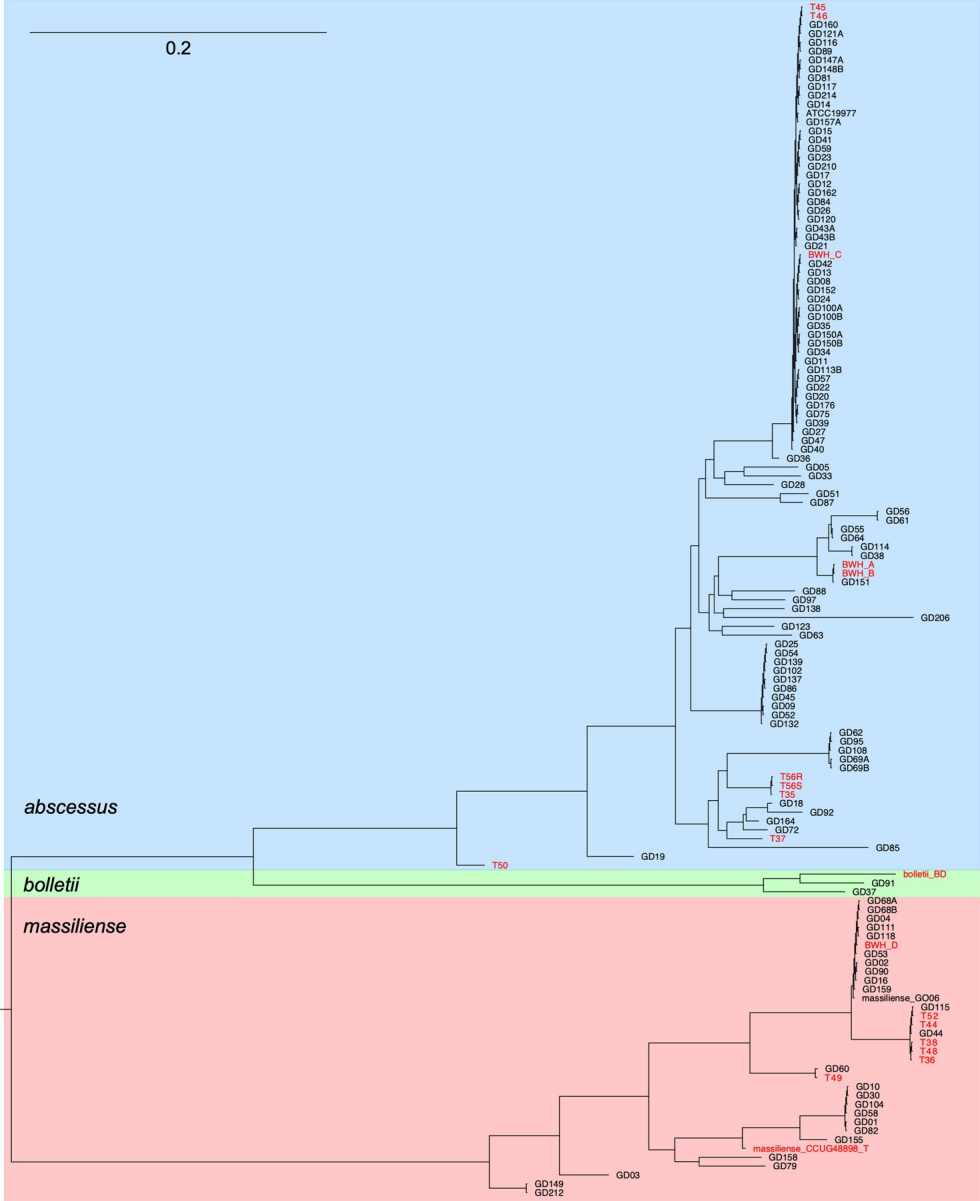
Phylogeny of *M*. *abscessus* strains. Phylogenetic relationships of *M*. *abscessus* isolates based on 3,350,221 (65.8%) positions conserved in all strains. Subspecies are shaded blue, green, and red for *M*. *abscessus* subsp. *abscessus*, *M*. *abscessus* subsp. *bolletii*, and *M*. *abscessus* subsp. *massiliense*, respectively. Strain names in red type are those discussed further here. Other strains either were reported previously [13] or are our unpublished data. Scale bar corresponds to 0.2 substitutions per position.

A set of 20 *M*. *abscessus* isolates were chosen for analysis of their subspecies designations, phylogenies, colony morphotypes, prophage content, and phage susceptibility patterns (Table 1 and Fig 1). All strains were whole genome sequenced to a modest number of contigs (S1 Table) and one (T36) was sequenced to completion (Table 1). Phylogenetic analysis reveals considerable diversity, with most (11) being subsp. *abscessus*, eight being subsp. *massiliense*, and one being subsp. *bolletii* (Table 1 and Fig 1). Three *M*. *abscessus* subsp. *abscessus* strains (T45, T46, and BWH-C) are in a major clade with ATCC19977 that is prominent among clinical isolates of *M*. *abscessus* [13, 33, 34] (Fig 1). Strain T50 is the most diverged subsp. *abscessus* strain from ATCC19977 and they differ at 29,458 positions (~0.6%). Five of the *M*. *abscessus* subsp. *massiliense* strains (T38, T48, T36, T44, and T52) are in a closely related clade with previously described strain GD44 and GD115 [13] (Fig 1). Most of the strains have a smooth colony morphotype, with the exceptions of T56R, BWH-D and CCUG50184-T (which is a re-sequenced assembly of bolletii_BD). Strains T56R (rough) and T56S (smooth) are derived from the same isolate and differ by only four substitutions, including a single base deletion in *mps1*, which likely accounts for the rough phenotype in T56R.

**Table 1:** *M*. *abscessus* strains and their prophages.

Strain	Source	R/S^1^	Seq^2^	Subsp^3^	Prophages	Coordinates^4^	Clust^5^	Other^6^
T35	Taiwan	S	WGS	a	None	N/A	N/A	None
T36	Taiwan	S	Com	m	prophiT36-1	3482056-3592847r	MabL	None
					prophiT36-2a	535146–575938	MabN	
					prophiT36-2b	575899–613553	MabB	
T37	Taiwan	S	WGS	a	prophiT37-1	C367 223812-300022r	MabK	MabC
T38	Taiwan	S	WGS	m	prophiT38-1	C1 448015-513273r	MabL	None
					prophiT38-2a	C8 262243-221451r	MabN	
					prophiT38-2b	C8 221490-183836r	MabB	
T44	Taiwan	S	WGS	a	None	N/A	N/A	None
T45	Taiwan	S	WGS	a	prophiT45-2	C393 19631–74641	MabJ	MabA1, MabB, MabL/G
T46	Taiwan	S	WGS	a	prophiT46-1	C3 260970–313843	MabG	MabA1, MabB
					prophiT46-2	C2 346212–411463	MabL	
					prophiT46-3	C5 19238–74248	MabJ	
T48	Taiwan	S	WGS	m	prophiT48-1	C1 952938–1018196	MabL	None
					prophiT48-2a	C7 34327–75119	MabN	
					prophiT48-2b	C7 75080–112734	MabB	
T49	Taiwan	S	WGS	m	prophiT49-1	C1 832090–898129	MabL	None
					prophiT49-2	C1 1081832–1162060	MabI	
					prophiT49-3	C3 393982–447365	MabJ	
T50	Taiwan	S	WGS	m	prophiT50-1	C387 35546–76736	MabB	Plasmid
T52	Taiwan	S	WGS	m	None	N/A	N/A	None
T56R	Taiwan	R	WGS	a	None	N/A	N/A	Plasmid
T56S	Taiwan	S	WGS	a	None	N/A	N/A	Plasmid
BWH-A	Boston	S	WGS	a	prophiBWHA-1	C10 143636-200007r	MabJ	Plasmid
BWH-B	Boston	S	WGS	a	prophiBWHB-1	C10 143836-200208r	MabJ	Plasmid
BWH-C	Boston	S	WGS	a	None	N/A	N/A	MabA1; plasmid
BWH-D	Boston	R	WGS	m	prophiBWHD-1	C2 272234–332665	MabE1	None
CCUG50184-T	CCUG	S	WGS	b	prophiCCUG50184T-1	C1 1340084–1405291	MabL	= bolletii BD
CCUG48898-T	CCUG	R	WGS	m	prophiCCUG48898T-1	C3 369722–419386	MabC	None
					prophiCCUG48898T-2	C7 228947–290995	MabA1	

^1^Colony morphotype. R, rough; S, Smooth

^2^Genome sequencing. WGS, whole genome sequence; Com, complete genome sequence

^3^Strain subspecies; a, subspecies *abscessus*; m, subsp. *massiliense*, b, subsp. *bolletii*.

^4^Prophage coordinates within the strain genome; WGS data analyzed for all but T36, contig number indicated by C. Prophage orientation is that of the host; coordinates followed by an “r” indicate prophage is in reverse complement orientation.

^5^Cluster of each prophage, based on designations in ref. [30].

^6^BLASTN searches indicate that some strains contain additional prophages, but their sequences are split between two or more contigs and cannot be extracted as a single prophage sequence; their predicted cluster designations are shown. Some strains contain a plasmid which is also noted.

### Identification of integrated prophages in *M*. *abscessus* genomes

To identify integrated prophages in the genomes of these *M*. *abscessus* strains, each was analyzed using PHASTER [35], DEPhT [36], BLAST [37], and other bioinformatic tools. Complete prophages were identified and could be bioinformatically extracted from eleven strains as well as the type strains of *M*. *abscessus* subsp. *bolletii* (CCUG50184-T) and *M*. *abscessus* subsp. *massiliense* (CCUG48898-T) (Tables 1 and 2). Some strains contain additional prophages which are split into different contigs in the WGS assemblies, and intact prophage sequences could not be readily extracted (Table 1). Four strains (T35, T44, T52, together with T56S and T56R) appear to be prophage-free. Some strains contain multiple prophages, with strain T46 carrying at least three (Tables 1 and 2).

**Table 2:** *M*. *abscessus* prophages.

Prophages^1^	Cluster^2^	Length (bp)	ORFs	tRNA/tmRNA	attB^3^	Int^4^	Comments^5^
prophiCCUG48898T-2	MabA1	62049	89	0	attB-20	Y	See S1 Fig
prophiT36-2b	MabB	37655	51	1	attB-2	Y	See Figs 3 and 4
prophiT38-2b							See Figs 3 and 4
prophiT48-2b							See Figs 3 and 4
prophiT50-1	MabB	41191	59	0	attB-2	Y	See S2 Fig
prophiCCUG48898T-1	MabC	49665	68	0	attB-13	Y	See S3 Fig
prophiBWHD-1	MabE1	60432	80	0	attB-4	Y	Same as prophiGD04-1
prophiT46-1	MabG	52874	79	0	attB-11	Y	See S4 Fig
prophiT49-2	MabI	80229	161	24; tmRNA	attB-17	S	See S5 Fig
prophiT46-3	MabJ	55011	82	2	attB-7	S	See S6 Fig
prophiT45-2							
prophiT49-3	MabJ	53384	73	2	attB-21	S	See Fig 8
prophiBWHA-1	MabJ	56372	91	2	attB-7	S	See S7 Fig
prophiBWHB-1							
prophiT37-1	MabK	76211	110	0	attB-1	Y	See S8 Fig
prophiT36-1	MabL	110792	129	0	attB-10	Y	See S5 Fig
prophiT48-1	MabL	65259	93	0	attB-10	Y	See S5 Fig
prophiT38-1							
prophiT46-2	MabL	65252	95	0	attB-3	S	See S9 Fig
prophiT49-1	MabL	66040	92	0	attB-10	Y	See S10 Fig
prophiCCUG50184T-1	MabL	65208	93		attB-10	Y	See Fig 5
prophiT36-2a	MabN	40793	54	0	attB-2	Y	See Figs 3 and 4
prophiT38-2a							See Figs 3 and 4
prophiT48-2a							See Figs 3 and 4

^1^*M*. *abscessus* prophages are designated as prophixxx, and identical prophages are listed indented. Identical prophage regions identified in strains T36 and T38, and T48 were designated as prophiT36-2, prophiT38-2, and prophiT48-2, respectively, but each contains tandemly integrated prophages, designated as prophiT36-2a and prophageT36-2b, with their identical relatives in T38 and T48 named accordingly.

^2^Cluster designations of prophages is according to the system described in ref. [30]

^3^The *attB* site occupied by the prophage is according to the designations described in ref. [30] and as shown in Fig 2.

^4^The prophage-encoded integrase (Int) is indicated as either a tyrosine-integrase (Y) or a serine-integrase (S).

^5^Detail genome maps are shown in figures or supplementary figures as indicated.

Comparison of the identified prophages with those described previously enables assignment of each to a cluster or subcluster (MabA–MabS) and no new singletons or representatives of new clusters were identified (Table 2). However, with the exception of the prophage prophiBWHD-1, which is identical to the previously described prophiGD04-1 [30], all vary–some substantially–from previously described *M*. *abscessus* prophages. Detailed genomic maps are shown in S1–S10 Figs (see Table 2). In general, the prophage genomes are organized similarly to those described previously [30], although there are many intra-cluster variations. All of the prophages–with the exception of the MabJ prophages–encode a polymorphic toxin-immunity system, genetically closely linked to one of the *attL*/*attR* sites (S1–S10 Figs and see below).

The prophage content in the closely related strains T36, T38, and T48 is the same, and prophiT38-1 and prophiT48-1 are identical to prophiT36-1, excluding an insertion present in prophiT36-1 that is absent from prophiT38-1 and prophiT48-1 (Cluster MabL; Table 2). All three strains also contain tandemly integrated prophages at the same location (e.g., prophiT36-2a and prophiT36-2b), and there are cognate prophages in strains T38 and T48, as described below; prophiT45-2 is identical to prophiT46-3 (Cluster MabJ), and prophiBWHA-1 and prophiBWHB-1 are also identical (Cluster MabJ) (Table 2). Five of the prophages code for tRNAs, typically one-to-two per prophage, excepting prophiT49-2 (Cluster MabI), which codes for 24 tRNAs and a tmRNA (Table 2).

### Use of *attB* sites in the *M*. *abscessus* genome

**Fig 2:**
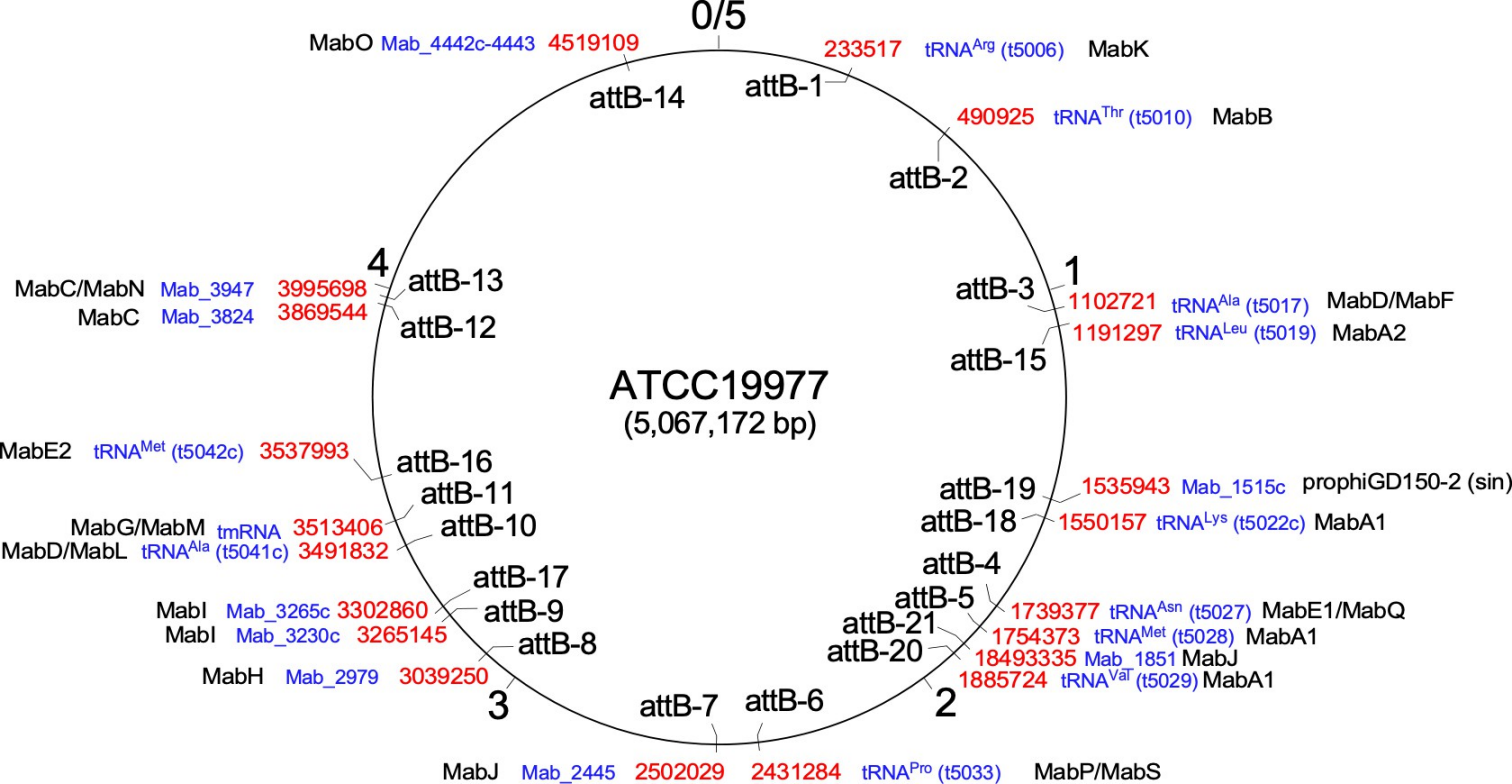
Locations of *attB* sites used by *M*. *abscessus* prophages. The *attB* sites in the *M*. *abscessus* ATCC19977 genome used by prophages characterized to date are shown. The 5 Mbp circular genome is represented, with the locations of 21 *attB* sites (attB-1 to attB-21) indicated within the circle. Text outside of the circular genome indicates the *attB* coordinates (red), the gene it is in (blue), and the cluster(s) or subcluster(s) of the prophages that integrate into that site (black). The figure is adapted from Fig 3A in reference [30], which also shows the positions of attB1 –attB-18. The attB-19 site is used by prophiGD150-1 which will be described elsewhere. The attB-20 and attB-21 sites are newly identified in this study (see Table 2).

Eleven of the prophages code for tyrosine-integrases and use seven different *attB* sites (Fig 2 and Tables 2 and S2). Six of these sites have been described previously, but one prophage (prophiCCUG48898T-2, identified in the type strain CCUG48898-T) integrates into a previously unidentified site designated *attB*-20, which overlaps a host tRNA^Val^ gene (equivalent to t5029 in ATCC19977). Five prophages use serine-integrases four of which integrate at previously identified *attB* sites (Tables 2 and S2). However, one–prophiT49-3 (Cluster MabJ)–uses a new site, designated *attB*-21, located within a protein-coding gene corresponding to MAB_1851, coding for a probable acyl-CoA dehydrogenase (FadE). Integration occurs around position 280 in the 1140 bp gene, likely inactivating its function, although the consequences of inactivating this gene on the physiology of *M*. *abscessus* is not known.

### Unusual tandemly-integrated prophages in strains *M*. *abscessus* T36, T38 and T48

**Fig 3:**
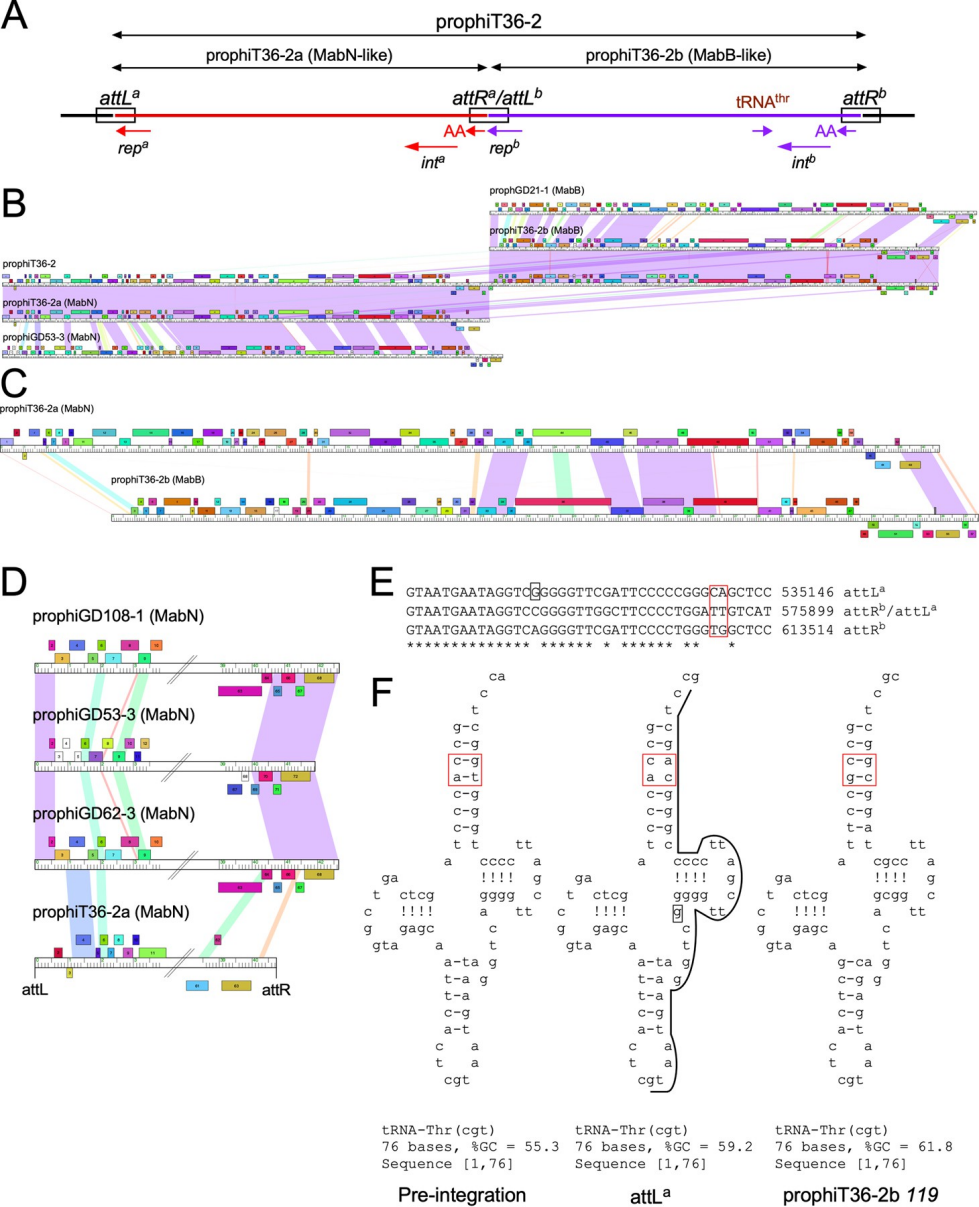
An unusual tandem prophage in *M*. *abscessus* T36. **A**. An overview of the proposed tandem prophage insertion, prophiT36-2a and b, in strain *M*. *abscessus* T36. ProphiT36-2a (red) is grouped into Cluster MabN, and prophiT36-2b (purple) is grouped into Cluster MabB. The putative attachment (*att*) sites are indicated as well as the repressor (*rep*) and integrase (*int*) genes. Both putative prophages use integration-dependent immunity systems in which the *attP* site is located within the repressor open reading frame of the phage, such that when integrated the 3’ end of the repressor gene coding for an *ssrA*-like degradation tag (shown as a short arrow with AA) is dissociated from the rest of the repressor gene (*rep*). **B.** Alignment of prophage genomes in the form of phamerator genome maps. Centrally positioned is the full phiT36-2 segment, and immediately above and below it are prophT36-2b and prophiT36-2a, respectively. At the top is the MabB prophage prophiGD21-1, and at the bottom is the MabN prophage prophiGD53-3 [30]. Genes are shown as colored boxes above and below each genome marker track, and pairwise nucleotide sequence similarity (from BLASTN) shown as spectrum colored shading between genomes with violet being the most similar; red indicates the least similarity above a threshold E value of 10^−4^. **C**. Comparison of the prophiT36-2a and prophiT36-2b genomes, illustrating region of nucleotide similarity. **D**. Genome alignment of the left ends of Cluster MabN prophages. Note that the prophiT36-2a genome ends are not closely related to those of other MabN prophages, the integrases are not closely related, and this prophage likely uses a different *attB* site for integration. **E**. Sequences of the common core at the *att* sites. Segments of 40 bp corresponding to the common cores are shown for the *att* sites shown in panel A. The boxed nucleotides correspond to differences between the pre-integration tRNA (equivalent to MAB_t5010) and the tRNA at *attL*^*a*^. **F**. Predicted secondary structures of the pre-integration tRNA (t5010), the tRNA at *attL*^*a*^, and the tRNA gene (*119*) encoded by prophiT36-2b. Red boxed regions correspond to those in panel E. The line around the tRNA at *attL*^*a*^ indicates the sequences contributed by prophiT36-2a common core.

*M*. *abscessus* T36 –together with T38 and T48 –contain tandemly inserted prophages, an unusual occurrence in all the *M*. *abscessus* strains we have analyzed; these prophages are identical in the three strains; all three are represented by prophiT36-2 in Fig 3. The entire prophage region (designated prophiT36-2) contains two different prophages integrated at the same site (attB-2, Fig 2), and these are designated prophiT36-2a and prophiT36-2b (Table 2). Comparison with other prophages shows that prophiT36-2a and prophiT36-2b are most closely related to those grouped in Clusters MabN, and MabB, respectively (Fig 3A and 3B). However, the two constituent prophages contain segments of nucleotide sequence similarity (Fig 3C), and there are multiple potential pathways that the tandem prophages could have been established. Perhaps the most plausible route involves homologous recombination between a superinfecting phage genome and a resident prophage, or a variation in which two phage genomes recombined via homologous recombination, which then integrated by integrase-mediated recombination. However, an alternative explanation is that a superinfecting phage integrated into a lysogen by integrase-mediated site-specific recombination between the incoming *attP* site, and either *attL* or *attR* of the resident prophage. Integrases are usually highly selective in their choice of *att* sites, but we provide support for formation of these tandem prophages through integrase mediated recombination.

**Fig 4:**
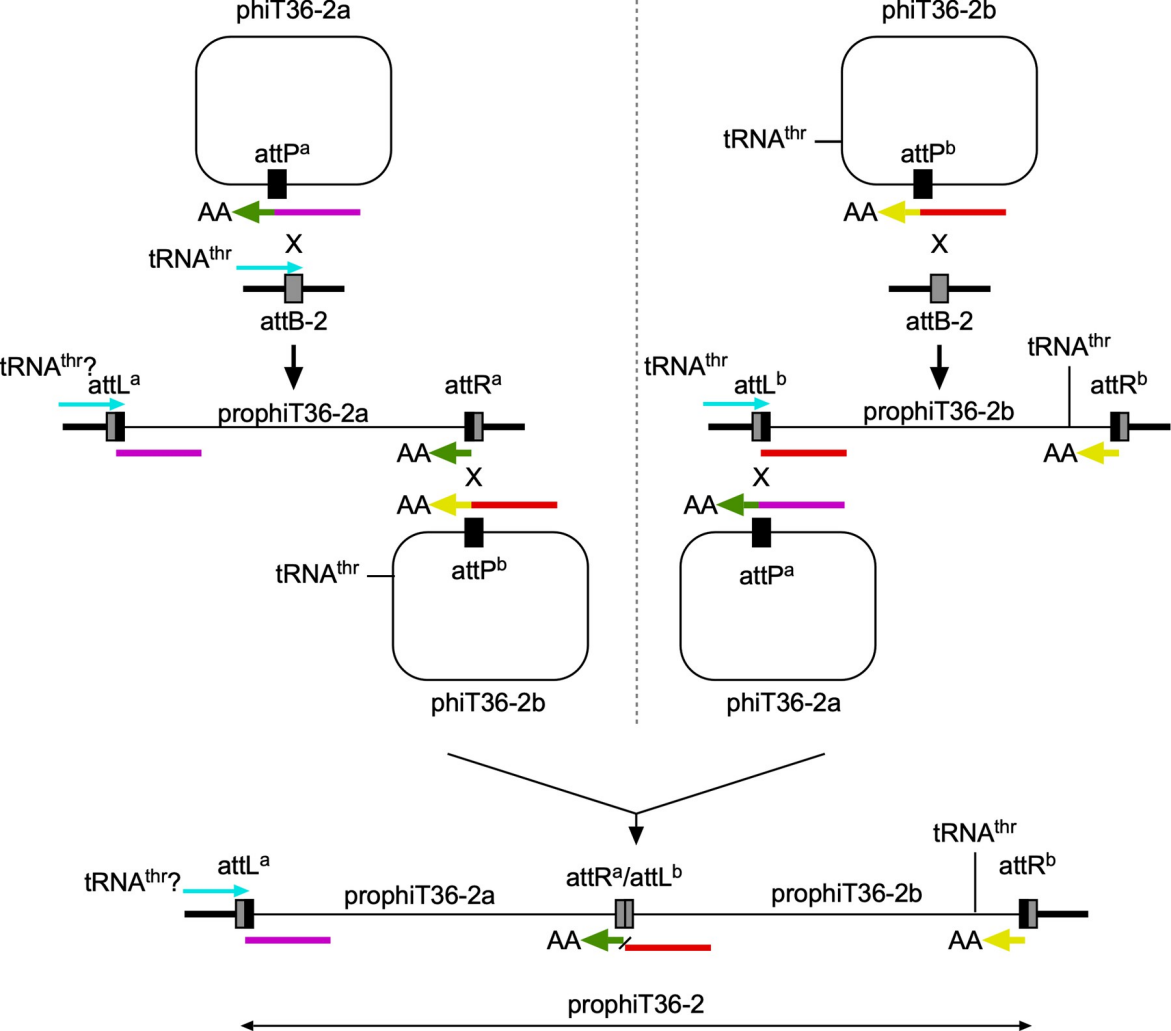
Pathways for formation of tandem prophages in T36. The prophiT36-2 prophage (bottom) is formed by prophiT36-2a (top left) and prophiT36-2b (top right). Both prophages can integrate at the *attB*-2 site. Additionally, both have integration-dependent immunity systems and the putative repressors for each prophage are not closely related. Formation of prophiT36-2 is likely to have occurred by two sequential site-specific integration events, although the order of events (either side of the dotted horizontal line) is unknown. On the left, an initial event occurs in which phiT36-2a integrates into the *attB*-2 site to form prophiT36-2a. The *attP* site (*attP*^*a*^) is located within the repressor gene, and the 5’ and 3’ ends of the repressor are shown in purple and green respectively; the extreme C-terminus contains two alanine amino acids (AA) as part of an ssrA-like degradation tag. Integrative recombination results in a stable and active form of the repressor (purple line) at *attL*^*a*^ and the 3’ remnant of the repressor gene at *attR*^*a*^. A second integrative recombination even between phiT36-2b and *attR*^*a*^ can then give tandem prophages. phiT36-2b also has an *attP* site within its repressor gene, such that integration results in the 3’ remnant of its repressor at the *attR*^*b*^ site and the active form of the repressor at the attR^a^/attL^b^ junction. Critically, although the *att* core sequences are closely related to each other, the repressors of phiT36-2a and phiT36-2b are translated in different reading frames, such that the 3’ remnant (green) of phiT36-2a is out of frame with the rest of the phiT36-2b repressor (red). If they were in the same reading frame, and thus fused in-frame at attR^a^/attL^b^ then the repressor would be unstable and inactive, and the lysogen non-viable. On the right side of the horizontal dotted line is shown an alternative series of events in which the phiT36-2b phage integrates first at *attB*-2, and then phiT36-2a integrates site-specifically by recombination between *attP*^*a*^ and *attL*^*b*^. Also shown is the approximate location of a tRNA^thr^ gene in prophiT36-2b that is absent from other Cluster MabB prophages. Note that the juxtaposition of the repressor and *att* common core sequences precludes an alternative scenario in which a phage initially integrates to form *attL*^*a*^ and *attR*^*b*^ followed by homologues recombination between a superinfecting phage and the resident prophage. Sequences of these regions are shown in S2 Fig.

We propose that the entire insertion (prophiT36-2) is composed of two prophages, each of which was derived from a phage, designated phiT36-2a and phiT36-2b (Figs 3A and 3B and 4). The occupied *attB*-2 sites overlaps a tRNA^thr^ gene (gene t5010 in *M*. *abscessus* ATCC19977 [30]), although T36, T38, and T48 are all subspecies *massiliense* (Table 1), and is used by other MabB prophages for integration, whereas all other MabN phages integrate at *attB*-13 [30] (Fig 2). However, prophageT36-2a differs at the nucleotide level from other MabN genomes at both extreme ends of the genome (Fig 3D), consistent with use of a different *attB* site; we also note that the prophiT36-2a integrase shares only 28% amino acid identity with other MabN integrases.

Comparison of the sequences at the prophiT36-2 boundaries identifies similar 40 bp sequences corresponding to the *att* site common core sequences (Fig 3E). These correspond to *attL*^*a*^ (left boundary), *attR*^*b*^ (right boundary), and the central *attR*^*a*^/*attL*^*b*^ junction site (which we propose represents the *attR*^*a*^ site of prophage prophiT36-2a and the *attL*^*b*^ site of prophiT36-2b) (Fig 3A and 3E). We note, however, that the cores are not identical and have several nucleotide substitutions relative to the others. The *attL*^*a*^ site overlaps the host tRNA^thr^ gene (t5010), but as a consequence of nucleotide deviations near the 3’ end of the common core (Fig 3E), the tRNA formed at *attL*^*a*^ has two unpaired positions in the tRNA acceptor stem, which likely impairs the functionality of the tRNA product (Fig 3F).

Prophages prophiT36-2a and prophiT36-2b share a feature of both MabN and MabB prophages in that they use integration-dependent superinfection immunity systems [30]. These systems have been previously described in detail for several mycobacteriophages [38] and are characterized by key features. First, the common core of the phage *attP* site is located within the coding sequence of the phage repressor gene, such that integrative site-specific recombination results in loss of the 3’ end of the repressor gene (see Figs 4 and S2); as such the prophage form of the repressor gene is typically located at *attL*, whereas the 3’ remnant of the repressor gene is located at *attR* [38]. Second, the prophage form of the repressor protein is smaller than the phage-encoded gene product, due to the presence of a stop codon introduced by bacterial sequences at or near the *attL* junction [38]. Third, the larger phage-encoded repressor differs functionally from the shorter prophage form because it contains a ssrA-like tag (including two C-terminal alanine residues; -----AA C-term) that promotes proteolytic degradation of the phage-encoded repressor protein (see Figs 4 and S2); integration removes the tag such that the prophage-encoded form is stable, active, and confers superinfection immunity [38]. We have identified the repressor genes for both prophiT36-2a and prophT36-2b, as well as the putative 3’ remnants of repressor genes at both *attR*^*b*^ and at the *attR*^*a*^/*attL*^*b*^ junction (Figs 3A and 4 and S2); both of the remnant’s code for ssrA-like tags, as expected.

Interestingly, the genes for these two repressors–which are unrelated at the amino acid sequence level–are in different reading frames with respect to the common core sequence, a unique arrangement because these *att* sequences must function both as integrase recognition sites and to encode for repressor amino acid sequences (S11 Fig). This has important consequences. First, if this were not true, then the repressor present at the *attR*^*a*^/*attL*^*b*^ site would be non-functional, because in-frame fusion of the prophiT36-2b repressor to the 3’ repressor remnant from prophiT36-2a would render it non-functional (see Figs 4 and S2). Second, this relationship excludes the possibility that *attL*^*a*^ and *attR*^*b*^ were derived from the same phage (and the same integration event) as reconstruction of the putative *attP* sites from these does not yield a full-length phage repressor gene. Only the *att* site combinations *attL*^*a*^ x *attR*^*a*^*/attL*^*b*^ and *attR*^*a*^*/attL*^*b*^ x *attR*^b^ can reconstitute phage-encoded repressors with ssrA-like tags (Figs 4 and S2). It is thus highly unlikely that homologous recombination was involved in forming these structures.

As noted above, a conundrum with the observed prophage structure is that the tRNA^thr^ gene formed at *attL*^*a*^ is likely impaired due to mismatches in the acceptor stem. Interestingly, prophiT36-2b codes for its own tRNA^thr^(cgt) approximately 2kbp from *attR*^*b*^; other MabB prophages do not carry this tRNA [30] (Fig 3A and 3F). The tRNA^thr^ location is in a region in which the surrounding genes are prophage-expressed [30] and we speculate that this tRNA complements the functional impairment of the tRNA^thr^ at *attL*^*a*^.

These observations are consistent with formation of the tandem prophage using two integrase-mediated site-specific integration events (Fig 4). It is plausible that phiT36-2b integrated first, followed by phiT36-2a site-specific recombination between the phage *attP* and *attL*^*b*^ (Fig 4, right). However, it is more likely that phiT36-2a integrated first, such that the impaired tRNA^thr^ gene at *attL*^*a*^ conferred a selective requirement for integration of phiT36-2b (Fig 4, left) that restores normal cellular growth. Because both phages use integration-dependent immunity systems, these events could only occur if the repressors are encoded in different frames, because of the presence of common *attP* core sequences within the repressor genes. It is thus not surprising that they are rarely observed.

### Variation among cluster MabL prophages

**Fig 5:**
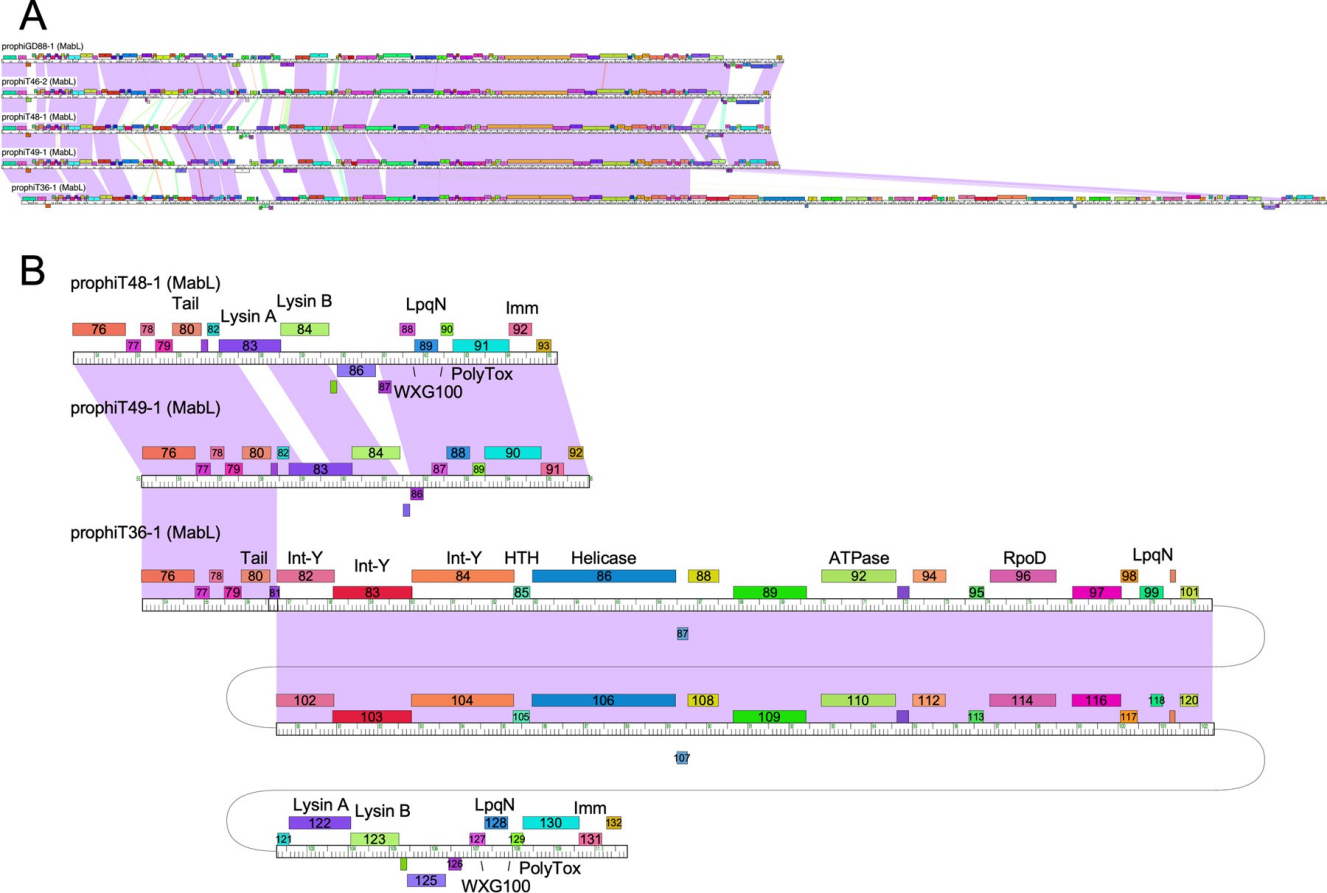
A large insertion in prophiT36-1. **A**. ProphiT36-1 is a member of Cluster MabL and is closely related to other cluster members. Five Cluster MabL genomes are shown and displayed in a phamerator genome map as in Fig 3. ProphiT36-1 is the 110, 792 bp genome at the bottom where the large insertion near the right end is evident. The alignment illustrates the global relationships, without emphasis on genomic details. **B**. The tandemly inserted 22.5 kbp DNA segment in prophiT36-1. The extreme right end of prophiT36-1 is shown aligned with prophiT48-1 and prophiT49-1. The insertion in prophT36-1 has been ‘wrapped’ such as to align the two repeated segments, which are identical to each other. Genomes are presented as in panel A, but with gene numbers shown with each gene box and putative functions indicated where known. Rightwards- and leftwards-transcribed genes are shown above and below genomes tracks, respectively. Note that the prophiT36-1 segment from genes *121* to *132* is closely related at the nucleotide level to prophiT48-1 genes *76*–*93*.

Bioinformatic analysis of the *M*. *abscessus* prophages suggests that most of them are likely intact and capable of forming lytically growing phages. However, propagating them lytically requires identifying a sensitive host strain, although this has been described for 11 phages, including phiT45-1 and phiT46-1 [13, 31, 32]. In one instance, a prophage (prophiGD20-1) contains a large transposon insertion which is predicted to make the genome too large to package, but a spontaneously released lytically growing phage was recovered in which the transposon had spontaneously excised [13]. The prophages described here appear to be mostly intact, although, as discussed above, one of the Cluster MabL prophages (prophiT36-1) has a peculiar, large insertion (Fig 5).

Five of the prophages group in Cluster MabL, although prophiT38-1 and prophiT48-1 are identical to each other (Table 2). The MabL prophages are closely related to each other and the previously reported prophiGD43A-6 and prophiGD88-1, with regions of variation encoding non-structural genes (Fig 5A). However, prophiT36-1 is over 45 kbp longer because of a large insertion between the tail genes and the lysis cassette (Fig 5B). The insertion contains a direct duplication of a 22.5 kbp segment, each containing 18 predicted protein-coding genes. One of these sets of genes appears to be typical tyrosine integrases (genes *82* and *102*), another has close similarity to tyrosine-integrases but with a C-terminal extension (*83* and *103*), and a third contain a small integrase-like domain but is globally distinct (*84* and *104*) (Fig 5B). Other genes of note are a DNA helicase (*86* and *106*), an AAA ATPase (*92* and *110*), and a gene containing both an RpoD-like sigma factor domain (*96* and *114*) and a motif related to TnsA transposases. Finally, a LqpN-like predicted lipoprotein is encoded by genes *99* and *118* (Fig 5B). This tandem insertion is anticipated to make the prophage too large to package, and likely arose from a recombination event–plausibly mediated by one of the tyrosine-integrase-like proteins–and we note that a second copy of the complete tandem duplication is present elsewhere in the *M*. *abscessus* T36 genome, situated between genes T36_00429 and T36_00464. Related copies are present in other *M*. *abscessus* subsp. *massiliense* genomes; we have not identified related insertions in any other prophages.

### Genetic exchange of polymorphic toxin-immunity cassettes

As noted previously [30], *M*. *abscessus* prophages commonly contain polymorphic toxin cassettes, which include an immunity protein that protects from toxicity, and a WXG-100 protein. These are rare among the *M*. *smegmatis* phages, although are present in several Subcluster K1 phages [17]. The polymorphic toxins typically also contain an N-terminal WXG-100 motif, and the toxin is likely exported via a Type VII secretion system [39, 40]. The prophages described here also contain similar cassettes, with the notable exception of the MabJ prophages (Table 2). Genome comparisons provide two examples of how these cassettes can be exchanged among different prophages.

**Fig 6:**
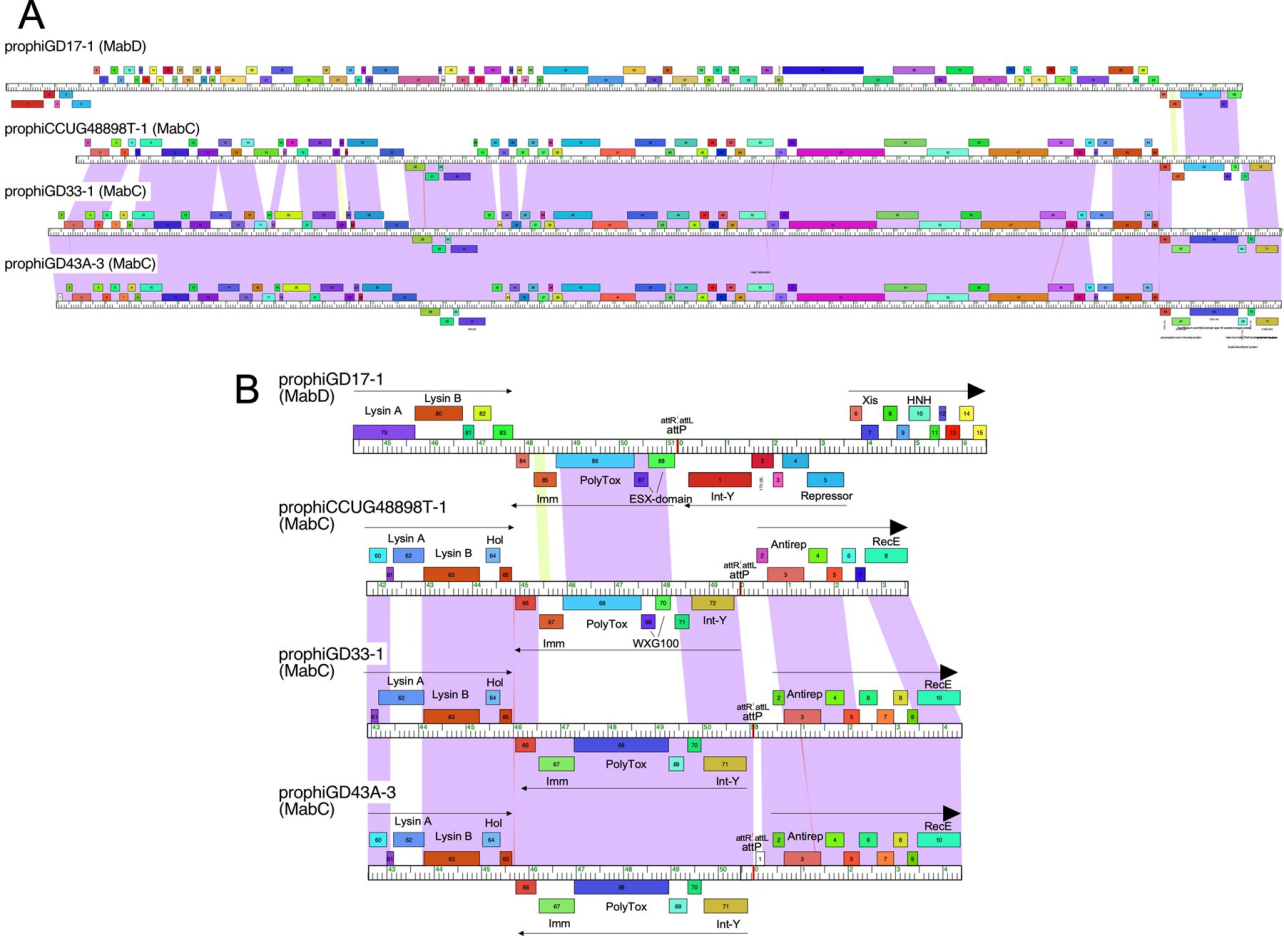
Swapping of polymorphic toxin-immunity systems between MabC and MabD genomes. **A.** Genome maps for prophiGD17-1, prophiCCUG48898T-1, prophiGD33-1, and prophiGD43A-3 are displayed. The top genome (prophiGD17-1) is in Cluster MabD, whereas the other three genomes are grouped into Cluster MabC. Genomes are displayed as in Fig 3. **B**. Exchange of the polymorphic toxin-immunity cassette between MabD and MabC phages. Genomes are represented as in panel A, with gene numbers shown inside gene boxes, and the directions of transcription noted by horizontal arrows. The color of each box reflects its gene phamily assignment, based on similar amino acid sequences [41]. Each genome segment shows the fusion of the extreme right end to the extreme left end of the genome, reconstituting *attP* and the organization as it would be represented in the parent phage genome. Note that MabC prophage prophiCCUG48898T-1 does not share nucleotide sequence similarity with the MabD prophage prophiGD17-1 except for genes *68*–*70*.

First, prophiCCUG48898T-1 is a close relative of other Cluster MabC prophages and does not have overall sequence similarity to MabD prophages such as prophiGD17-1 (Fig 6A). However, a 2.3 kbp region at the right end of the genome coding for genes *67*–*70* is unrelated to other MabC genomes, and genes *68*–*70* are closely related (97% nucleotide identity) to their homologues in the MabD prophage prophiGD17-1 (Fig 6B); prophiCCUG48898T-1 gene *67* is more distantly related to prophiGD17-1 gene *85* (77% nucleotide identity over the 3’ 50% of the genes). This cassette has thus been plausibly exchanged quite recently between MabC and MabD prophages, although multiple events were likely involved.

**Fig 7:**
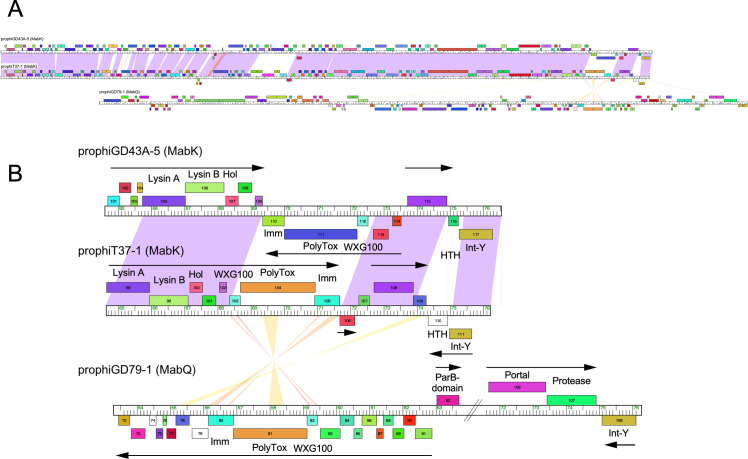
Swapping of polymorphic toxin-immunity systems between MabK and MabQ genomes. **A.** Genome maps are represented as in Fig 3. The top two genomes are both in Cluster MabK and are closely related as noted by the nucleotide sequence similarity shading between the genomes. The third genome (prophiGD79-1) is in MabQ and does not share extensive sequence similarity. **B**. Exchange of the polymorphic toxin-immunity cassette between MabK and MabQ phages. Genomes are represented as in Fig 3, with directions of transcription noted by horizontal arrows. Note that prophiT37-1 (MabK) has a different polymorphic toxin-immunity cassette from prophiGD43A-5 (MabK), but it is related to the cassette in the MabQ prophage prophiGD79-1.

The second case is illustrated by prophiT37-1, which is closely related to other genomes in Cluster MabK, and not closely related to Cluster MabQ phages (Fig 7A). In prophiT37-1, genes *103*–*105*, coding for the polymorphic toxin (*103*), immunity gene (*104*), and associated WXG100 gene (*105*) replace the cognate genes *110*–*112* in the MabK prophage prophiGD43A-5, but are transcribed rightwards; this rightwards transcription of the cassette is relatively unusual among *M*. *abscessus* prophages [30] (Fig 7B). These three genes are related to genes *80*–*82* of the MabQ prophage prophiGD79-1, although only distantly at the nucleotide level; amino acid identities are 54%, 38%, and 48% for prophiT37-1 genes *103*–*105* and their prophiGD79-1 homologues (Fig 7B). Although the specific roles of these polymorphic toxin-immunity systems are not known, their prevalence in these prophages and their propensity to exchange between different prophages suggests they are playing important roles.

### Identification of stoperator sites in MabJ prophages

The MabJ prophages described here are similar to the three (two of which are identical) MabJ prophages described previously [30]. The genomes of prophiT46-3 and prophiBWHA-1 indicate both use the same *attB*-7 site as these (Fig 2), located within the gene corresponding to MAB_2445. However, prophiT49-3 integrates into a new *attB*-21 site located within the gene corresponding to MAB_1851.

The MabJ prophages are organized similarly to the Cluster A phages isolated on *M*. *smegmatis* but are not closely related at the nucleotide sequence level [30, 42]. The Cluster A phages use an unusual repressor-operator system in which a small (~160–200 aa) repressor protein binds to a large number of binding sites located across the phage genome [43]. Each site is 13–14 bp long, is asymmetric, and positioned in one orientation relative to the direction of transcription; typically they are located within short intergenic regions, with a relatively high density near the right *cos* end. At least one of these sites acts as a true operator and overlaps a strong early lytic promoter, P_left_ [43, 44]. The other sites are proposed to act as repressor-dependent transcription termination sites and are referred to as ‘stoperators’ [45]. The MabJ prophages likely use similar systems, but these have not been explored.

**Fig 8:**
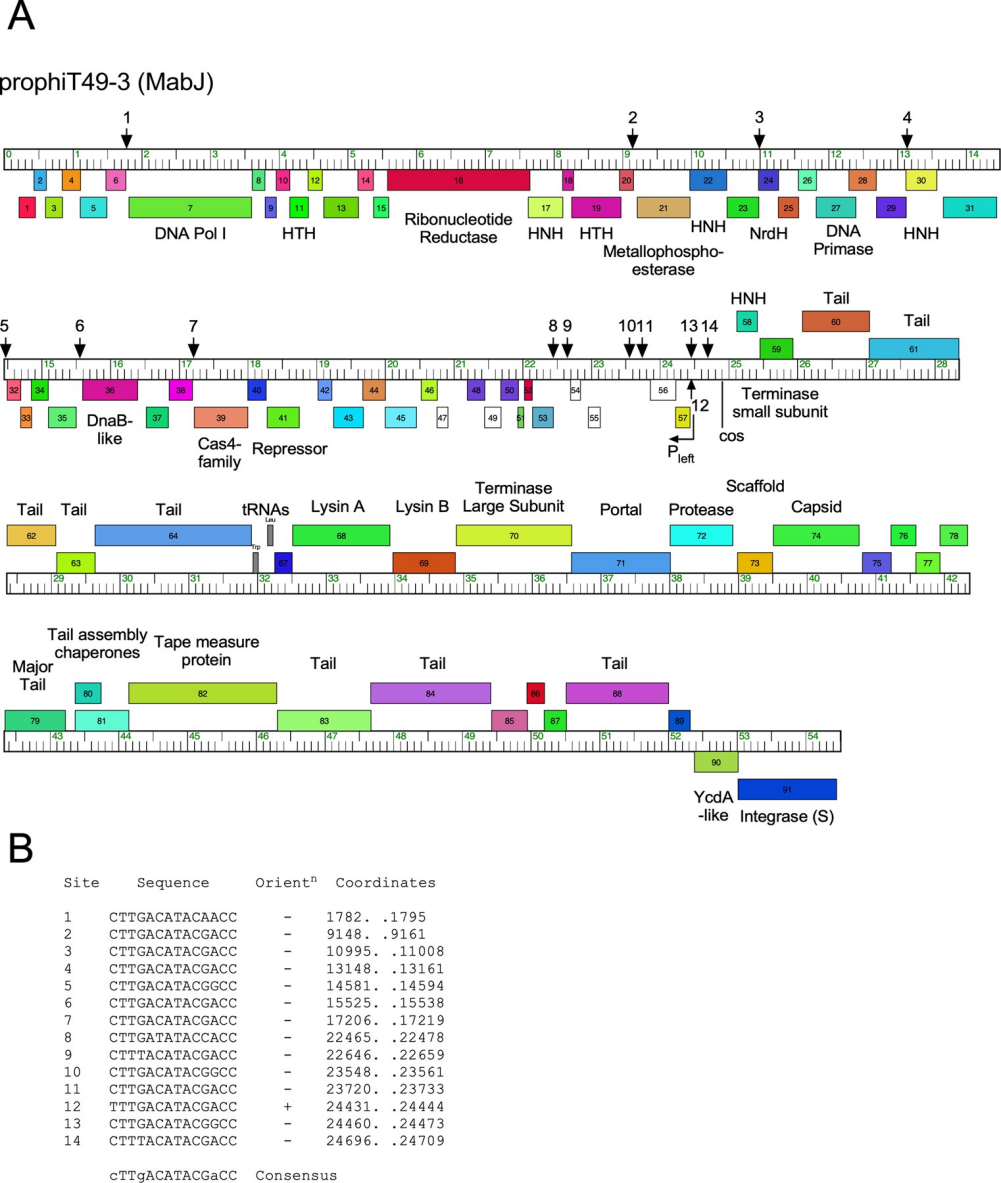
Repressor binding sites in MabJ prophage prophiT49-3. **A**. Genome map of prophiT49-3. The genes are displayed as described in Fig 3 and putative functions are shown, where known or predicted. The vertical arrows show the positions of predicted repressor binding sites. Site #12 overlaps the predicted early lytic promoter, P_left_, and is likely a true operator site. The other sites are all in the opposite orientation to site #12 and represent ‘stoperator’ sites where repressor binding is proposed to interfere with transcriptional elongation. **B**. Sequences of operator and stoperator sites showing their genome coordinates, orientation, and consensus sequence. Consensus positions conserved in all sites are shown in upper case type, and those present in at least ten of the sites are shown in lower case type.

A search of the prophiT49-3 genome identified a putative P_left_ promoter close to the predicted *cos* site, with features similar to P_left_ of phage L5 and other SigA promoters (Fig 8A). The repressor is encoded by gene *41* and the 155 amino acid product (gp41) is related to the repressors of the Subcluster A8 mycobacteriophage Expelliarmus and its relatives (40% aa identity). A search of stoperator/operator sites identified 14 sites with a consensus sequence 5’-cTTgACATACGaCC (Fig 8B). One of these (site #12) overlaps the predicted P_left_ promoter and presumably acts as the true operator site. The other sites are positioned predominantly between open reading frames and in one orientation relative to the direction of transcription, and there is a cluster of sites close to the right *cos* end (Fig 8A). There is some sequence variation among the MabJ repressors, but they appear to recognize similar stoperators to those shown in Fig 8B. In general, this unusual gene regulation system appears to be well conserved among the broader collection of Cluster A and MabJ phages, even though they span considerable overall diversity.

### Most prophages spontaneously induce to form phage particles

Most *M*. *abscessus* prophages appear to be full-length and not decaying or non-functional cryptic prophages, with the exception of those containing large insertions. To determine if the predicted prophages are released as virion particles into the culture supernatants through spontaneous induction, we determined if DNA products could be amplified using oligonucleotide primers flanking the predicted *attP* site (Table 1 and S12 and S13 Figs and S3 Table). Products were observed for many although not all of the prophages, consistent with the prediction that most of the prophages are functional (S3 Table). As predicted, products were not observed for the complete tandemly integrated prophages prophiT36-2, prophiT38-2, or prophiT48-2, as these would be too large to package. However, spontaneously induced virions from the constituent prophages (e.g. prophiT36-2a, prophiT36-2b, etc) were detected in culture supernatants, consistent with excisive recombination between attL^a^ and attR^a^/attL^b^ and between attR^a^/attL^b^ and attR^b^, as predicted (Fig 4 and S3 Table). Interestingly, we did observe a product resulting from attL^a^ x attR^b^ recombination (i.e. equivalent to prophiGD36-2) in bacterial culture, suggesting that this recombination event occurs, but does not yield virion particles. We did not observe *attP* products from prophiT45-2 either in liquid cultures or culture supernatants, although both were observed for the identical prophage prophiT46-3 (S3 Table). *attB* products were observed in some culture supernatants, potentially reflecting cell lysis due to prophage spontaneous induction.

### Phage sensitivities of *M*. *abscessus* isolates

**Fig 9:**
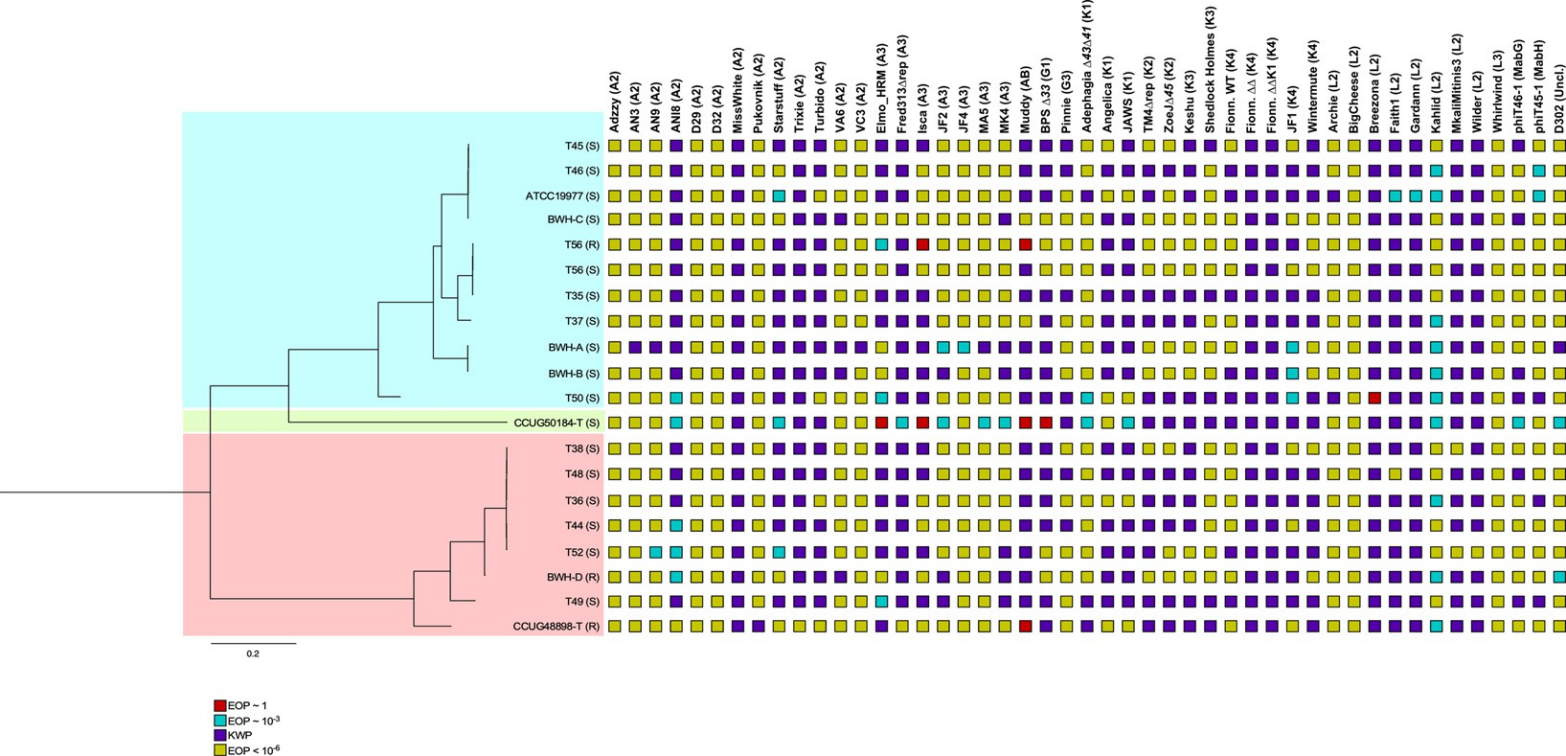
Phage susceptibility profiles of *M*. *abscessus* strains. *M*. *abscessus* strains and their phylogenetic relationships are shown at the left, together with the infection profiles of a broad set of phages. Subspecies *abscessus*, *bolletii*, *and massiliense* strains are shaded aqua, green and red, accordingly. Phage names are shown at the top, together with their cluster designation in parentheses. Phage infection is indicated according to the key below, according to the efficiency of plating relative to a control strain (*M*. *smegmatis* for most phages): Red box, EOP of 1, Aqua box, EOP of ~10^−3^, purple box, killing without plaquing (KWP), or yellow box, infection <10^−6^. Examples of these phenotypes are shown in S14 Fig.

Each of the *M*. *abscessus* strains was tested for susceptibility to a panel of 47 phages, including several representatives of genomic clusters/subclusters (A2, A3, AB, G1, G3, K1, K2, K3, K4, L2, L3); representatives of these cluster/subclusters have been shown previously to infect one or more *M*. *abscessus* strains [13]. Two lytically grown, spontaneously released phages, phiT45-1 and phiT46-1 [31, 32], as well as D302 (Fig 9) were also included in the screen. Ten-fold serial dilutions of each phage were spotted onto top agar overlays containing each *M*. *abscessus* isolate. After incubation, each strain was scored according to its efficiency of plaquing (EOP) relative to a control strain (Figs 9 and S14). Few strains are efficiently infected with any of the phages tested, although only a few strains have rough colony morphotypes, which are more susceptible to phage infection than their smooth counterparts [13]. CCUG50184-T, although smooth, is efficiently infected by phages Elmo_HRM^mc2155^, Isca_cpm (both subcluster A3), Muddy (Cluster AB) and BPsΔ*33* HTH (Subcluster G1). The two most common phenotypes are either no phage infection at all or clearing at high titer (lower dilutions) without formation of individual plaques, designated as the killing without plaquing (KWP) phenotype (Figs 9 and S14). However, a substantial number of interactions show plaquing at reduced frequencies of 10^−3^–10^−4^, with plaques likely originating from either phenotypic escape from restriction, or mutational escape from host or prophage-mediated viral defense systems [26, 30]. We note that the smooth and rough morphologies of strain T56 (T56S and T56R, respectively) differ in that Muddy and Isca_cpm efficiently infect T56R but not T56S. Overall, there is no evident correlation between prophage content and phage infection, and there are likely numerous defense genes determining phage susceptibilities in addition to the prophages.

### Phage susceptibility profiles of prophiT45-1 lysogens

Because prophages can confer viral defense systems, we tested whether prophiT45 [32] influences phage infection profiles of *M*. *abscessus*. The lytically propagated phiT45 was derived by spontaneous release from strain T45 previously [32], and although it does not efficiently infect any of the strains tested here, it does infect clinical isolates GD40, GD41 and GD245. We therefore constructed prophiT45-1 lysogens of these three strains and confirmed, by PCR, that the phage is integrated into each genome at the *attB*-10 site and that the lysogens are immune to superinfection by phiT45-1. The phage infection profiles of these new lysogens were compared to their parent strain, which doesn’t contain prophiT45-1 (S15 Fig). However, we observed no substantial differences in phage infection, and we conclude that either prophiT45-1 does not encode viral defense systems, or that any such defenses are highly specific, and we have not yet identified phages targeted by them.

## Discussion

Here, we characterized a phylogenetically diverse set of *M*. *abscessus* strains and showed that 75% of them contain at least one integrated prophage, similar to other *M*. *abscessus* strains [13, 29]. Comparison of the prophages to those previously described indicated that they were similar and no new clusters or singletons were identified, suggesting that there are few new *M*. *abscessus* prophage types to be discovered. Genomic analysis confirms the prophages to be full-length, intact, phage genomes. This is supported by PCR results indicating the phage *attP* sites are present in culture supernatants. However, there is substantial genomic variation within clusters, and this includes active exchange of the polymorphic toxin/immunity loci. Because most of the strains are whole genome sequence (WGS) assemblies in multiple contigs, we cannot exclude the possibility that some prophages escaped detection if located in gaps between contigs.

Most prophage genomes reflect a single integrated phage genome, but three closely related strains, T36, T38, and T48, all have identical tandem duplications of a Cluster N and a Cluster B prophage. The tandem duplications are not present in the genomically similar strains T52, T44, and T35 (Fig 1). Each tandem prophage was detected as a virion in the culture supernatant by PCR, indicating that each phage is virulent and functional (Table 3). Both of the constituent prophages use integration-dependent immunity systems and although these are complex, they facilitate dissection of the process by which these tandemly inserted prophages form. Determining how prevalent tandem prophages exist in the *M*. *abscessus* population is of interest.

**Table 3:** Presence of *attP* in cultures and culture supernatants.

Strain	Prophage	Cluster	attP; culture^1^		attP; s/n^2^	
			1	2	1	2
T36	prophiT36-1	MabL	+++	+++	+++	+++
	prophiT36-2	MabB	++	+++	-	-/+
	prophiT36-2a	MabN	++	++	+	++
	prophiT36-2b	MabB	+++	+++	++	++
T37	prophiT37-1	MabK	+++	+++	+++	+++
T38	prophiT38-1	MabL	+++	+++	+++	+++
	prophiT38-2	MabB	+	+++	-	-
	prophiT38-2a	MabN	+++	+++	++	++
	prophiT38-2b	MabB	+++	+++	++	++
T45	prophiT45-1	MabL	++	+++	++	++
	prophiT45-2	MabJ	-	-	-	-
T46	prophiT46-1	MabG	+++	+++	+++	+++
	prophiT46-2	MabL	+++	+++	+++	+++
	prophiT46-3	MabJ	+++	+++	+++	++
T48	prophiT48-1	MabL	+++	+++	+++	+++
	prophiT48-2	MabB	+	+++	-	-
	prophiT48-2a	MabN	+++	+++	++	+++
	prophiT48-2b	MabB	+++	+++	++	++
T49	prophiT49-1	MabL	+++	+++	+	++
	prophiT49-2	MabI	+++	+++	+++	+++
	prophiT49-3	MabJ	+++	+++	+++	+++
T50	prophiT50-1	MabB	-	+++	+++	+++
BWH-A	prophiBWHA-1	MabJ	+++	+++	+++	+++
BWH-B	prophiBWHB-1	MabJ	+++	-	+++	+++
BWH-D	prophiBWHD-1	MabE1	+++	++	++	*
CCUG50184-T	prophiCCUG50184-T-1	MabL	-	-	++	++
CCUG48898-T	prophiCCUG48898-T-1	MabC	-	++	-/+	-/+
	prophiCCUG48898-T-2	MabA1	-	++	+++	+++

^1^Amplification of the *attP* site by PCR in cell cultures is shown with product abundance indicated by–(absent), or with +, ++, or +++ reflecting increasing signal strength.

^2^Amplification of the *attP* site by PCR in filtered culture supernatants is shown with product abundance indicated by–(absent), or with +, ++, or +++ reflecting increasing signal strength. A barely detectable signal is shown as -/+.

Curiously, genome rearrangements in prophages do not necessarily mean they are defective and cannot produce spontaneously induced virion particles. This is illustrated by prophage prophiT36-1, which has a 22.5 kbp larger genome than its close relatives due to a duplicated insertion, but still releases virion particles with an *attP* site detected by PCR. It is plausible that phage-encoded recombinases promote recombination during spontaneous induction and thus resolve the duplication; prophiT36-1 encodes both RecE- and RecT-like proteins (gp15 and gp16, respectively). Similarly, it was shown previously that a prophage containing a large transposon insertion was able to form viable virions from which the transposon was lost [13]. These observations suggest that interpretations about the viability of bioinformatically-defined prophages should be made cautiously.

Although the *M*. *abscessus* prophages described here are not closely related to any of the previously described phages of *M*. *smegmatis*, the MabJ and MabI prophages are similarly organized to the Cluster A and Cluster M phages, respectively. The MabI prophage, prophiT49-2 (S5 Fig), has a similar genome size (~80 kbp), uses a serine-integrase for integration with an *attP* site distant from *int*, and contains a large set of tRNA genes, like Cluster M phages [46]. However, the prophiT49-2 genome contains a polymorphic toxin-immunity cassette, a common feature of *M*. *abscessus* prophages that is absent from *M*. *smegmatis* phages [13, 46]. Interestingly, prophages prophiT49-3, prophiT46-3, and prophiBWHA-1 (all in MabJ) are organized similarly to Cluster A *M*. *smegmatis* phages (with closest similarity to Subcluster A4 phages), including the characteristic feature of stoperator repressor binding sites throughout the ‘right arm’ of the genomes (Fig 9) [43]. However, the three predicted repressor proteins have substantially diverged, and they are unlikely to be homoimmune; it is unclear if this contributes to the phage infection profiles (Fig 9).

The variation in the phage susceptibility profiles of these strains is not particularly surprising, given previous reports of mycobacteria susceptibility to phage infection [7, 13]. However, the determinants of phage susceptibility remain largely unknown, and likely include a spectrum of previously described host defense systems [47], prophages, and plasmids [13]. Most of the strains tested here have smooth colony morphotypes, and the widespread resistance to phage infection is also consistent with prior reports [13]. Three of the strains are rough, however, and two of them are efficiently infected by Muddy, one of the most commonly used phages therapeutically (Fig 9) [8]. Interestingly, the *M*. *abscessus* subsp. *bolletii* strain CCUG50184T although smooth, is the most susceptible of all the strains infected here and is efficiently infected by four of the phages tested. This contrasts with the two *M*. *abscessus* subsp. *bolletii* strains tested previously (GD37 and GD91), neither of which are infected by any phages tested to-date [13]. *M*. *abscessus* subsp. *bolletii* strains are less common among the NTM pathogens, and there is a need to collect and characterize more of these strains to learn about their phage infection profiles.

## Materials and methods

### Bacterial strains and growth

Mycobacterial strains described here were received from the Eric Rubin Lab, Harvard University. Strains annotated as T## are clinically isolated strains from Taiwan. Strains annotated with the prefix “BWH-”are clinically isolated strains from the Brigham and Women’s Hospital located in Boston, Massachusetts. Bacterial cultures were grown in Middlebrook 7H9 liquid medium supplemented with oleic acid-albumin-dextrose-catalase (OADC), 1 mM CaCl_2_, carbenicillin (CB), and cycloheximide (CHX). Cultures were grown to OD_600_ of 0.4–1.0 at 37°C, on a shaker at 250 rpm. Top agar overlays were prepared on Middlebrook 7H10/CB/CHX solid medium with a mixture of Middlebrook Top Agar, 7H9/CH/CHX, and 1 mM CaCl_2_. Plates were incubated at 37°C for 4–6 days.

### Bacterial genome sequencing and phylogeny

DNA was extracted from bacterial strains by phenol-chloroform iso-amyl alcohol extraction. Sequencing libraries were prepared from genomic DNA using an NEB Ultra II FS kit with dual-indexed barcoding. Forty-eight libraries were pooled and run on an Illumina MiSeq, yielding single-end 150-base reads. These reads were then assembled using Newbler v2.9 with default settings, yielding a collection of contigs which was checked for completeness and using Consed v29 and as previously described [48]. Phylogenetic trees were generated using CSI phylogeny v1.4, a program which uses SNPs in WGS data to infer phylogeny. The program was run with the following parameters: 10x minimum depth at SNP positions, 10% minimum relative depth at SNP positions, minimum distance between SNPs disabled, minimum SNP quality score of 30, minimum read mapping quality score of 25, minimum Z-score of 1.96. Trees were visualized using FigTree v1.4.4. with the *M*. *abscessus* type strain ATCC19977 as a reference genome.

### Phage susceptibility screening

Ten-fold serial dilutions of phage lysates were spotted onto lawns of bacterial strains on solid media. Phage infections were scored by calculating efficiency of plaquing (EOP). EOP’s were calculated by dividing the titer (concentration) of phage observed on an NTM strain by the titer observed on the control strain *M*. *smegmatis* mc^2^155. An EOP = ~1.0 indicates the full infection phenotype; an EOP ~ 10^−3^ indicates the reduced infection phenotype; an EOP < 10^−6^, about zero, indicates no infection. The killing without plaquing (KWP) phenotype is apparent lysis in the presence of high concentrations of phage lysate, but without formation of individual plaques.

### Identification and extraction of prophage sequences

Bacterial genome sequences were analyzed for prophage content using PHASTER [35] and DEPhT [36], and the *attB* sites confirmed as described by Dedrick *et al*. [30]. Strain T36 used complete genome sequences and all other strains used WGS contigs. Prophage sequences were extracted and were analyzed and annotated using DNA Master v3.02, GeneMarkS v4.30 and Glimmer v3.02 as described previously [49]. Genome maps were constructed using Phamerator [41] with database Actino_Mab_4036. Protein functions were predicted using BLAST and HHpred [37, 50], and ARAGORN v1.2.41 [51] was used to identify tRNA genes.

### Detection of spontaneously released and integrated prophages by PCR

Spontaneously released prophages were detected by PCR amplification of the predicted *attP* sites. Bacterial cultures were grown to an OD_600_ = 0.6–1.0, and a 1 ml aliquot was separated by centrifugation at 17,000 rpm for 10 minutes. The supernatant was decanted into a syringe and filter sterilized through a 0.22 μM filter. 2 μl of culture supernatant were used for each PCR reaction, using primers described in S4 Table. Colony PCR was performed on picks from isolated colonies in the final phase of the T-streak plate for each clinically isolated strain.

### Construction of prophiT45-1 lysogens

Lysogens carrying a prophiT45-1 prophage were constructed by infecting strains GD40, GD41, and GD245 with phiT45-1 on solid media, and bacteria streaked out from the infected areas for single colonies. Colonies were purified by re-streaking, shown to carry an integrated prophT45-1 prophage integrated at *attB*-10 by PCR, and demonstrated to be immune to superinfection.

## Supporting information

S1 Table Genomic sequencing of *M*. *abscessus* strains. (DOCX)

S2 Table *attB* characteristics and coordinates in *M*. *abscessus* type strain ATCC19977. (PDF)

S3 Table Presence of *attP* and *attB* in *M*. *abscessus* cultures and culture supernatants. (PDF)

S4 Table Primers used in this study. (PDF)

S1 Fig Map of the prophiCCUG48898T-2 genome. The horizontal ruler represents the nucleotide sequence of the phage genome, with each bar indicating 1kb. Predicted genes are shown as colored boxes above and below the ruler, indicating rightward and leftward transcription, respectively. Genes are colored according to their phamily assignment using Phamerator [41] and database Actino_Mab_4036. Phamily designations with the number of members are shown in parentheses above each gene. Genes with no close relatives in this data set (orphams), are shown as white boxes. Predicted functions are shown above the genes.
(PDF)

S2 Fig Map of the prophiT50-1 genome. See S1 Fig for details.
(PDF)

S3 Fig Map of the prophiCCUG48898T-1 genome. See S1 Fig for details.
(PDF)

S4 Fig Map of the prophiT46-1 genome. See S1 Fig for details.
(PDF)

S5 Fig Map of the prophiT49-2 genome. See S1 Fig for details.
(PDF)

S6 Fig Map of the prophiT46-3 genome. See S1 Fig for details.
(PDF)

S7 Fig Map of the prophiBWHA-1 genome. See S1 Fig for details.
(PDF)

S8 Fig Map of the prophiT37-1 genome. See S1 Fig for details.
(PDF)

S9 Fig Map of the prophiT46-2 genome. See S1 Fig for details.
(PDF)

S10 Fig Map of the prophiT49-1 genome. See S1 Fig for details.
(PDF)

S11 Fig Sequences of *att* sites and repressor genes in prophiT36-2. Panels A-F show each strand of DNA sequence and the three translated reading frames that are transcribed leftwards. The 40 bp *att* common core sequence in each is shown in bold type. **A**. The sequence at the extreme left boundary of the prophiT36-2 prophage at *attL* (see Fig 3). The tRNA^thr^ gene is shown in aqua colored shading. The 3’ end of the repressor encoded by prophiT36-2a is shaded in purple. **B**. The sequence at the center of the prophT36-2 prophage that defines the boundary between prophiT36-2a and prophiT36-2b; it is proposed that the common core corresponds to *attR* of prophiT36-2a and *attL* of prophiT36-2b, designated *attR*/*attL*. The 3’ end of the repressor gene encoded by phrphiT36-2b is shaded in red. The predicted 3’ remnant of the phage-encoded form of the prophiT36-2a repressor is shaded in green. **C**. The sequence at the extreme right boundary of the prophiT36-2 prophage at *attR* (see Fig 3). The predicted at the extreme left boundary of the prophiT36-2 prophage at *attL* (see Fig 3) 3’ remnant of the of the phage-encoded form of the prophiT36-2b repressor is shaded in yellow. **D**. The predicted *attP* of phage phiT36-2a reconstructed from the sequences in panels A and B. Note that the 3’ remnant of the phage-encoded repressor contributed from *attR*/*attL* (green) is in-frame with the 5’ part of the repressor contributed by *attL* (purple). **E**. The predicted *attP* of phage phiT36-2b reconstructed from the sequences in panels B and C. Note that the 3’ remnant of the phage-encoded repressor contributed from *attR* (yellow) is in-frame with the 5’ part of the repressor contributed by *attR*/*attL* (red). **F**. Sequence of a potential *attP* that could be formed by site-specific recombination between the outside boundaries, *attL* and *attR*. In this case, the putative 3’ remnant of the repressor gene (yellow) would be fused out of frame with the rest of the repressor (purple), and thus these *attL* and *attR* sites are not derived from the same phage.
(PDF)

S12 Fig Detection of phage virions by PCR. **A**. Schematic of the primer annealing locations denoted with right and left facing half-headed arrows for the forward and reverse primers respectively. The line represents the host chromosome, with *attB* indicated. The rectangle depicts the integrated prophage flanked by *attL* and *attR*. **B**. PCR products using primers flanking the predicted *attP* from the identical prophages prophiT36-2, prophiT38-2 and prophiT48-2. For many prophages, two primers sets were used, designated 1 (primer set 1) and 2 (primer set 2) above the lanes. The input sample for the PCR was either from a bacterial culture (C) or a culture supernatant (S) as indicated. **C**. Schematic of the primer annealing locations, denoted with half-headed arrows as in panel A, for the 2a-2b *attR*^*a*^*/attL*^*b*^ site and the *attR* site of prophages prophiT36-2, prophiT38-2, and prophiT48-2. **D**. PCR products using primers amplifying 2a-2b *attR*^*a*^*/attL*^*b*^ site and the *attR* site of prophages prophiT36-2, prophiT38-2, and prophiT48-2. Culture or supernatant sample input indicated as in panel B. Either primers 1 and 2, or 3 and 4 were used for PCR as shown in panel C.
(PDF)

S13 Fig Detection of additional phage virions by PCR. Figure format is as described for S12 Fig but showing PCR products for MabJ prophages (panel A) and MabL prophages (panel B).
(PDF)

S14 Fig Phage infection phenotypes. Examples of phage infection phenotypes are shown within different categories of the efficiency of plaquing (EOP). The names of the phages are shown at the left with their subcluster designations in parenthesis. Ten-fold serially diluted phage lysates were spotted onto bacterial lawns (indicated on the right), with the lowest concentrations at the right. Two examples are shown of the Killing Without Plaquing (KWP) phenotype, where individual plaques are not observed.
(PDF)

S15 Fig Phage susceptibilities of phiT45-1 lysogens. Results from screens of phiT45-1 lysogens and respective wild-type strains are shown in three panels. The bacterial strain is labeled below each plate; the phages spotted onto the bacterial overlays in ten-fold serial dilutions are listed down the left side, with subcluster designations in parenthesis.
(PDF)

S1 Raw images (PDF)
