# Supplementary material for: Unusual prophages in Mycobacterium abscessus genomes and strain variations in phage susceptibilities
Source: PLoS One. 2023 Feb 16;18(2):e0281769. doi: 10.1371/journal.pone.0281769 (PMC9934374; doi:10.1371/journal.pone.0281769)
Supplement: S1 Table — (DOCX) [file pone.0281769.s001.docx]

S1 Table. Genomic sequencing of *M. abscessus* strains.

| Strain^1^ | Length (bp)^2^ | Contigs^3^ | Coverage^4^ | Status^5^ | Accession #^6^ |
| --- | --- | --- | --- | --- | --- |
| T35 | 5248033 | 67 | 51 | WGS | SRR19357812 |
| T36 | 5075015 | 1 | 53 | Complete | SRR19357811 |
| T37 | 5337129 | 67 | 72 | WGS | SRR19357810 |
| T38 | 4990610 | 27 | 59 | WGS | JAQLTU000000000 |
| T44 | 4869593 | 30 | 93 | WGS | JAQLTT000000000 |
| T45 | 5371068 | 69 | 33 | WGS | SRR19357825 |
| T46 | 5335090 | 54 | 131 | WGS | JAQLTS000000000 |
| T48 | 4990171 | 34 | 149 | WGS | JAQLTR000000000 |
| T49 | 5092962 | 37 | 123 | WGS | JAQLTQ000000000 |
| T50 | 5295651 | 67 | 55 | WGS | SRR19357822 |
| T52 | 4826919 | 105 | 36 | WGS | JAQLTP000000000 |
| T56S | 5227337 | 72 | 133 | WGS | JAQLTO000000000 |
| T56R | 5227737 | 85 | 50 | WGS | JAQLTN000000000 |
| BWH-A | 4977099 | 43 | 70 | WGS | JAQLTM000000000 |
| BWH-B | 4995327 | 49 | 77 | WGS | SRR19357831 |
| BWH-C | 5088980 | 44 | 127 | WGS | JAQLTL000000000 |
| BWH-D | 5117974 | 15 | 209 | WGS | JAQLTG000000000 |
| CCUG50184-T | 5053525 | 24 | 150 | WGS | PRJNA281565 |
| CCUG48898-T | 4969787 | 25 | N/A | WGS | SRR315577 |

^1^Clinically isolated strains were given designations based on their location of origin; strains received from Taiwan begin with “T”; the suffixes “S” and “R” indicate smooth and rough colony morphologies, respectively; “BWH” denotes strains isolated from Brigham and Women’s Hospital, Boston, Massachusetts; type strains from the Culture Collection University of Gothenburgh archives are indicated with the prefix “CCUG”.

^2^For T36, the length of the complete genome is provided. For all others, the length shown is the sum of all contig lengths.

^3^The number of contigs greater than 100 bp generated following assembly with Unicycler v 0.4.8.

^4^WGS coverage.

^5^Whole genome shotgun sequencing performed with an Illumina MiSeq; complete genome sequencing performed with Illumina HiSeq and Oxford Nanopore.

^6^Accession numbers under which the strain sequences can be found. In cases where a Genome accession number is available, it has been provided. In other cases, the Sequence Read Archive (SRA) number is provided.
