# Supplementary material for: Unusual prophages in Mycobacterium abscessus genomes and strain variations in phage susceptibilities
Source: PLoS One. 2023 Feb 16;18(2):e0281769. doi: 10.1371/journal.pone.0281769 (PMC9934374; doi:10.1371/journal.pone.0281769)
Supplement: S2 Table — (PDF) [file pone.0281769.s002.pdf]

S2 Table. *attB* characteristics and coordinates in *M. abscessus* type strain ATCC19977

Cluster	Core Sequence	Int	attB	attB	ATCC19977 coordinates	Comments
**MabA1**	GGGTTCGAAACCCTCCGCGCCCACCAA	Int-Y	attB-20	tRNA-Val (Mab_t5029)	1739377	Shorter than other MabA1s
**MabB**	GTAATGAATAGGTCGGGGGTTCGATTCCCCCGGGCAGCTC<br>GTAATGAATAGGTCCGGGGTTGGCTTCCCCTGGATTGTCA<br>GTAATGAATAGGTCAGGGGTTCGATTCCCCTGGGTGGCTC	Int-Y	attB-2	tRNA-Thr (Mab_t5010)	490925	Flank MabN-like prophage region (T36-2a)<br>New mismatches
**MabC**	TACTCGTGAGTAAGAACT	Int-Y	attB-13	Mab_3947	3995698	
**MabG**	CGGGTTCAATTCCCGGCAGCTCCAC	Int-Y	attB-11	tmRNA	3513406	
**MabI**	GGGCT	Int-S	attB-17	Mab_3265c	3302860	
**MabJ**	AAGTCGTA	Int-S	attB-7	Mab_2445	2502029	
	AC		attB-21	Mab_1851	18493335	
**MabK**	CAGAAGGTTAGGGGTTCGAATCCCTTCGGGCGCACCA**T**<br>CAGAAGGTTGGGGGTTCGAATCCCTTCGGGCGCACCA**C**	Int-Y	attB-1	tRNA-Arg (Mab_t5006)	233517	
**MabL**	AGGGGTTCGAGTCCCCTTAGCTCCAC<br>AGGGGTTCGAGTCCCCTTAGCTCCAC**AATA**<br>AGGGGTTCGAGTCCCCTTAGCTCCAC**CATA**	Int-Y	attB-10	tRNA-Ala (Mab_5041c)	3491832	
