# Supplementary material for: Unusual prophages in Mycobacterium abscessus genomes and strain variations in phage susceptibilities
Source: PLoS One. 2023 Feb 16;18(2):e0281769. doi: 10.1371/journal.pone.0281769 (PMC9934374; doi:10.1371/journal.pone.0281769)
Supplement: S3 Table — (PDF) [file pone.0281769.s003.pdf]

S3 Table. Presence of *attP* and *attB* in *M. abscessus* cultures and culture supernatants

Strain	Prophage	Cluster	attP; culture		attP; s/n		attB culture		attB s/n	
			1	2	1	2	1	2	1	2
T36	prophiT36-1	MabL	+++	+++	+++	+++	+++	+++	++	++
	prophiT36-2	MabB	++	+++	-	+/-	++	+++	++	++
	prophiT36-2a	MabN	++	++	+	++	+++	+++	+	++
	prophiT36-2b	MabB	+++	+++	++	++	Same as 36-2 attR	Same as 36-2 attR	Same as 36-2 attR	Same as 36-2 attR
T37	prophiT37-1	MabK	+++	+++	+++	+++	-	+++	-	-
T38	prophiT38-1	MabL	+++	+++	+++	+++	+++	+++	++	+
	prophiT38-2	MabB	+	+++	-	-	+++	+++	++	++
	prophiT38-2a	MabN	+++	+++	++	++	+++	+++	++	++
	prophiT38-2b	MabB	+++	+++	++	++	Same as 38-2 attR	Same as 38-2 attR	Same as 38-2 attR	Same as 38-2 attR
T45	prophiT45-1	MabL	++	+++	++	++	*ND	ND	ND	ND
	prophiT45-2	MabJ	-	-	-	-	-	-	-	-
T46	prophiT46-1	MabG	+++	+++	+++	+++	+++	+++	++	++
	prophiT46-2	MabL	+++	+++	+++	+++	-	+++	-	++
	prophiT46-3	MabJ	+++	+++	+++	++	-	+++	-	++
T48	prophiT48-1	MabL	+++	+++	+++	+++	-	+++	+	++
	prophiT48-2	MabB	+	+++	-	-	+++	+++	-	++
	prophiT48-2a	MabN	+++	+++	++	+++	+++	+++	++	++
	prophiT48-2b	MabB	+++	+++	++	++	Same as 48-2 attR	Same as 48-2 attR	Same as 48-2 attR	Same as 48-2 attR
T49	prophiT49-1	MabL	+++	+++	+	++	-	+++	-	++
	prophiT49-2	MabI	+++	+++	+++	+++	-	+/-	-	-
	prophiT49-3	MabJ	+++	+++	+++	+++	+++	+++	++	++
T50	prophiT50-1	MabB	-	+++	+++	+++	+++	+++	++	++
BWH-A	prophiBWHA-1	MabJ	+++	+++	+++	+++	++	+++	-	++
BWH-B	prophiBWHB-1	MabJ	+++	-	+++	+++	+++	++	++	+
BWH-D	prophiBWHD-1	MabE1	+++	++	++	+/-	++	++	++	++
CCUG50184-T	prophi CCUG50184-T-1	MabL	-	-	++	++	+	+++	-	-
CCUG48898-T	prophi CCUG48898-T-1	MabC	-	++	+/-	+/-	++	+++	+	+
	prophi CCUG48898-T-2	MabA1	-	++	+++	+++	+	+++	-	+/-

*WGS contig positioning does not allow for designing primers that straddle *attB*
