# Supplementary material for: Unusual prophages in Mycobacterium abscessus genomes and strain variations in phage susceptibilities
Source: PLoS One. 2023 Feb 16;18(2):e0281769. doi: 10.1371/journal.pone.0281769 (PMC9934374; doi:10.1371/journal.pone.0281769)
Supplement: S4 Table — (PDF) [file pone.0281769.s004.pdf]

S4 Table. Primers used in this study

Primers	5' – 3' sequence	Product Length (bp)
phiBWHA-1 attP F1	CGTTGCCGTTCTACAGAGGT	463
phiBWHA-1 attP R1	CGCGGAGCTACAACAACTGA	
phiBWHA-1 attP F2	ACGTTGCCGTTCTACAGAGG	466
phiBWHA-1 attP R2	GACGCGGAGCTACAACAACT	
phiT36-1 attP F1	TATGAGAATCCGTACCGGCG	475
phiT36-1 attP R1	CACCGTCATCTTGGCCTCAT	
phiT36-1 attP F2	ATGAGAATCCGTACCGGCGA	470
phiT36-1 attP R2	GTCATCTTGGCCTCATTGCG	
phiT37-1 attP F1	AGCTATTTGTTACCAAACTGAGAGA	444
phiT37-1 attP R1	GTCTGCGATACCGCCTACAG	
phiT37-1 attP F2	AAGCTATTTGTTACCAAACTGAGAG	441
phiT37-1 attP R2	GCGATACCGCCTACAGATCG	
phiT38-1 attP F1	CCAGCATGTTCCAGGTCGAG	465
phiT38-1 attP R1	CTGATAGAGCTCGCATGCCC	
phiT38-1 attP F2	GCCGGACGGCTTCTATGAG	442
phiT38-1 attP R2	TGATAGAGCTCGCATGCCC	
phiT45-2 attP F1	TGCCTCGAAGGGGTCGATAG	451
phiT45-2 attP R1	CCGATCGTGATTCGGGAGTT	
phiT45-2 attP F2	GTGCCTCGAAGGGGTCGATA	426
phiT45-2 attP R2	CATGGAGTCGATCCACCAGG	
phiT46-1 attP F1	GGGTTTGGCAAGTCGGAGT	445
phiT46-1 attP R1	TTTCAGTATCTCCAGCGCCC	
phiT46-1 attP F2	GGGTTTGGCAAGTCGGAGTC	420
phiT46-1 attP R2	GGACCGAGGCGGTTCATTAG	
phiT46-2 attP F1	CCAGCATGTTCCAGGTCGAG	444
phiT46-2 attP R1	CACCATGTGCGTCAACTGC	
phiT46-2 attP F2	CCCAGCATGTTCCAGGTCG	446
phiT46-2 attP R2	GCACCATGTGCGTCAACTG	
phiT46-3 attP F1	TGCCTCGAAGGGGTCGATA	424
phiT46-3 attP R1	ATGGAGTCGATCCACCAGGA	
phiT46-3 attP F2	TGCCTCGAAGGGGTCGATAG	451
phiT46-3 attP R2	CCGATCGTGATTCGGGAGTT	
phiT48-1 attP F1	CCAGCATGTTCCAGGTCGAG	465
phiT48-1 attP R1	CTGATAGAGCTCGCATGCCC	
phiT48-1 attP F2	CCCAGCATGTTCCAGGTCG	472
phiT48-1 attP R2	GCTCAGCTGATAGAGCTCGC	
phiT49-1 attP F1	CCAGCATGTTCCAGGTCGAG	465
phiT49-1 attP R1	CTGATAGAGCTCGCATGCCC	
phiT49-1 attP F2	CCCAGCATGTTCCAGGTCG	472
phiT49-1 attP R2	GCTCAGCTGATAGAGCTCGC	
phiT49-2 attP F1	GCGTACGGTGTCCAGTGTTT	408
phiT49-2 attP R1	GGCTAGCTGACAGACGTGG	
phiT49-2 attP F2	GCGTACGGTGTCCAGTGTT	409
phiT49-2 attP R2	GGGCTAGCTGACAGACGTG	409
phiT49-3 attP F1	TAGATCCTCGAACGTCCCCA	400
phiT49-3 attP R1	TGGTTATCTGTGAGGTGCGG	
phiT49-3 attP F2	CTAGATCCTCGAACGTCCCC	400
phiT49-3 attP R2	GGTTATCTGTGAGGTGCGGT	
phiT50-1 attP F1	GTCTTGTCCATCGGATCGGG	456
phiT50-1 attP R1	ACGACGGATCAAGTACAGCG	
phiT50-1 attP F2	TACACGTACCCGAGTTTGCG	440
phiT50-1 attP R2	GCAGGACGACGGATCAAGTA	
phiBWHD-1 attP F1	TGCTGCACGGTAGAGACTTC	461
phiBWHD-1 attP R1	CGAATGGTCGAGAGCTTGGT	

phiBWHD-1 attP F2	TTTGCTGCACGGTAGAGACTT	453
phiBWHD-1 attP R2	AGAGCTTGGTCAAGCATGAGA	
phiCCUG48898T-1 attP F1	TCTTCAGCTTCAGATAACGAGCA	436
phiCCUG48898T-1 attP R1	GGTTCCGGCAGACACTCTTG	
phiCCUG48898T-1 attP F2	CAGCTTCAGATAACGAGCAAACT	440
phiCCUG48898T-1 attP R2	AACAACTGGGTTCCGGCAG	
phiCCUG48898T-2 attP F1	CCATCACTGTTGGCGTCGAG	463
phiCCUG48898T-2 attP R1	GAGCTCAACGGCGCGAT	
phiCCUG48898T-2 attP F2	CAGCCATGGCAACCTCCC	435
phiCCUG48898T-2 attP R2	CTCAACGGCGCGATCCGA	
phiCCUG50184T-1 attP F1	CAGCATGTTCCAGGTCGAGA	443
phiCCUG50184T-1 attP R1	CACCATGTGCGTCAACTGC	
phiCCUG50184T-1 attP F2	GAGGCCAGACGGCTTCTAC	427
phiCCUG50184T-1 attP R2	CGCACCATGTGCGTCAACT	
phiT45-1 attP/attR F1	GCCCATCGACTACGCCG	582
phiT45-1 attP/attR R1	CTGTACGTCGCCGATCACC	
phiT45-1 attP/attR F2	CAGCCCATCGACTACGCC	597
phiT45-1 attP/attR R2	CTATCACGCCCTGCTGTACG	
phiT36-2a attP F1	TCATGGTGGTATCCGATGCAG	572
phiT36-2a attP R1	TAGTTGTGCCGTGGACATCG	
phiT36-2a attP F2	AGTCGTAGGGCCCGTCAT	579
phiT36-2a attP R2	GCCGTGGACATCGCACTA	
phiT36-2b attP F1	CACGATCGCGGTCGCTTC	533
phiT36-2b attP R1	ACGACCTTGATGGCTGTTGA	
phiT36-2b attP F2	GCAGTCCCCGTCGTCCAC	550
phiT36-2b attP R2	TGACGACCTTGATGGCTGTT	
phiT36-2 attP F1	ACGATCGCGGTCGCTTC	547
phiT36-2 attP R1	CCGTGGACATCGCACTAGAT	
phiT36-2 attP F2	CAGGCAGTCCCCGTCGTC	567
phiT36-2 attP R2	GCCGTGGACATCGCACTAGA	
prophiT36-1 attR F1	GAACCCCTGACCCCCACAC	480
prophiT36-1 attR R1	CGATGGGGTTCGTGTCGT	
prophiT36-1 attR F2	TGGAGCTAAGGGGACTCGAA	472
prophiT36-1 attR R2	GGTGCTTGTCGTGCTCTCA	
prophiT37-1 attR F1	CCGTAACGCGTATTTGCACT	489
prophiT37-1 attR R1	GACCAGAGTCTCCTCGGTCA	489
prophiT37-1 attR F2	GATAGATGGTGCGCCCGAAG	459
prophiT37-1 attR R2	TCCTCGGTCACTTCATGTGG	
prophiT38-1 attR F1	AACCCCTGACCCCCACAC	477
prophiT38-1 attR R1	ATGGGGTTCGTGTCGTCG	
prophiT38-1 attR F2	GAACCCCTGACCCCCACA	479
prophiT38-1 attR R2	GATGGGGTTCGTGTCGTCG	
prophiT45-2 attR F1	CGCACGCCAAGTCGTATAGT	478
prophiT45-2 attR R1	TCACAGTTCCGCGAGATAGC	
prophiT45-2 attR F2	CGCACGCCAAGTCGTATAG	461
prophiT45-2 attR R2	AGCGGACACATTGAAGGCTA	
prophiT46-1 attR F1	TTATTGTGGAGCTGCCGGG	454
prophiT46-1 attR R1	ATCGGCGATAGCCCTCGATA	
prophiT46-1 attR F2	GGAGCTGCCGGGAATTGAA	495
prophiT46-1 attR R2	TGCGCATGTGATTACTCCCG	
prophiT46-2 attR F1	TTCGCATCGAGAAGGTCAGG	483
prophiT46-2 attR R1	GCACGATTCGCACTGGATAG	
prophiT46-2 attR F2	GAGAAGGTCAGGGGTTCGAT	482
prophiT46-2 attR R2	CAGCGACATGTCCTCCTCC	
prophiT46-3 attR F1	CGCACGCCAAGTCGTATAGT	497
prophiT46-3 attR R1	TTGGCGGATGCTGACTGAAT	
prophiT46-3 attR F2	GCACGCCAAGTCGTATAGTAAC	467
prophiT46-3 attR R2	GCGAGATAGCGGACACATTG	
prophiT48-1 attR F1	AGGTCGTCTGACCTGGGATT	490

prophiT48-1 attR R1	CGACCCTGAAGCGGATCTAT	
prophiT48-1 attR F2	TGTGGAGCTAAGGGGACTCG	456
prophiT48-1 attR R2	GGATCTATCGGCTGGTGTACG	
prophiT49-1 attR F1	GGGATCGGAGCAGGTTTGTA	475
prophiT49-1 attR R1	ATTTCTCACTGCGTTTCCGC	
prophiT49-1 attR F2	ATCGGAGCAGGTTTGTACGC	464
prophiT49-1 attR R2	CTGCGTTTCCGCAGGTCATA	
prophiT49-2 attR F1	TTCACGAGAACAAGGGGCTG	417
prophiT49-2 attR R1	GCAATGTTGCTTCTACCTGCG	
prophiT49-2 attR F2	CGAGAACAAGGGGCTGTATGT	415
prophiT49-2 attR R2	ATGCAATGTTGCTTCTACCTGC	
prophiT49-3 attR F1	CAAAGACGTAGTTGACGTTGGT	461
prophiT49-3 attR R1	GCTTCACATGAGTAGGCGTC	
prophiT49-3 attR F2	TGCGGATAGTGCTGCGAAA	477
prophiT49-3 attR R2	ACCGAGGTGGGGCTTTAGT	
prophiT50-1 attR F1	AGGGGTTCGATTCCCCTGG	473
prophiT50-1 attR R1	ACATGTTCTGCGGCGAACTC	
prophiT50-1 attR F2	CTGGGTGGCTCCATCCCTTC	460
prophiT50-1 attR R2	GGACATGTTCTGCGGCGA	
prophiBWHA-1 attR F1	GGCGTGATCACCTACGACTT	482
prophiBWHA-1 attR R1	TTTCTTACGGGACGCCTTGG	
prophiBWHA-1 attR F2	CGACGGCGTGATCACCTAC	485
prophiBWHA-1 attR R2	TTCTTACGGGACGCCTTGG	
prophiBWHB-1 attR F1	CGACGGCGTGATCACCTAC	485
prophiBWHB-1 attR R1	TTCTTACGGGACGCCTTGG	
prophiBWHB-1 attR F2	GGCGTGATCACCTACGACTT	482
prophiBWHB-1 attR R2	TTTCTTACGGGACGCCTTGG	
prophiBWHD-1 attR F1	TGTAGCTCAATTGGCAGAGCA	478
prophiBWHD-1 attR R1	GGCGAAGACCTCCACCAGAC	
prophiBWHD-1 attR F2	GGCAGAGCATCCGACTGTTA	465
prophiBWHD-1 attR R2	GCGAAGACCTCCACCAGAC	
prophi50184T-1 attR F1	CCACTGGTGGTCGCCG	521
prophi50184T-1 attR R1	CCTGACCCCCACACTGC	
prophi50184T-1 attR F2	CGGGCTGGTGGTGTGTG	509
prophi50184T-1 attR R2	CCCCTGACCCCCACACTG	
prophi48898T-1 attR F1	GCTTGCCGAACGAGTTCTTAC	465
prophi48898T-1 attR R1	CATACGGCGCTGAATCTTCG	
prophi48898T-1 attR F2	GCCGAACGAGTTCTTACTCAC	463
prophi48898T-1 attR R2	GCCATACGGCGCTGAATCT	
prophi48898T-2 attR F1	GTTTTACACACCAGCGGTCG	483
prophi48898T-2 attR R1	CCCAACGCGACACATCCT	
prophi48898T-2 attR F2	TCTGGTTTTACACACCAGCGG	490
prophi48898T-2 attR R2	GGACCCAACGCGACACAT	
