# Supplementary figures and images for: Unusual prophages in Mycobacterium abscessus genomes and strain variations in phage susceptibilities
Source: PLoS One. 2023 Feb 16;18(2):e0281769. doi: 10.1371/journal.pone.0281769 (PMC9934374; doi:10.1371/journal.pone.0281769)

prophiCCUG48898T-2 (MabA1)

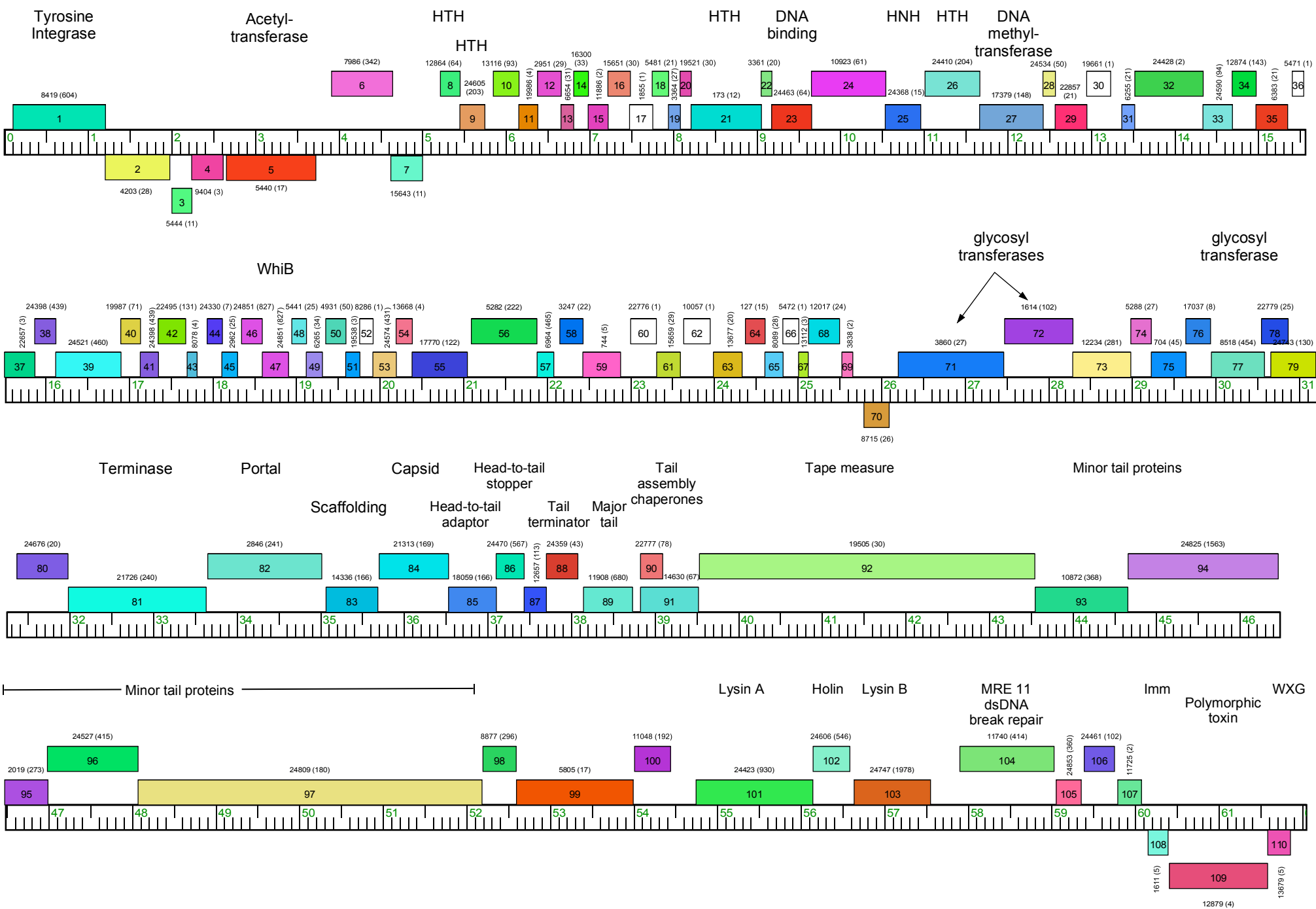

Figure S1

Supplement: S1 Fig — The horizontal ruler represents the nucleotide sequence of the phage genome, with each bar indicating 1kb. Predicted genes are shown as colored boxes above and below the ruler, indicating rightward and leftward transcription, respectively. Genes are colored according to their phamily assignment using Phamerator [41] and database Actino_Mab_4036. Phamily designations with the number of members are shown in parentheses above each gene. Genes with no close relatives in this data set (orphams), are shown as white boxes. Predicted functions are shown above the genes. (PDF) [file pone.0281769.s005.pdf]

prophiT50-1 (MabB)

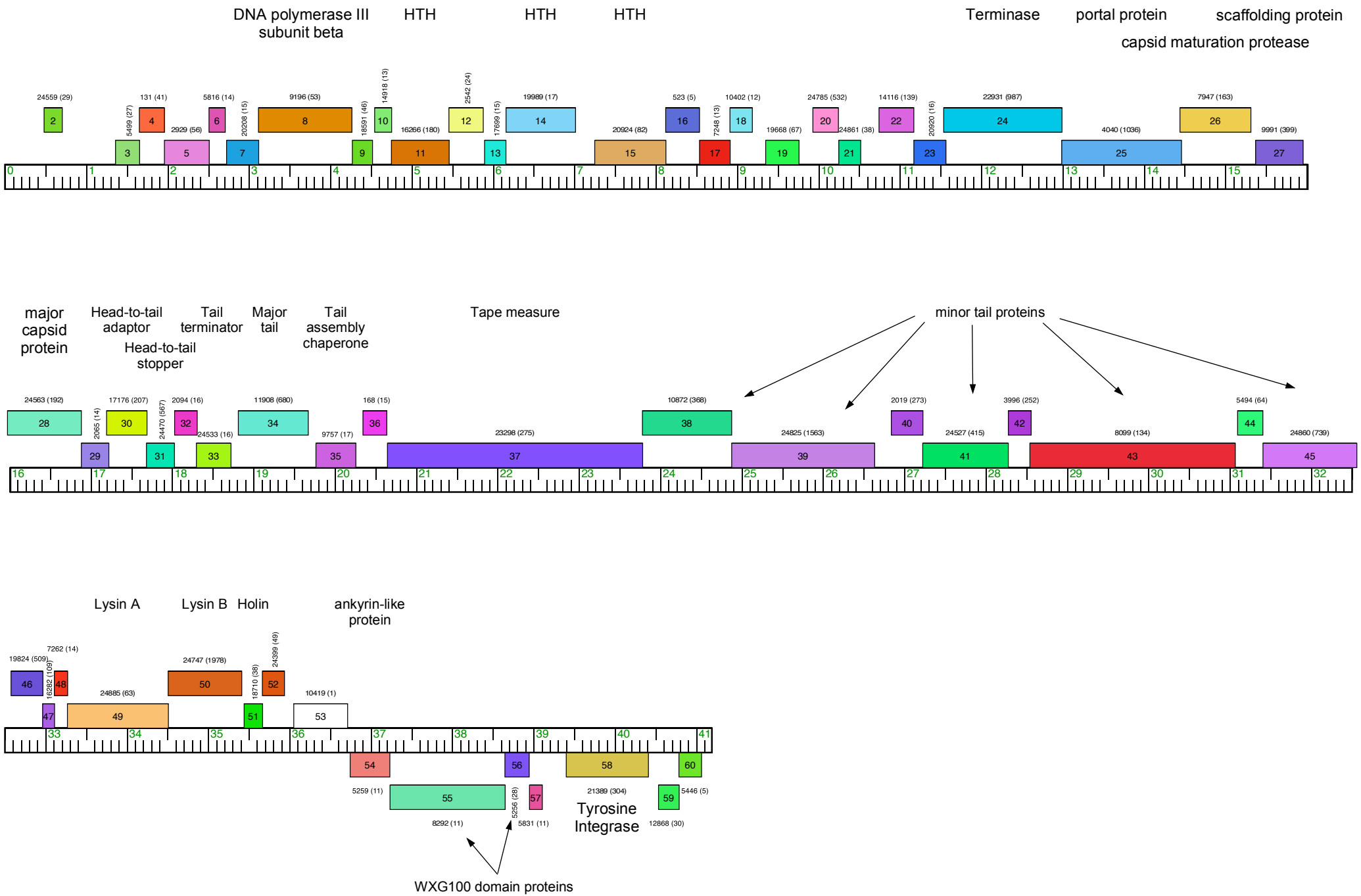

Figure S2

Supplement: S2 Fig — See S1 Fig for details. (PDF) [file pone.0281769.s006.pdf]

prophiCCUG48898T-1 (MabC)

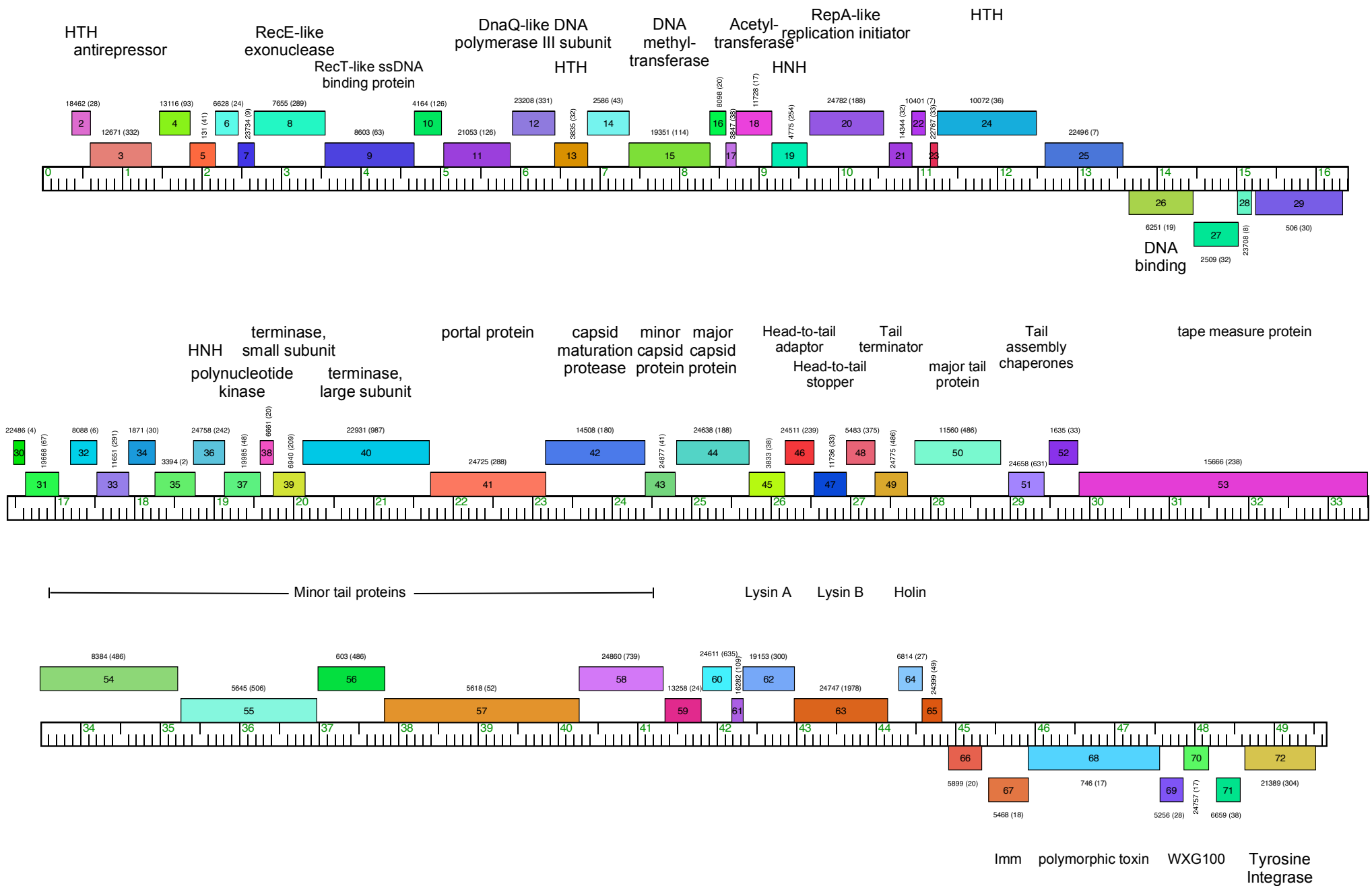

Figure S3

Supplement: S3 Fig — See S1 Fig for details. (PDF) [file pone.0281769.s007.pdf]

prophiT46-1 (MabG)

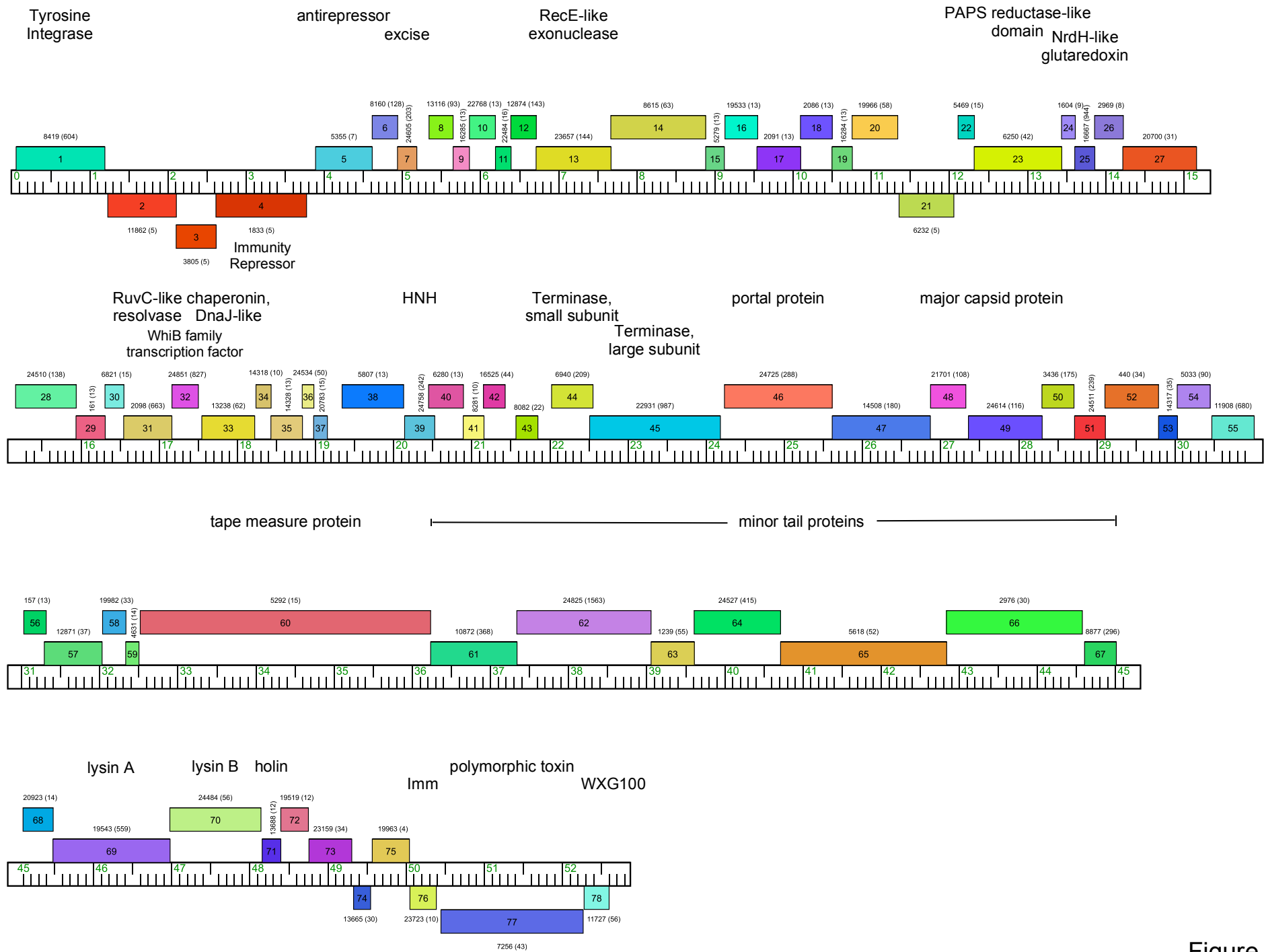

Figure S4

Supplement: S4 Fig — See S1 Fig for details. (PDF) [file pone.0281769.s008.pdf]

prophiT49-2 (MabI)

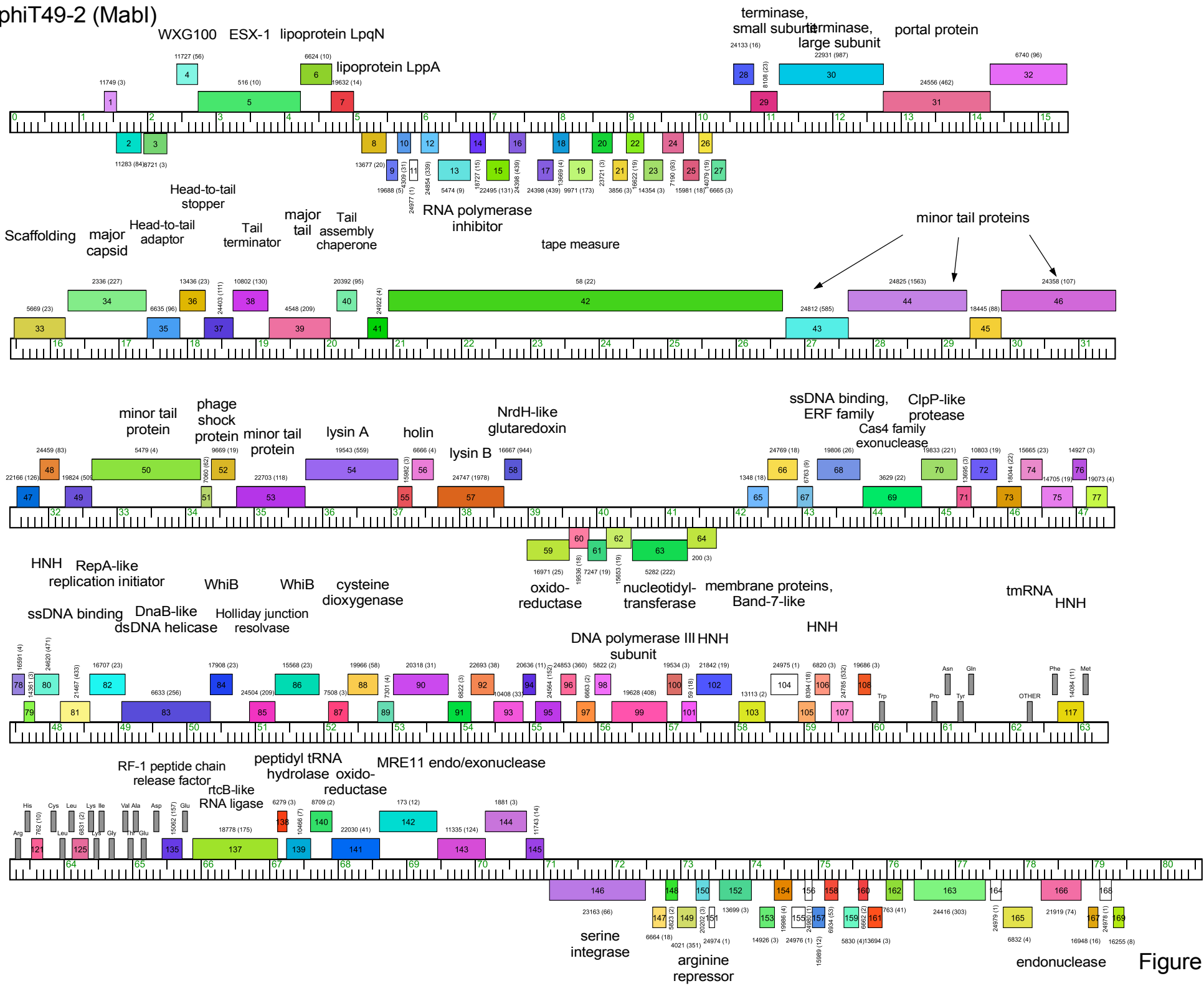

Figure S5

Supplement: S5 Fig — See S1 Fig for details. (PDF) [file pone.0281769.s009.pdf]

prophiT46-3 (MabJ)

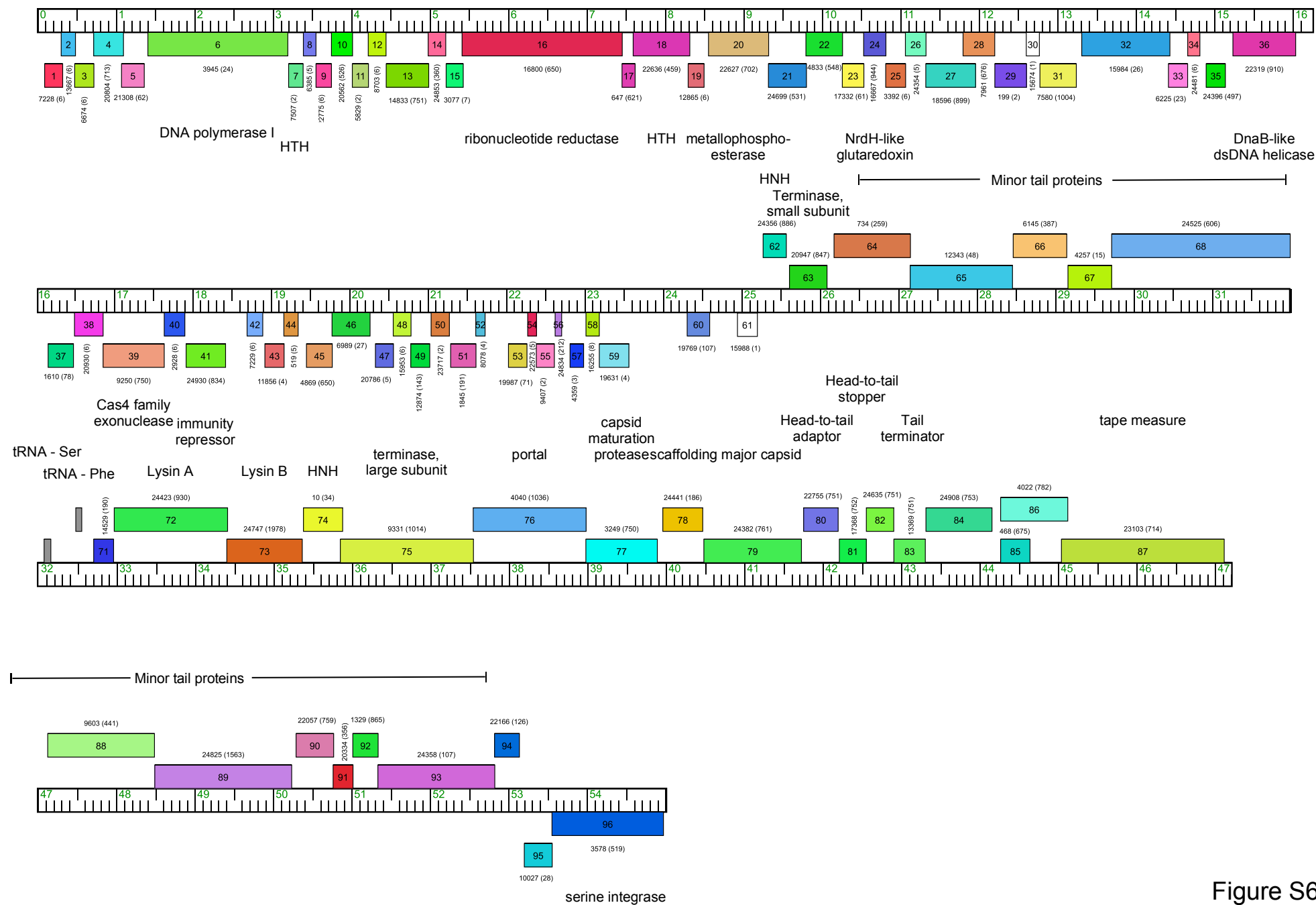

Figure S6

Supplement: S6 Fig — See S1 Fig for details. (PDF) [file pone.0281769.s010.pdf]

prophiBWHA-1 (MabJ)

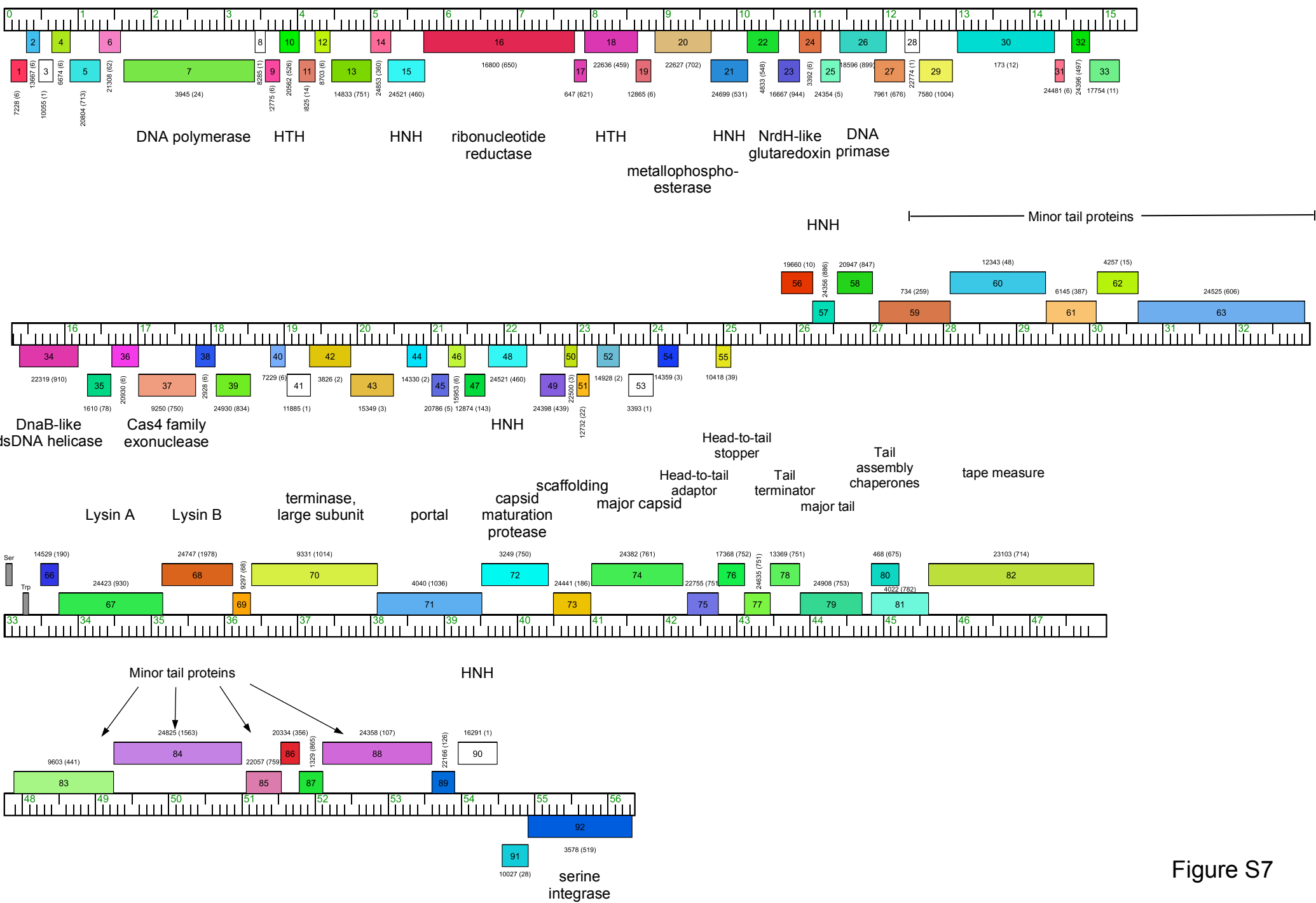

Figure S7

Supplement: S7 Fig — See S1 Fig for details. (PDF) [file pone.0281769.s011.pdf]

## prophiT37-1 (MabK)

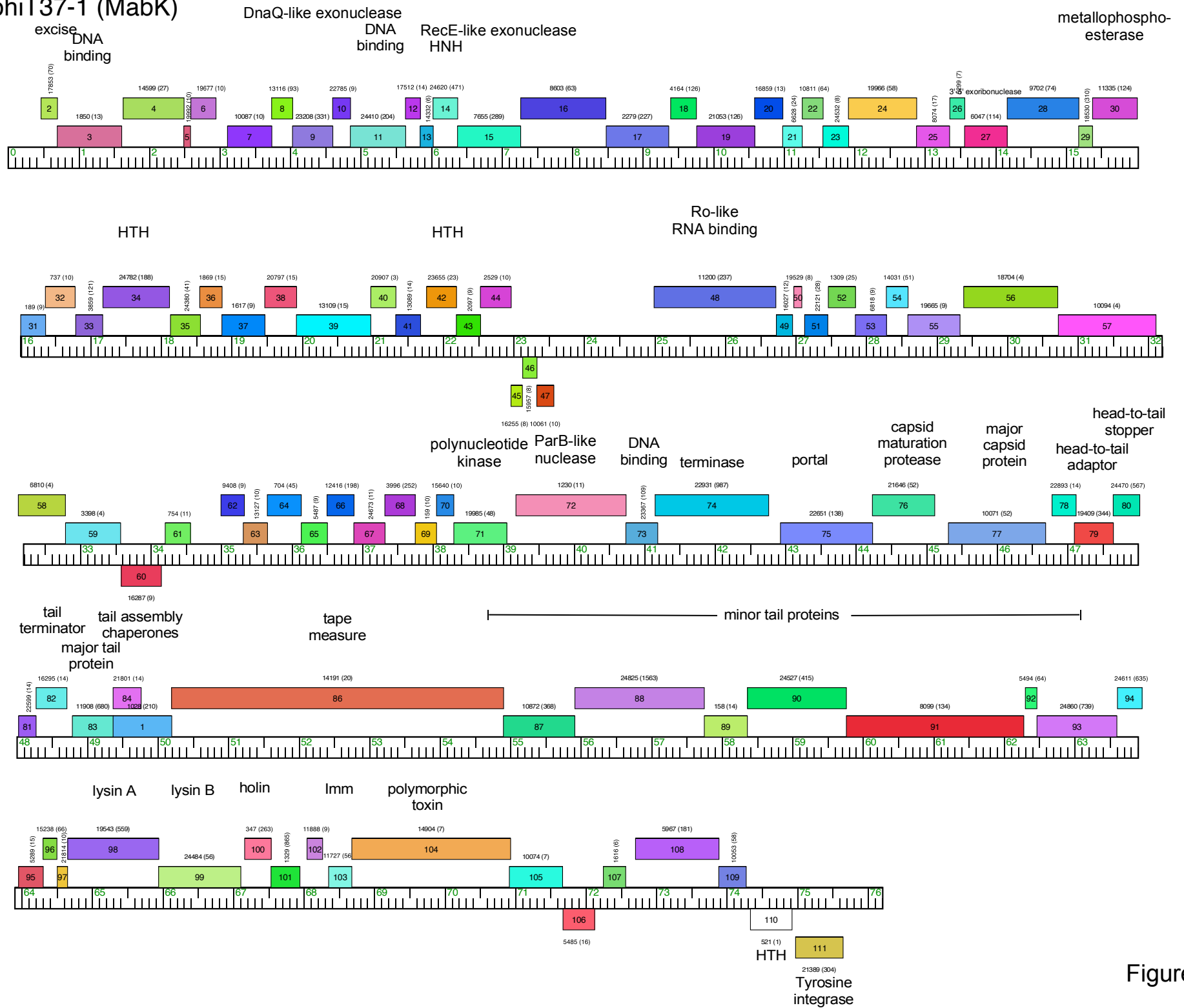

Figure S8

Supplement: S8 Fig — See S1 Fig for details. (PDF) [file pone.0281769.s012.pdf]

prophiT46-2 (MabL)

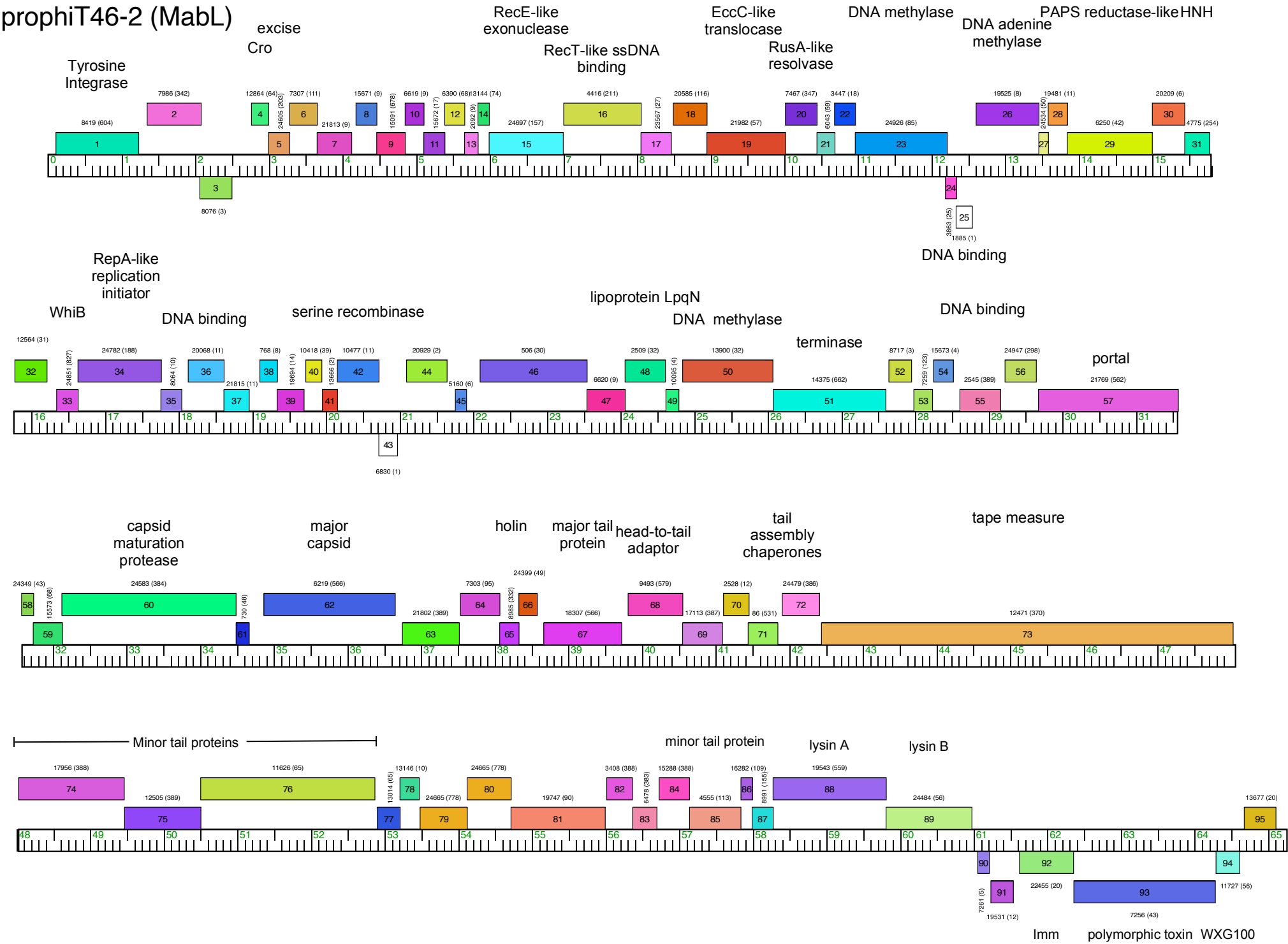

Figure S9

Supplement: S9 Fig — See S1 Fig for details. (PDF) [file pone.0281769.s013.pdf]

prophiT49-1 (MabL)

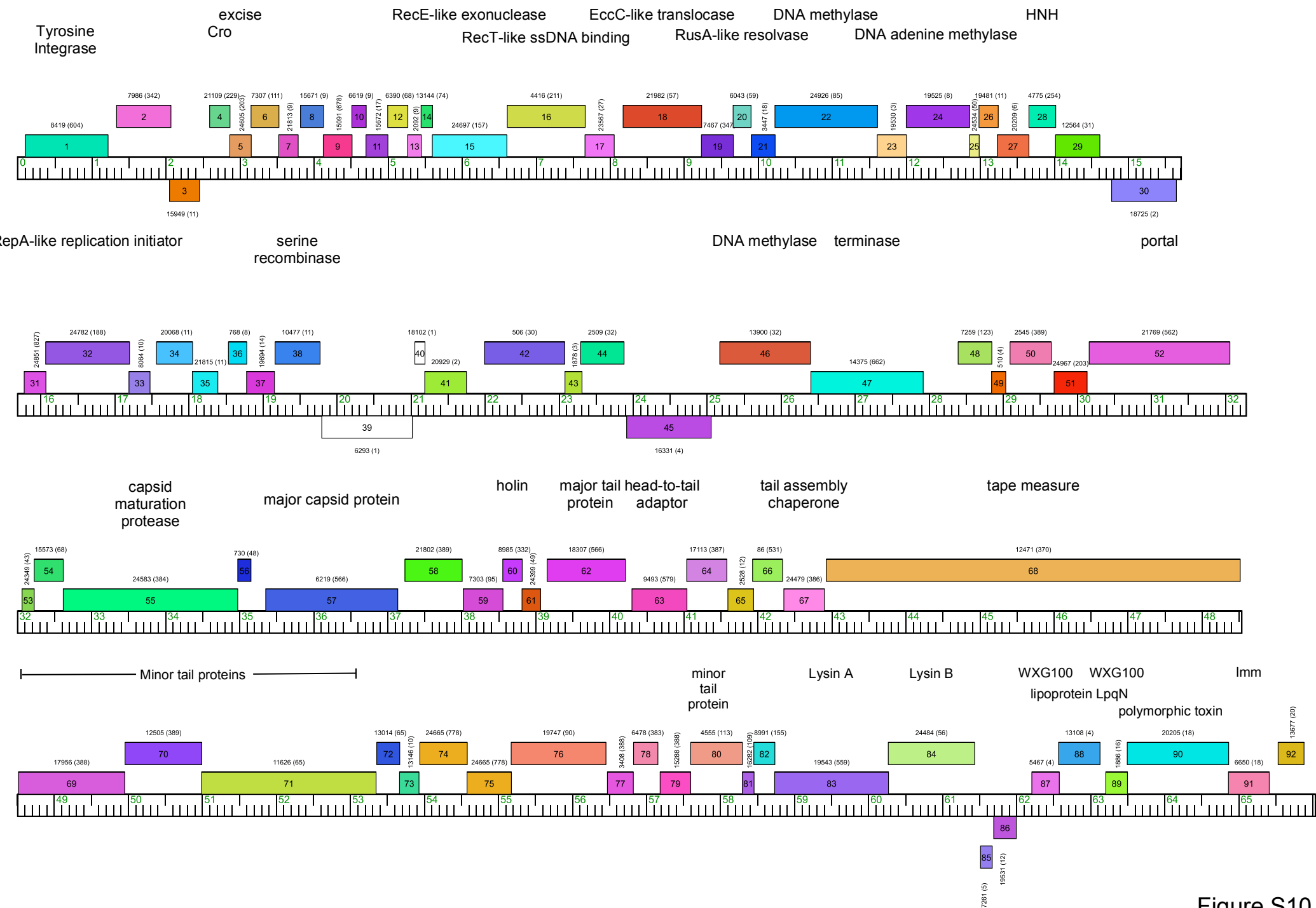

Figure S10

Supplement: S10 Fig — See S1 Fig for details. (PDF) [file pone.0281769.s014.pdf]

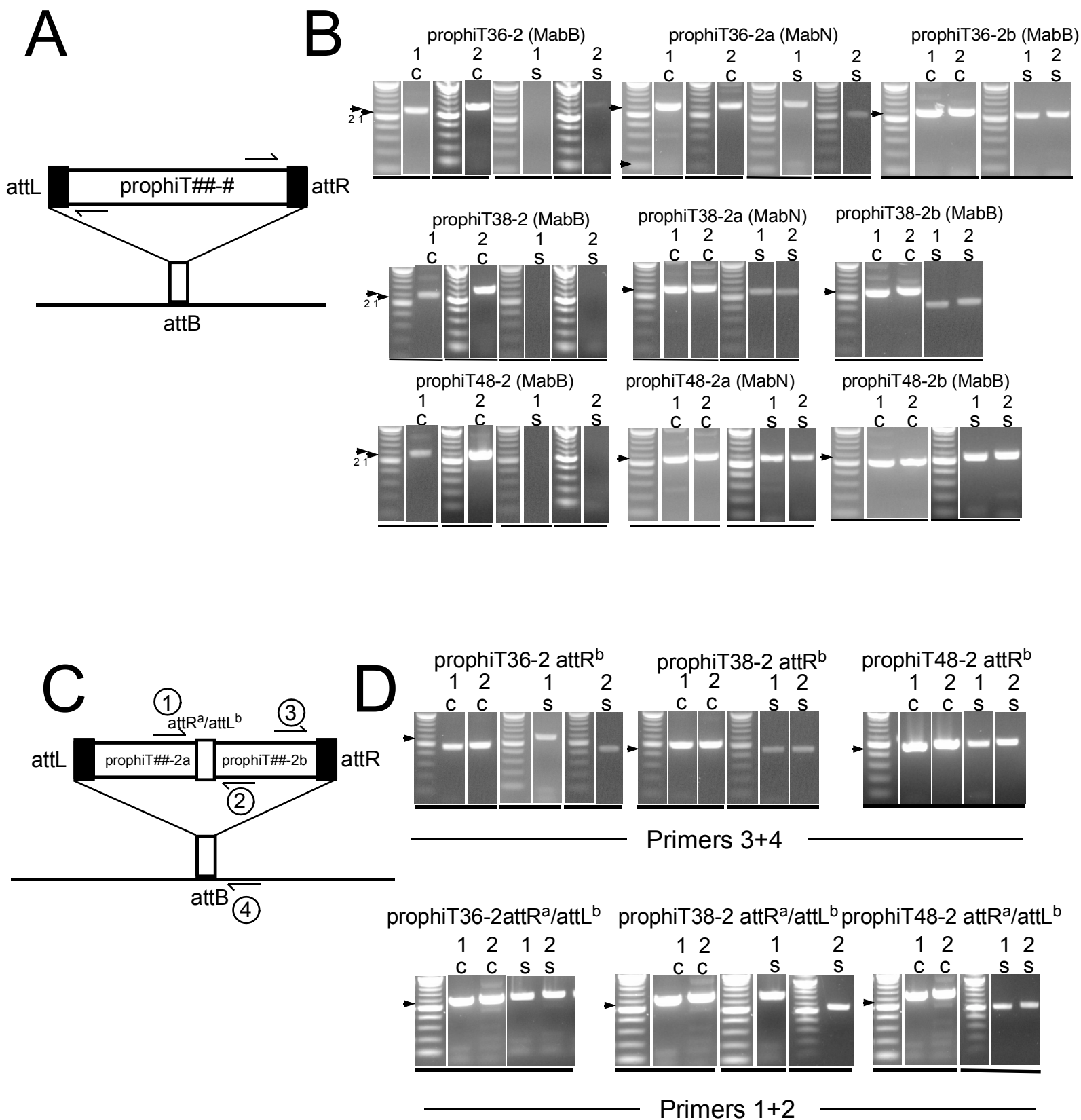

Figure S12

Supplement: S12 Fig — A. Schematic of the primer annealing locations denoted with right and left facing half-headed arrows for the forward and reverse primers respectively. The line represents the host chromosome, with attB indicated. The rectangle depicts the integrated prophage flanked by attL and attR. B. PCR products using primers flanking the predicted attP from the identical prophages prophiT36-2, prophiT38-2 and prophiT48-2. For many prophages, two primers sets were used, designated 1 (primer set 1) and 2 (primer set 2) above the lanes. The input sample for the PCR was either from a bacterial culture (C) or a culture supernatant (S) as indicated. C. Schematic of the primer annealing locations, denoted with half-headed arrows as in panel A, for the 2a-2b attRa/attLb site and the attR site of prophages prophiT36-2, prophiT38-2, and prophiT48-2. D. PCR products using primers amplifying 2a-2b attRa/attLb site and the attR site of prophages prophiT36-2, prophiT38-2, and prophiT48-2. Culture or supernatant sample input indicated as in panel B. Either primers 1 and 2, or 3 and 4 were used for PCR as shown in panel C. (PDF) [file pone.0281769.s016.pdf]

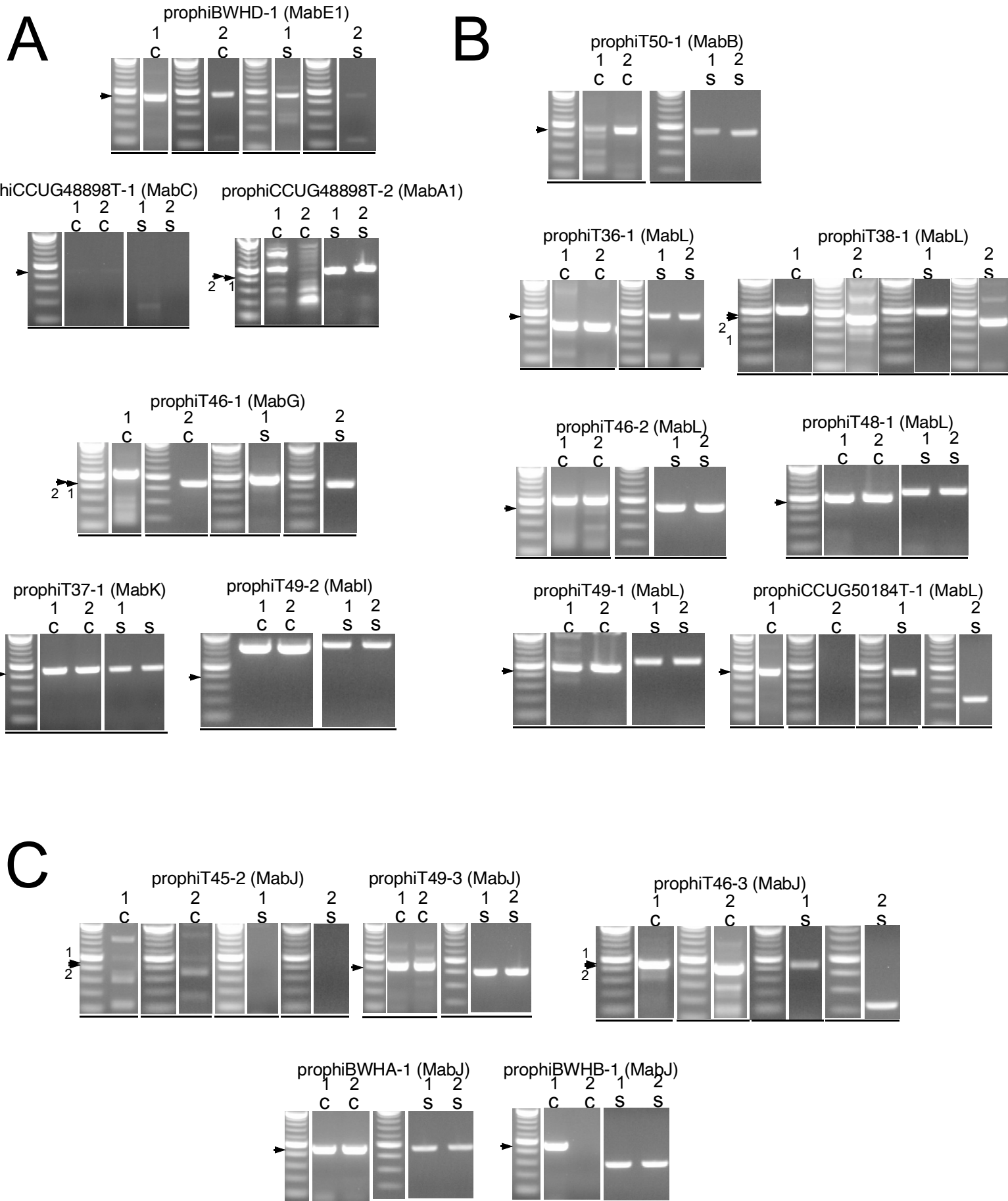

Figure S13

Supplement: S13 Fig — Figure format is as described for S12 Fig but showing PCR products for MabJ prophages (panel A) and MabL prophages (panel B). (PDF) [file pone.0281769.s017.pdf]

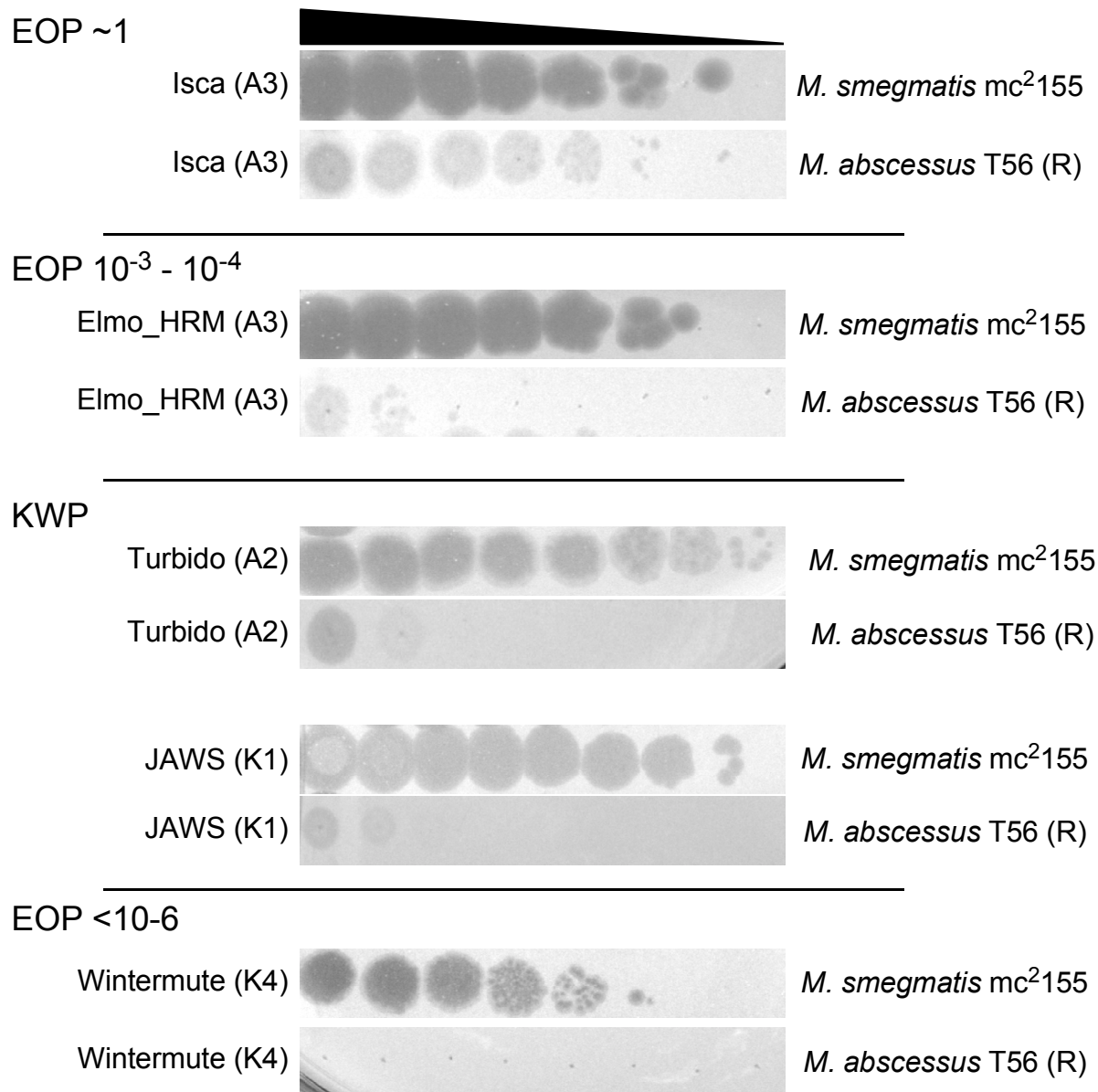

Figure S14

Supplement: S14 Fig — Examples of phage infection phenotypes are shown within different categories of the efficiency of plaquing (EOP). The names of the phages are shown at the left with their subcluster designations in parenthesis. Ten-fold serially diluted phage lysates were spotted onto bacterial lawns (indicated on the right), with the lowest concentrations at the right. Two examples are shown of the Killing Without Plaquing (KWP) phenotype, where individual plaques are not observed. (PDF) [file pone.0281769.s018.pdf]

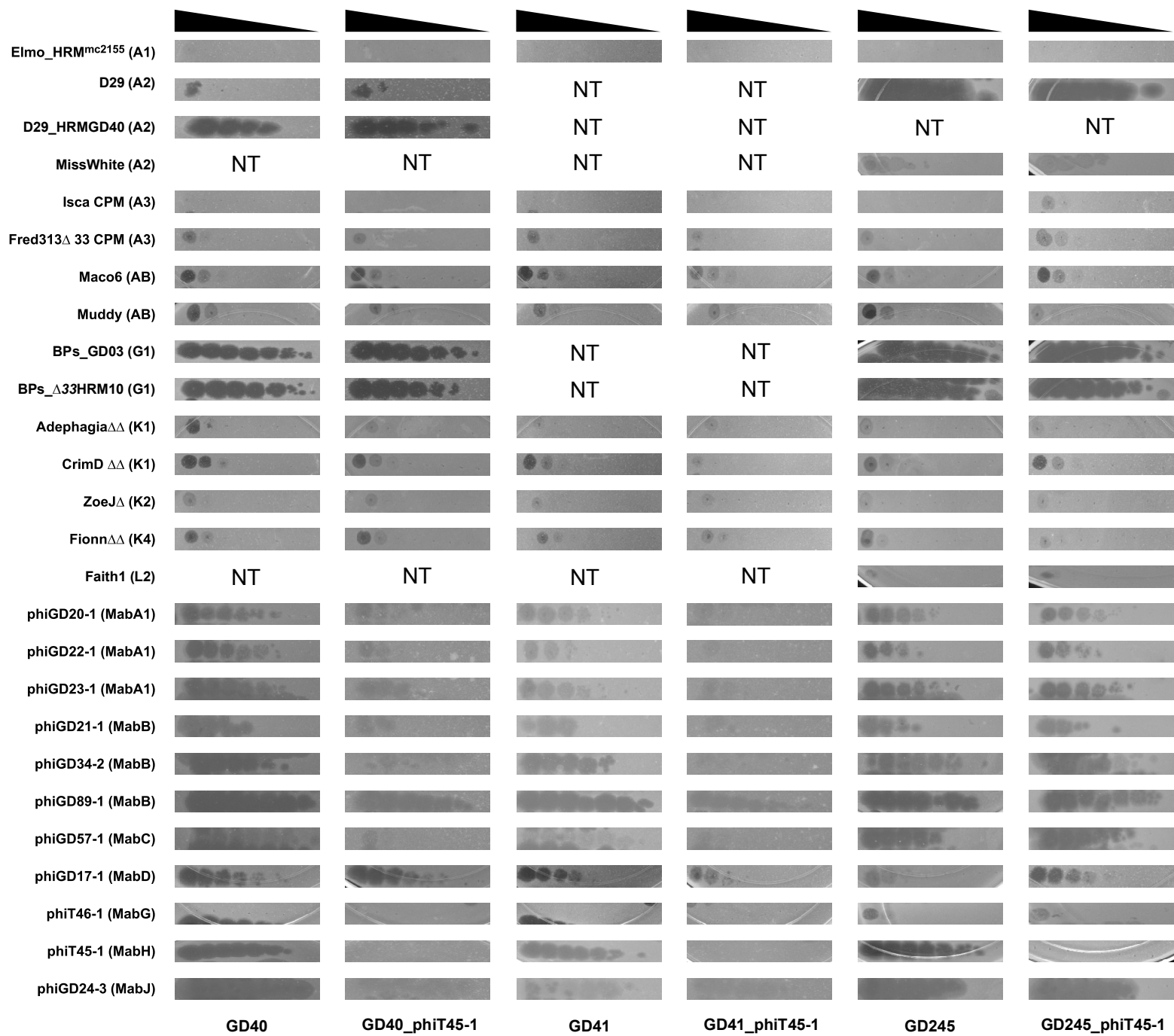

Figure S15

Supplement: S15 Fig — Results from screens of phiT45-1 lysogens and respective wild-type strains are shown in three panels. The bacterial strain is labeled below each plate; the phages spotted onto the bacterial overlays in ten-fold serial dilutions are listed down the left side, with subcluster designations in parenthesis. (PDF) [file pone.0281769.s019.pdf]
