# Supplementary material for: Unusual prophages in Mycobacterium abscessus genomes and strain variations in phage susceptibilities
Source: PLoS One. 2023 Feb 16;18(2):e0281769. doi: 10.1371/journal.pone.0281769 (PMC9934374; doi:10.1371/journal.pone.0281769)
Supplement: S11 Fig — Panels A-F show each strand of DNA sequence and the three translated reading frames that are transcribed leftwards. The 40 bp att common core sequence in each is shown in bold type. A. The sequence at the extreme left boundary of the prophiT36-2 prophage at attL (see Fig 3). The tRNAthr gene is shown in aqua colored shading. The 3’ end of the repressor encoded by prophiT36-2a is shaded in purple. B. The sequence at the center of the prophT36-2 prophage that defines the boundary between prophiT36-2a and prophiT36-2b; it is proposed that the common core corresponds to attR of prophiT36-2a and attL of prophiT36-2b, designated attR/attL. The 3’ end of the repressor gene encoded by phrphiT36-2b is shaded in red. The predicted 3’ remnant of the phage-encoded form of the prophiT36-2a repressor is shaded in green. C. The sequence at the extreme right boundary of the prophiT36-2 prophage at attR (see Fig 3). The predicted at the extreme left boundary of the prophiT36-2 prophage at attL (see Fig 3) 3’ remnant of the of the phage-encoded form of the prophiT36-2b repressor is shaded in yellow. D. The predicted attP of phage phiT36-2a reconstructed from the sequences in panels A and B. Note that the 3’ remnant of the phage-encoded repressor contributed from attR/attL (green) is in-frame with the 5’ part of the repressor contributed by attL (purple). E. The predicted attP of phage phiT36-2b reconstructed from the sequences in panels B and C. Note that the 3’ remnant of the phage-encoded repressor contributed from attR (yellow) is in-frame with the 5’ part of the repressor contributed by attR/attL (red). F. Sequence of a potential attP that could be formed by site-specific recombination between the outside boundaries, attL and attR. In this case, the putative 3’ remnant of the repressor gene (yellow) would be fused out of frame with the rest of the repressor (purple), and thus these attL and attR sites are not derived from the same phage. (PDF) [file pone.0281769.s015.pdf]

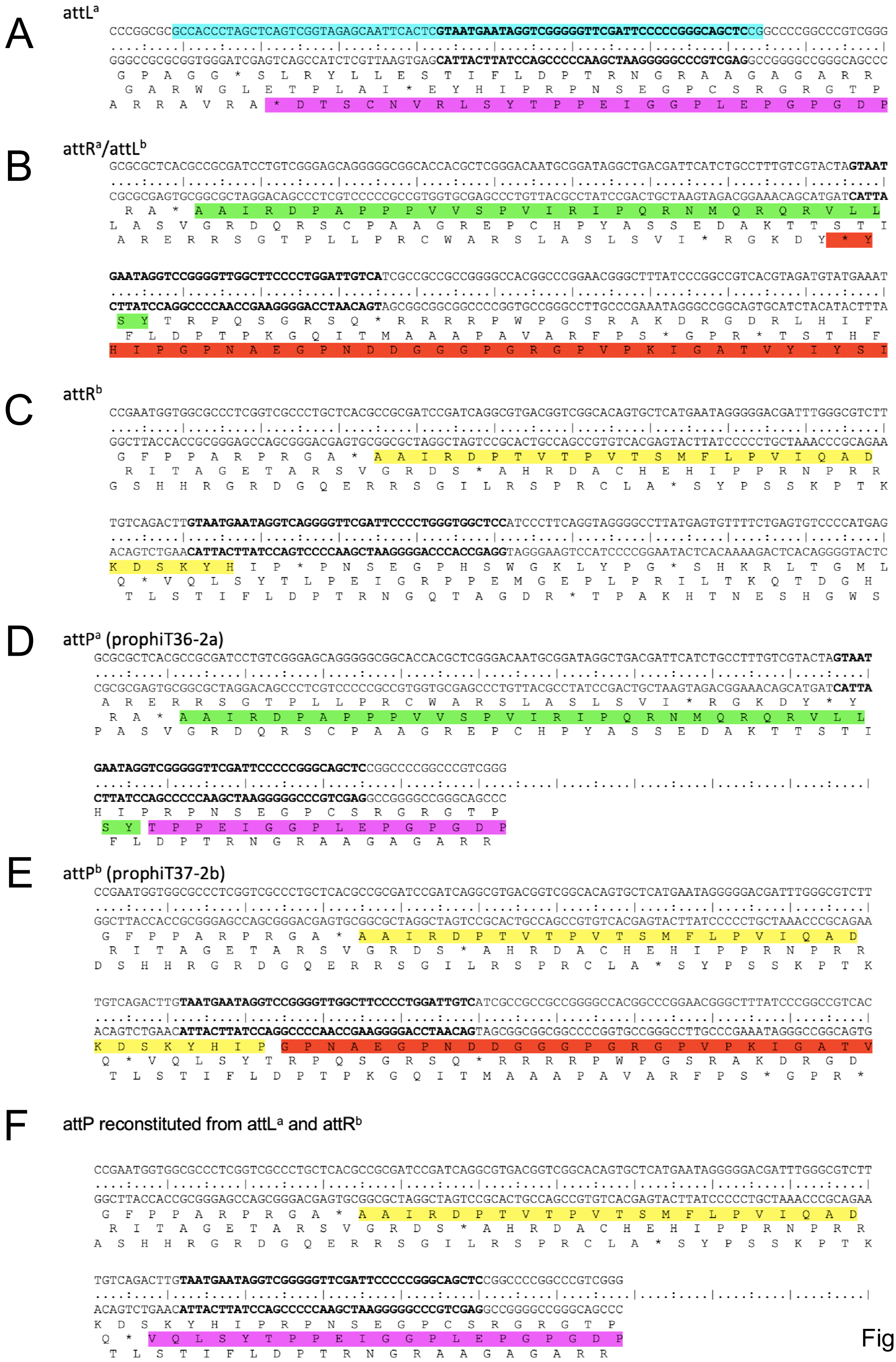
A
attL[a]
B
attR[a]/attL[b]
C
attR[b]
D
attP[a] (prophiT36-2a)
E
attP[b] (prophiT37-2b)
F
attP reconstituted from attL[a] and attR[b]

Figure S11
