# Supplementary material for: Unusual prophages in Mycobacterium abscessus genomes and strain variations in phage susceptibilities
Source: PLoS One. 2023 Feb 16;18(2):e0281769. doi: 10.1371/journal.pone.0281769 (PMC9934374; doi:10.1371/journal.pone.0281769)
Supplement: S1 Raw images — (PDF) [file pone.0281769.s020.pdf]

Figure 9 Group 1 phages

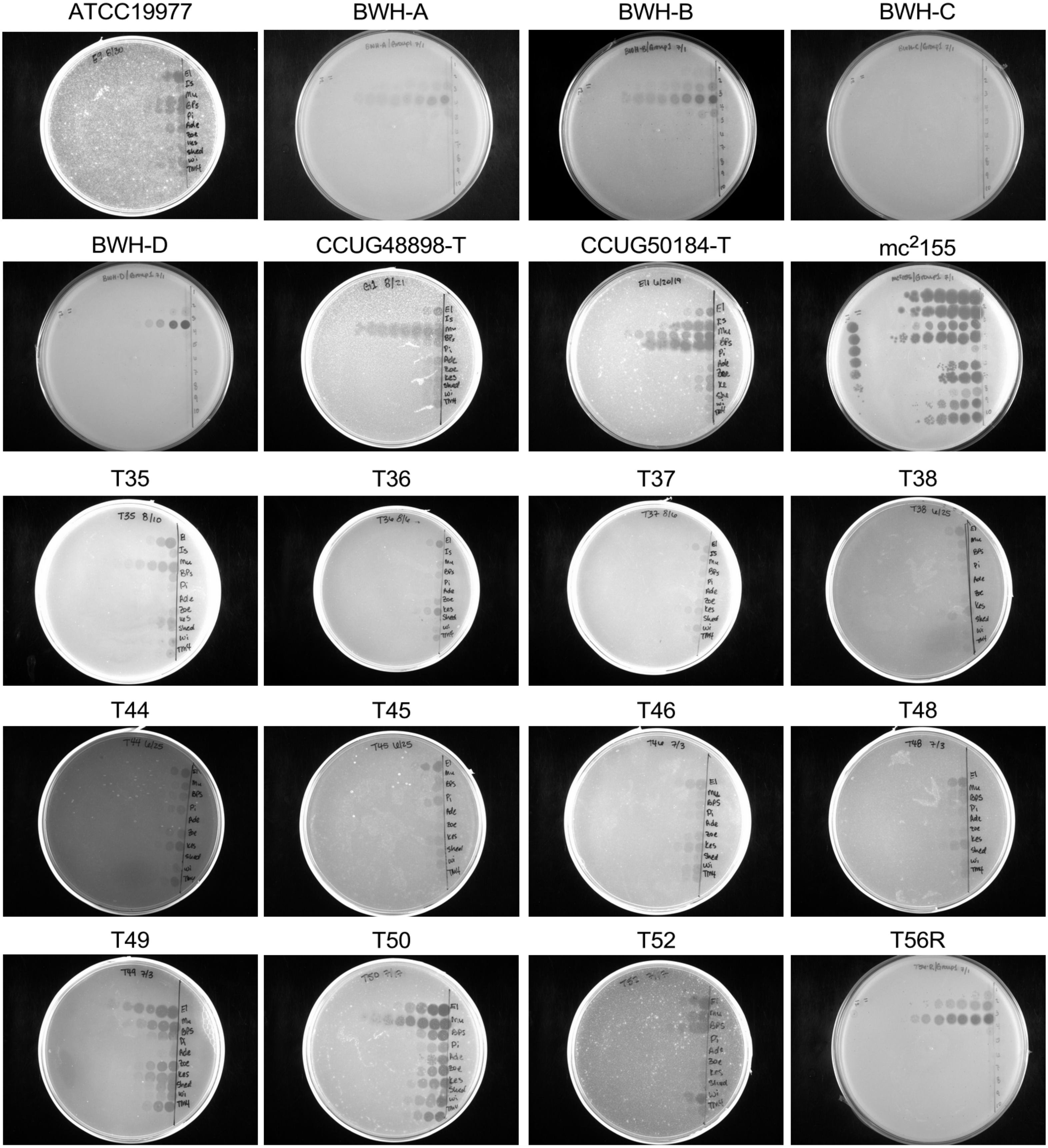

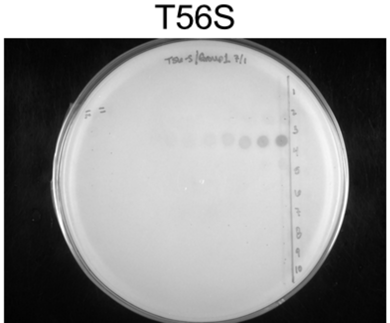

Group I Bacteriophages (cluster)
1. Elmo HRM (A3)
2. Isca (A3)
3. Muddy (AB)
4. BPs_HRM10_HTHΔ33 (G1)
5. Pinnie (G3)
6. Adephagia ΔΔ (K1)
7. ZoeJΔ45 (K2)
8. Keshu (K3)
9. ShedlockHolmes (K3)
10. Wintermute (K4)
11. TM4Δ (K2)
12. data not included

Figure 9 Group 2 phages

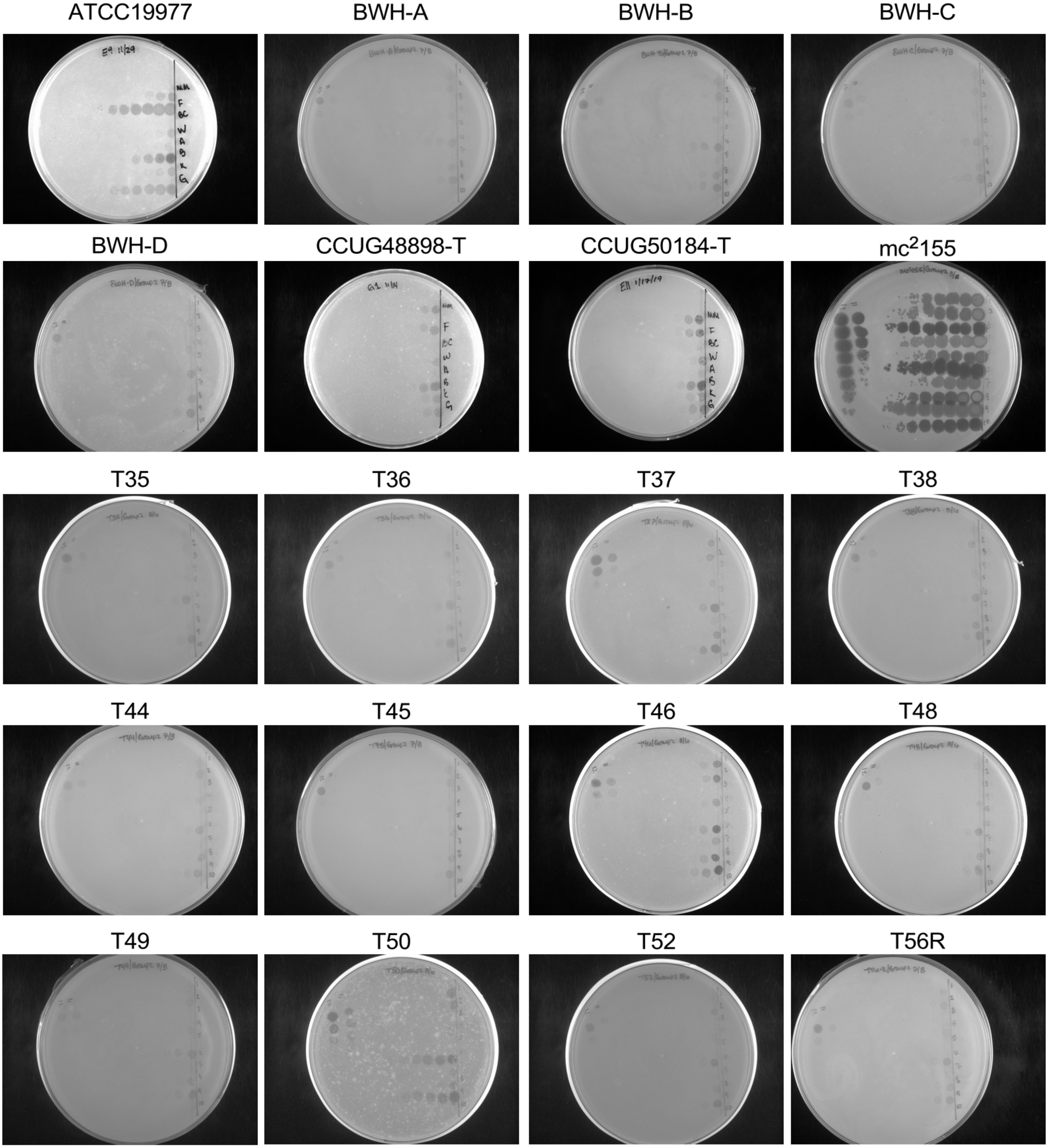

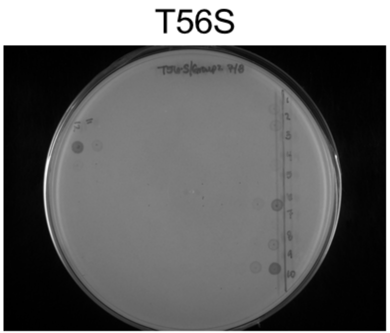


```
Group II Bacteriophages (cluster)
1. MkaliMitinis3 (L2)
2. Faith1 (L2)
3. BigCheese (L2)
4. Wilder (L2)
5. Archie (L2)
6. Breezona (L2)
7. Kahlid (L2)
8. Gardann (L2)
9. JAWS (L2)
10. Whirlwind (L2)
11. Angelica (L2)
12. Turbido (L2)
```

Figure 9 Group 3 phages

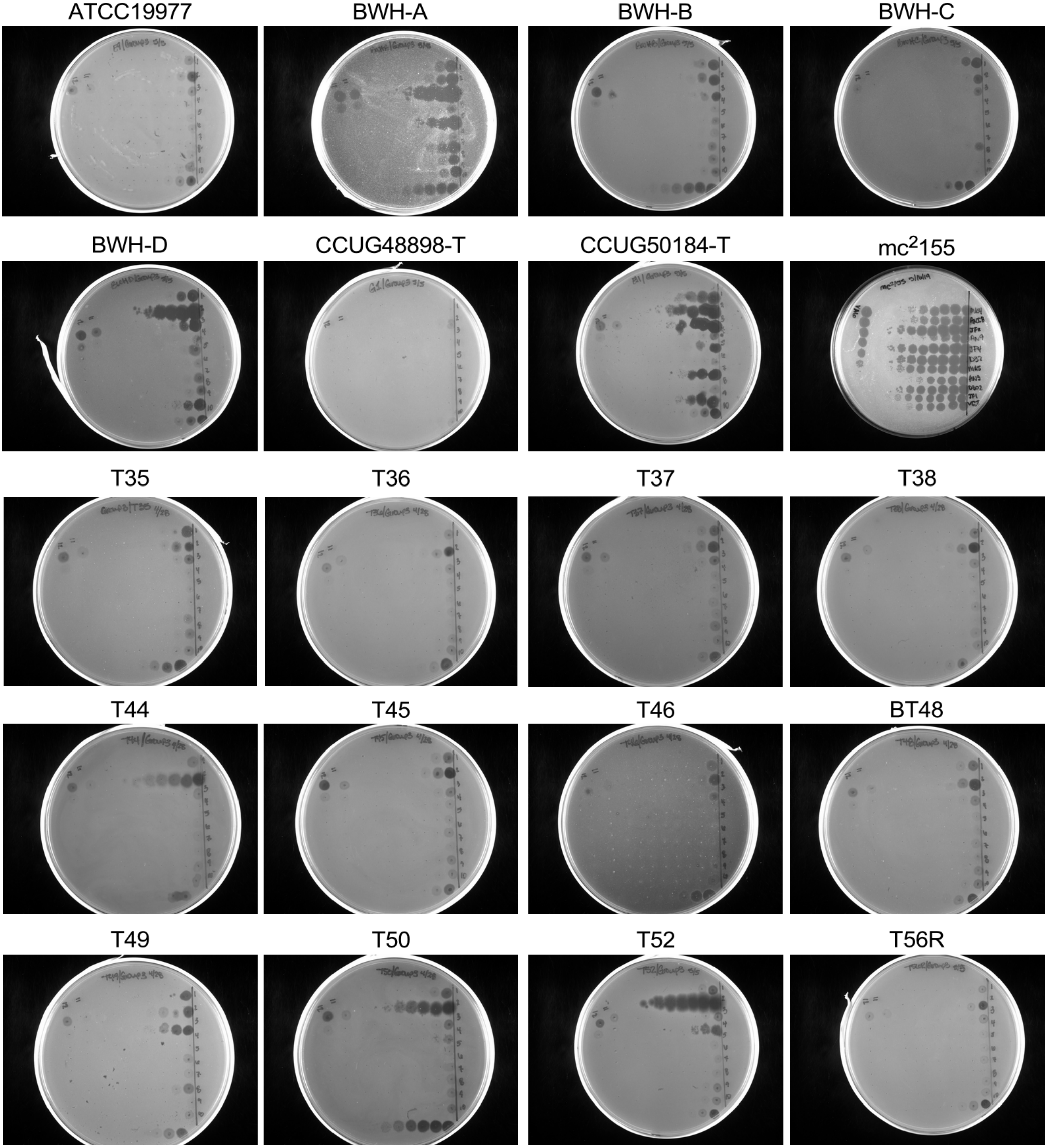

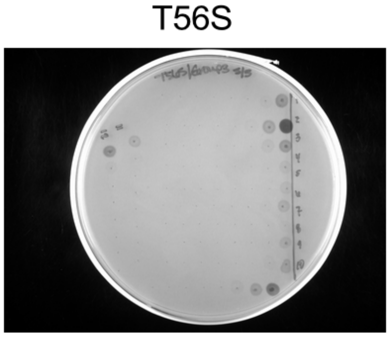

Group III Bacteriophages (cluster)
1. MK4 (A3)
2. ANI8 (A2)
3. JF2 (A3)
4. AN9 (A2)
5. JF4 (A3)
6. D32 (A2)
7. MA5 (A3)
8. AN3 (A2)
9. D302 (Uncl.)
10. JF1 (K4)
11. VC3 (A2)
12. VA6 (A2)

Figure 9 Group 4 phages

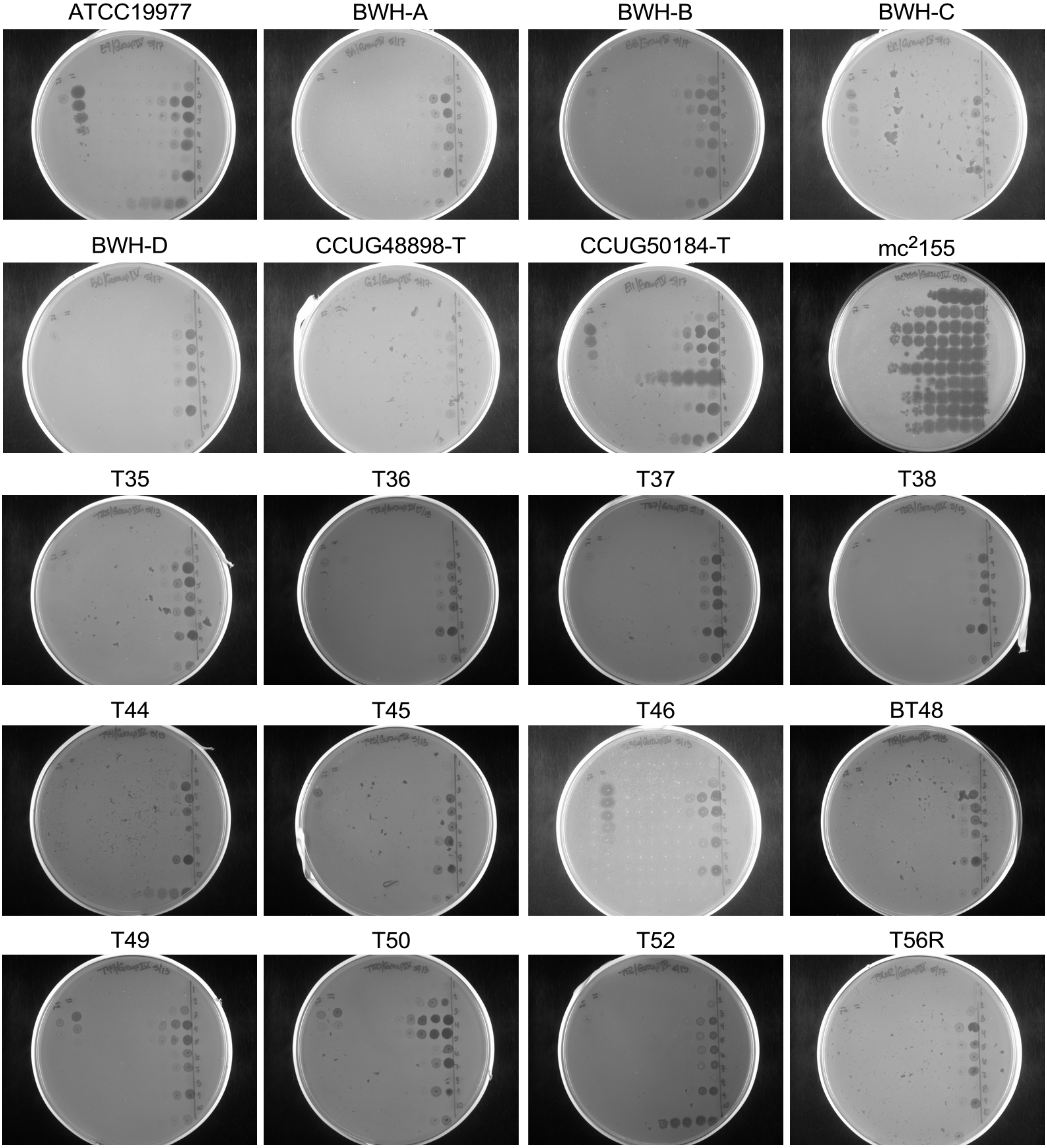

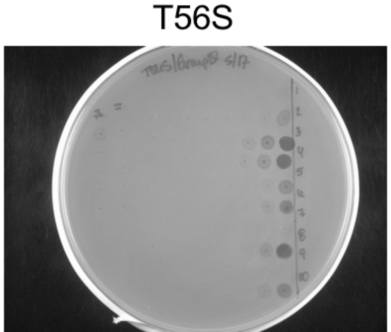

Group IV Bacteriophages (cluster)
1. D29 (A2)
2. Fionnbharth WT (K4)
3. Fionnbharth ΔΔ (K4)
4. Fionnbharth ΔΔ_K1toxin (K4)
5. MissWhite (A2)
6. Fred313Δrep (A3)
7. Adzzy (A2)
8. Trixie (A2)
9. Pukovnik (A2)
10. Starstuff (A2)
11. phiT45-1 (MabH)
12. phiT46-1 (MabG)

S12 and S13 Fig. Raw images

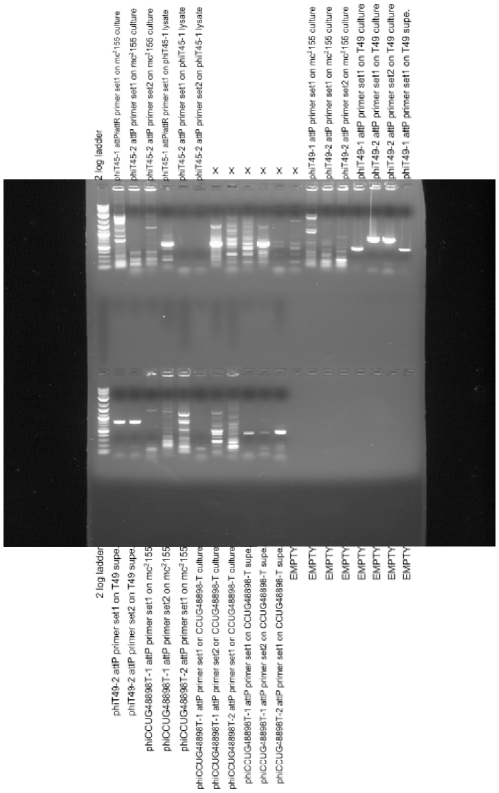

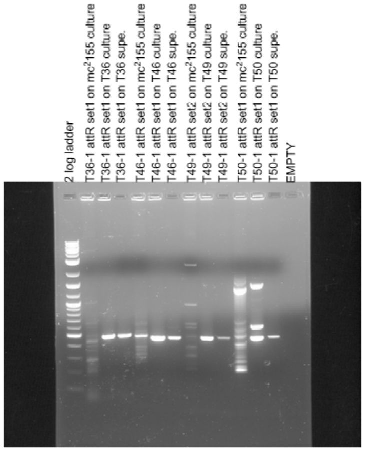

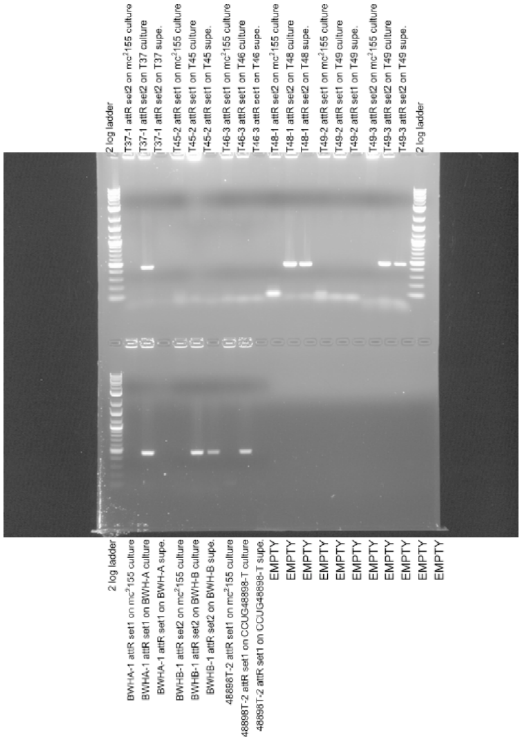

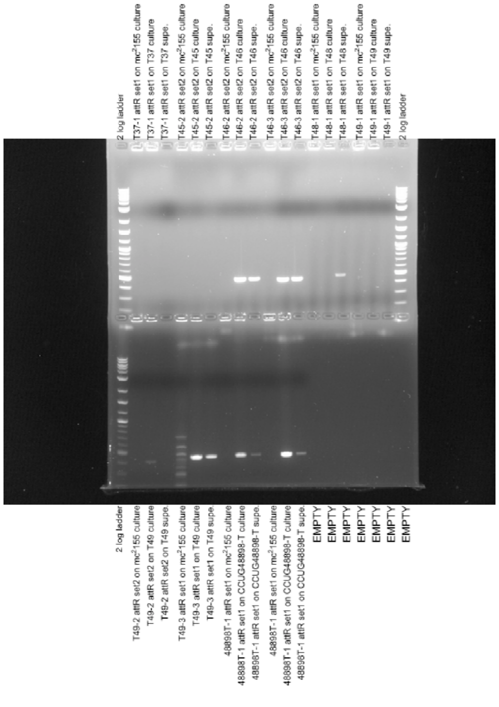

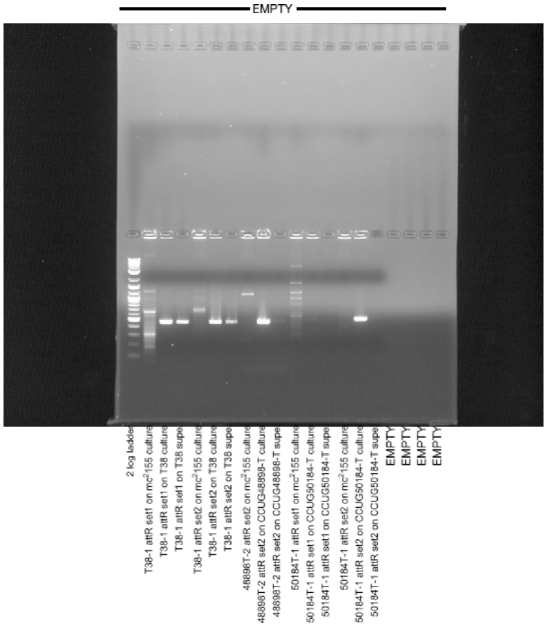

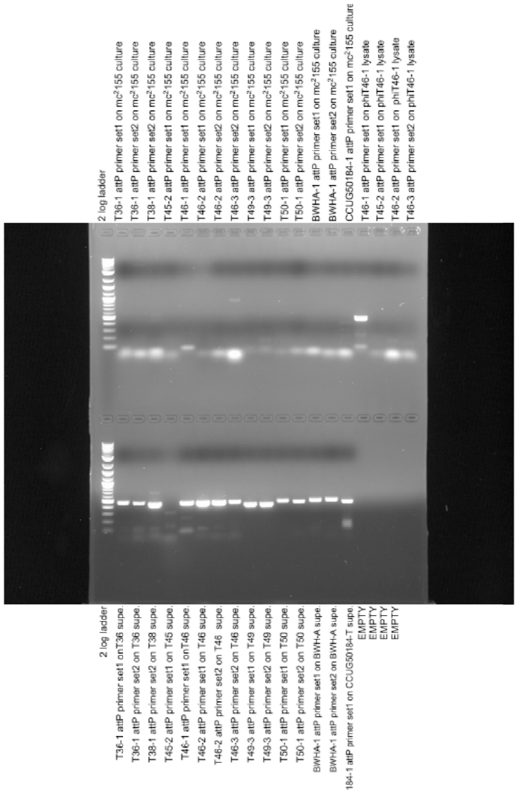

S12 and S13 Fig. Raw images

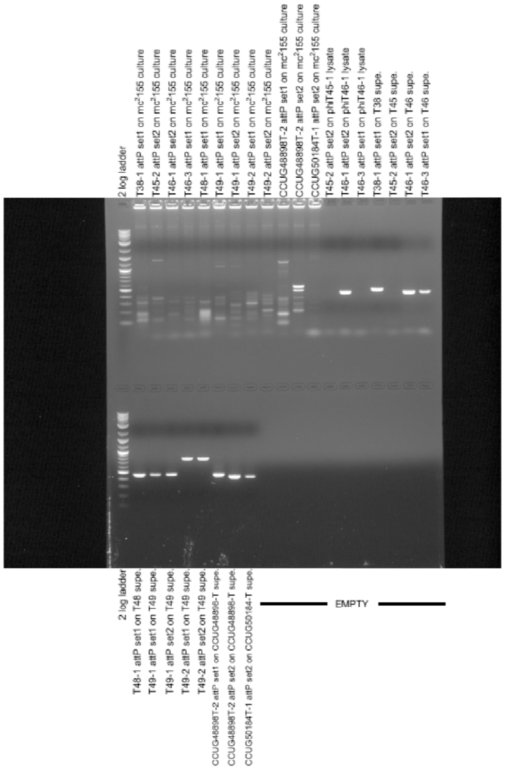

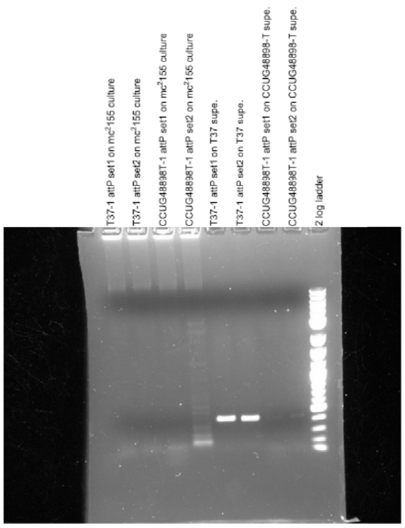

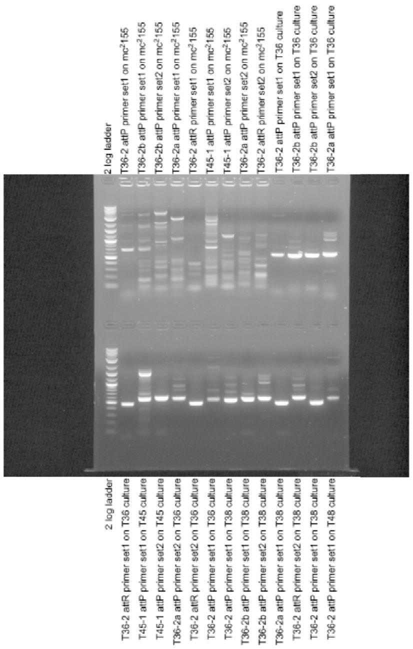

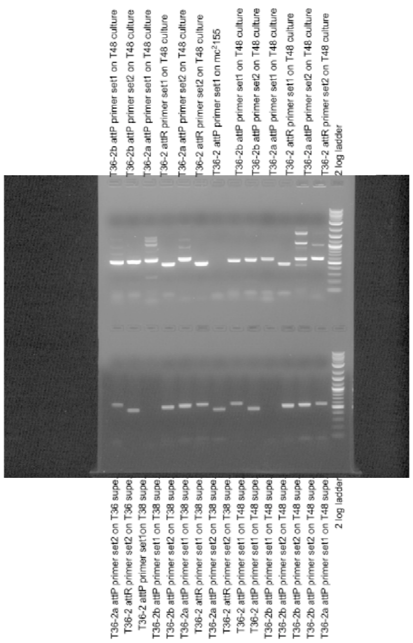

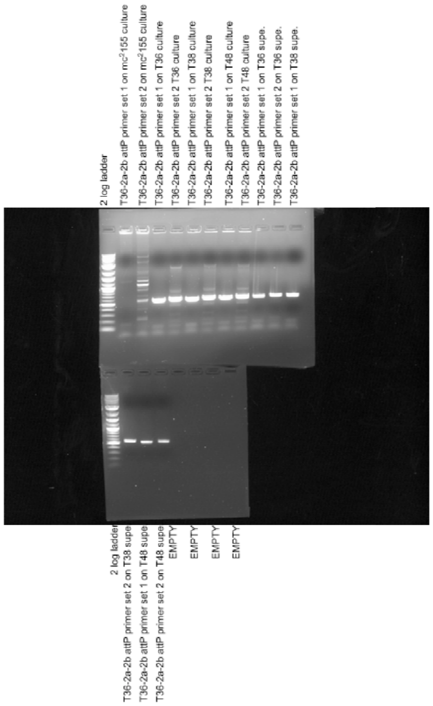

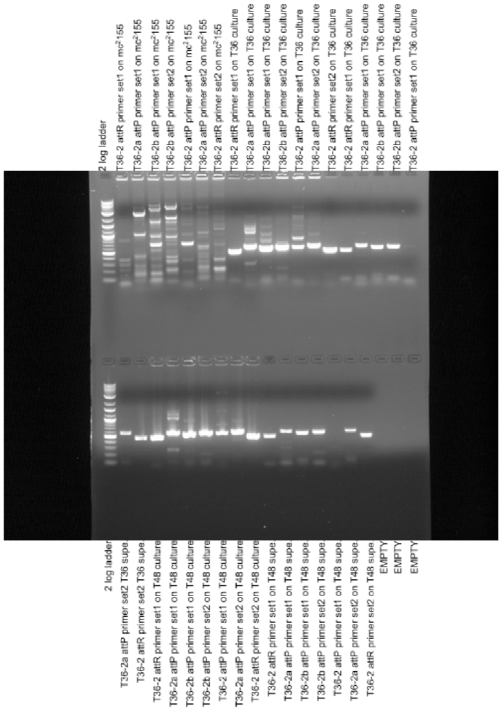

S12 and S13 Fig. Raw images

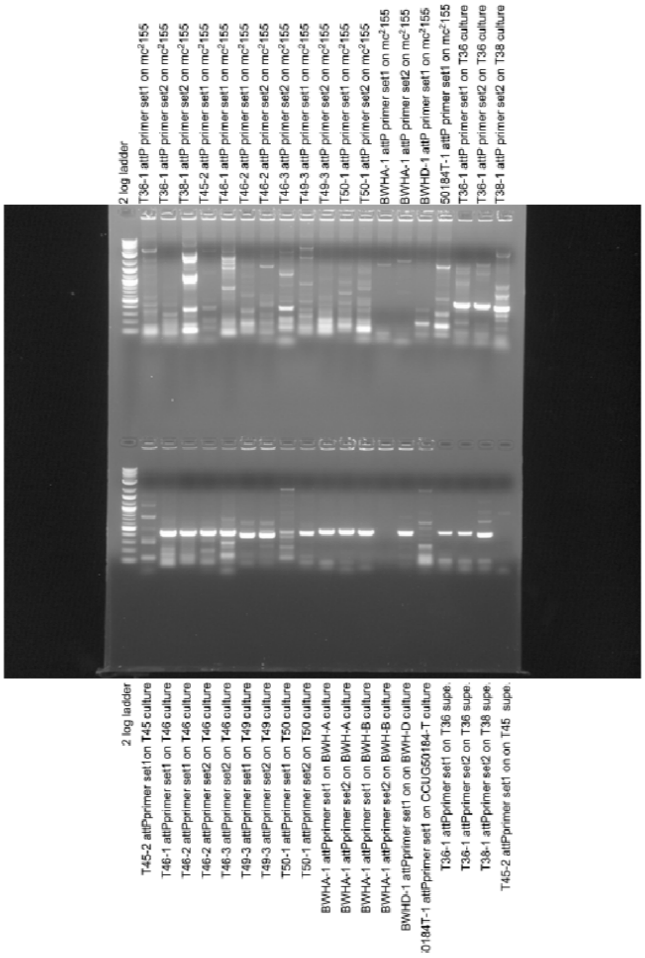

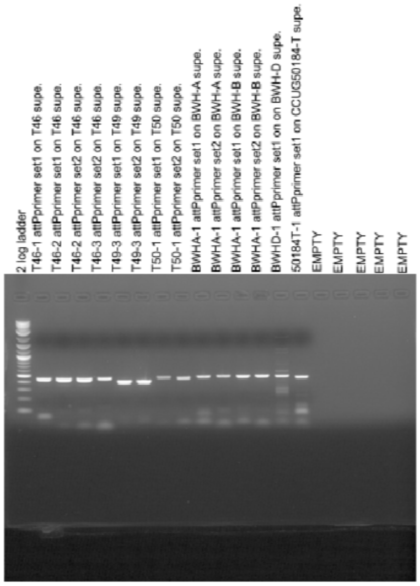

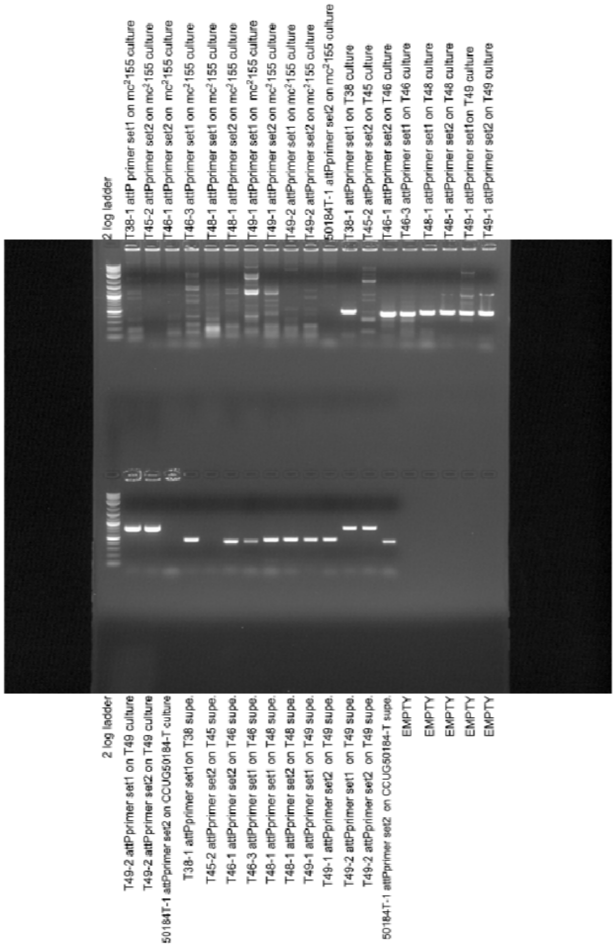

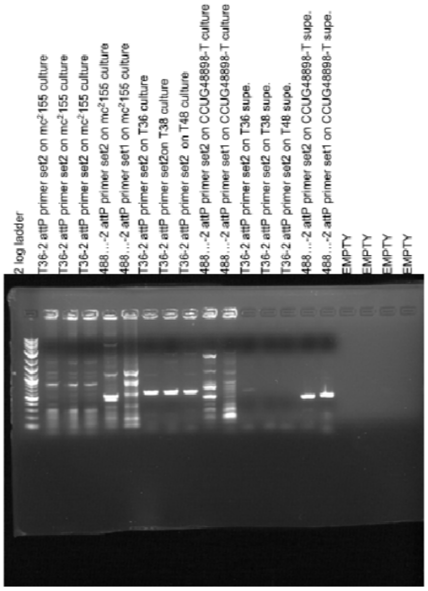

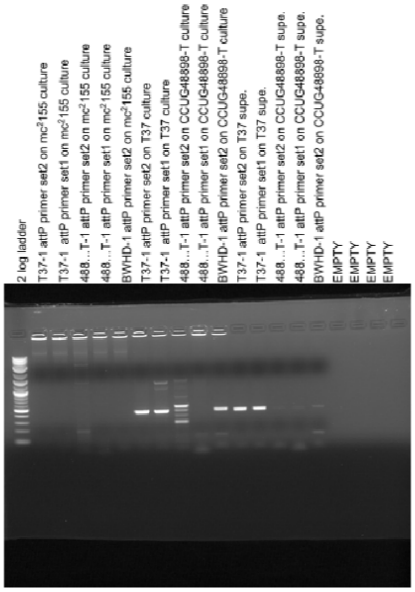

S12 and S13 Fig. Raw images

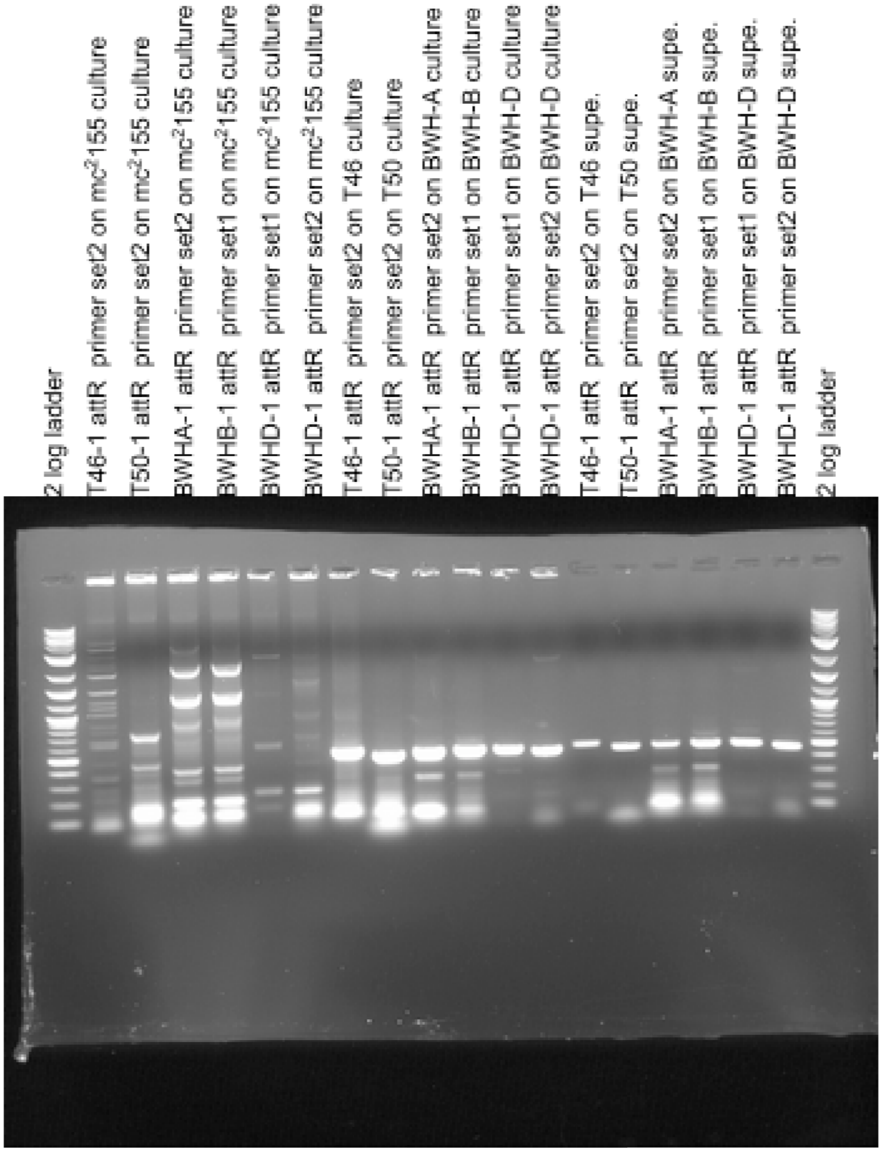

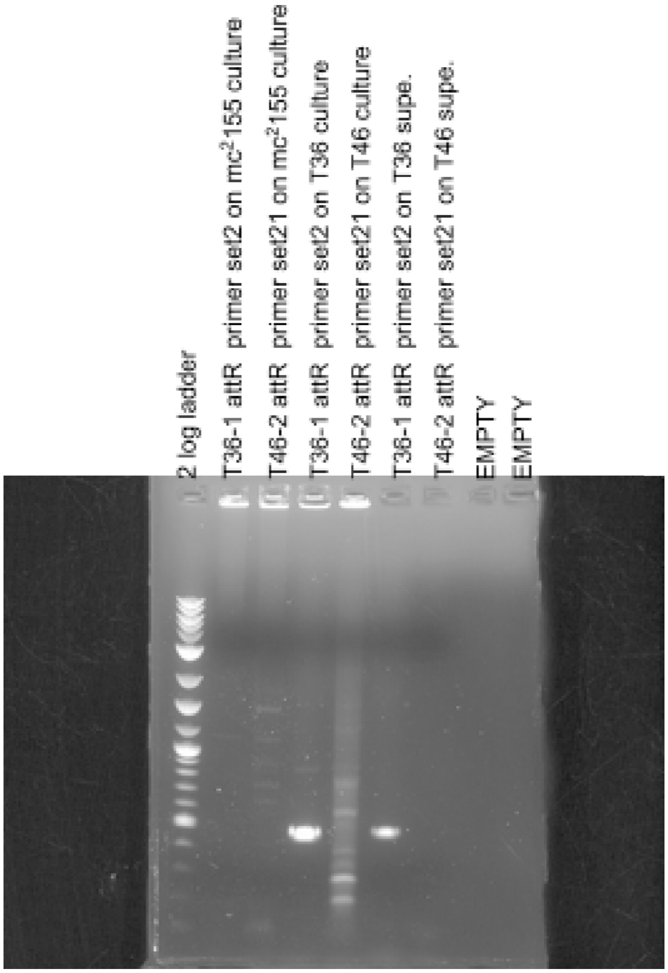

S14 and S15 Fig Raw Images

ATCC19977

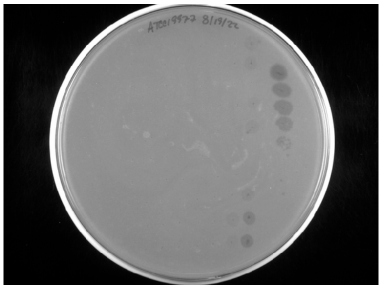

GD40

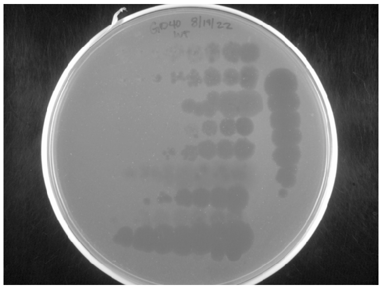

GD40(phiT45-1)

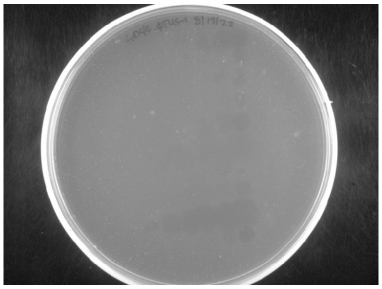

mc^2155

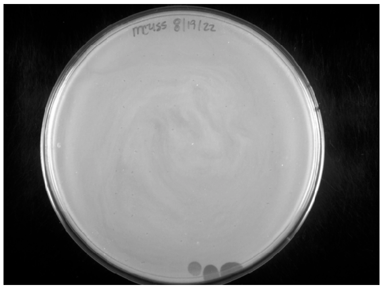

GD41

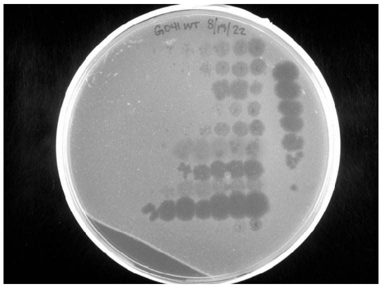

GD41(phiT45-1)

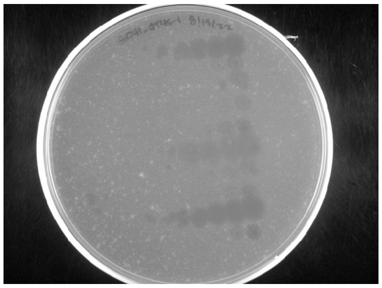

GD245

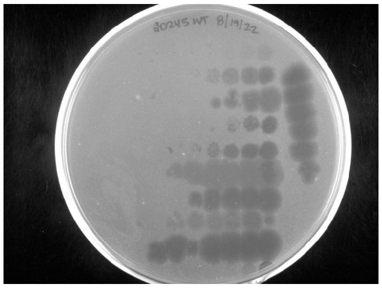

GD245(phiT45-1)

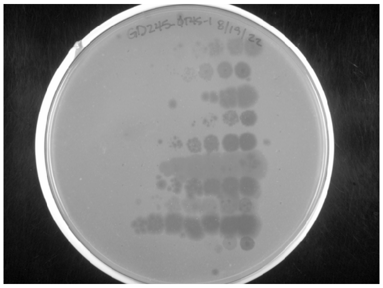

GD## (phiT45-1) Group I Key
Bacteriophage (cluster)

1. phiGD17-1 (MabD)
2. phiGD20-1 (MabA1)
3. phiGD21-1 (MabB)
4. phiGD22-1 (MabA1)
5. phiGD23-1 (MabA1)
6. phiGD24-3 (MabJ)
7. phiGD34-2 (MabB)
8. phiGD57-1 (MabC)
9. phiGD89-1 (MabB)
10. Muddy (AB)
11. phiT45-1 (MabH)

S14 and S15 Fig Raw Images

GD40

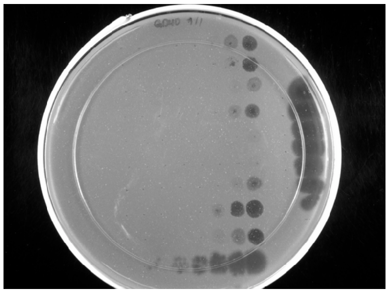

GD40(phiT45-1)

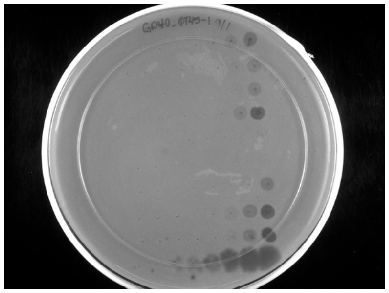

GD41

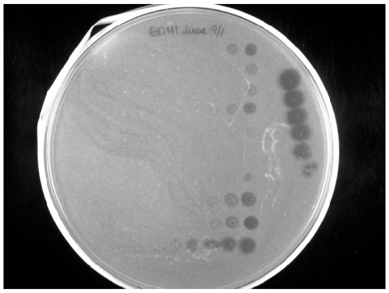

GD41(phiT45-1)

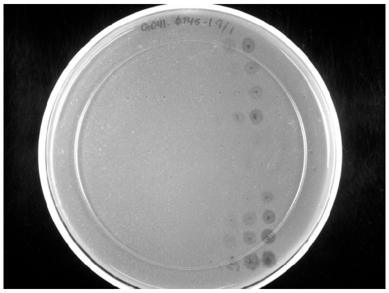

GD245

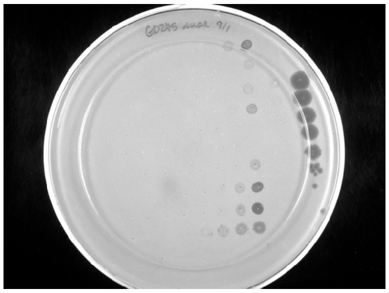

GD245(phiT45-1)

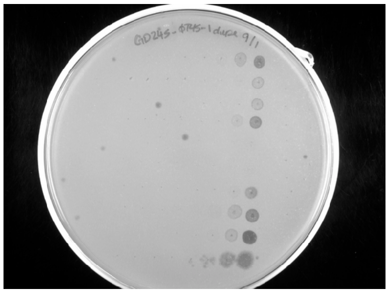

ATCC19977

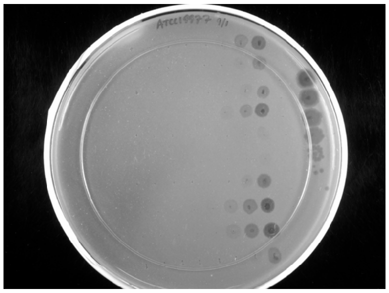

mc^2155

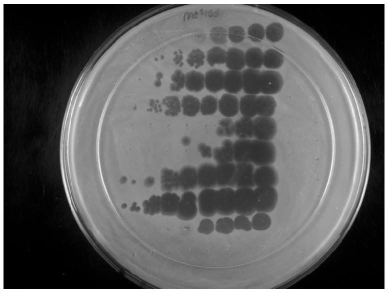

GD## (phiT45-1) Group I Key
Bacteriophage (cluster)

1. phiGD17-1 (MabD)
2. phiGD20-1 (MabA1)
3. phiGD21-1 (MabB)
4. phiGD22-1 (MabA1)
5. phiGD23-1 (MabA1)
6. phiGD24-3 (MabJ)
7. phiGD34-2 (MabB)
8. phiGD57-1 (MabC)
9. phiGD89-1 (MabB)
10. Muddy (AB)
11. phiT45-1 (MabH)
